# Mutational disparities in colorectal cancers of White Americans, Alabama African Americans, And Oklahoma American Indians

**DOI:** 10.1038/s41698-024-00782-9

**Published:** 2024-12-23

**Authors:** Hiroshi Y. Yamada, Madhusmita Rout, Chao Xu, Philip H. O’Neill, Farrukh Afaq, Katherine T. Morris, Dharambir K. Sanghera, Upender Manne, Chinthalapally V. Rao

**Affiliations:** 1 Department of Internal Medicine, Hematology/Oncology Section, University of Oklahoma Health Sciences Center (OUHSC), Oklahoma City, OK USA; 2 Center for Cancer Prevention and Drug Development, Stephenson Cancer Center, University of Oklahoma Health Sciences Center (OUHSC), Oklahoma City, OK USA; 3 Department of Pediatrics, College of Medicine, University of Oklahoma Health Sciences Center, 940 Stanton L. Young Blvd., Rm 317 BMSB, Oklahoma City, OK USA; 4 Department of Biostatistics and Epidemiology, Hudson College of Public Health, University of Oklahoma Health Sciences Center (OUHSC), Oklahoma City, OK USA; 5 Harold Hamm Diabetes Center, University of Oklahoma Health Sciences Center (OUHSC), Oklahoma City, OK USA; 6 Department of Pathology, Heersink School of Medicine, University of Alabama at Birmingham, Birmingham, AL USA; 7 Department of Surgery, University of Oklahoma Health Sciences Center (OUHSC), Oklahoma City, OK USA; 8 VA Medical Center, Oklahoma City, OK USA

**Keywords:** Colon cancer, Cancer genomics, Cancer genomics, Mutation

## Abstract

The high incidence and mortality rates of colorectal cancer (CRC) in Alabama African Americans (AAs) and Oklahoma American Indians (AIs) are recognized as cancer disparities, yet the underlying causes have been poorly demonstrated. By evaluating CRC whole-exome sequencing and mutational profiles, here we report sets of mutated genes whose frequencies differed significantly (*p* < 0.05) in a race-specific manner. Secondary screening with cancer database identified “survival-critical genes (SCGs)” (i.e., genes whose mutations/alterations are associated with significant differences in the patients’ survival rates) among the differentially mutated genes. Notable SCGs with race-pronounced variants were different from DEGs and their involved pathways included nucleotide catabolism and cell cycle checkpoints for AAs, and extracellular matrix organization for AIs. The inclusion of these SCGs with race-pronounced variants in the clinical CRC next-generation sequencing panels and the development of targeting drugs will serve as refinements for precision medicine to overcome racial disparities in health outcomes of CRC.

## Introduction

The practice of precision medicine utilizes profiling by next-generation sequencing (NGS) of cancer specimens from patients. The cancer NGS panels include a few hundred cancer-associated genes and mutations with proven clinical importance, such as K-RAS G12V and EGFR L858R, can be targeted by a matching specific inhibitor (i.e., a small molecule or engineered antibody). The precision medicine approach, using information on the distinctive cancer mutation profile and a specific targeting drug, has improved cancer outcomes.

However, the clinical NGS panels cover only select genes, due to cost and to insufficient knowledge of genes and pathogenic mutations involved in cancer. For example, the OncoKB website by Memorial Sloan Kettering (https://www.oncokb.org/)^1^ is consulted by clinicians working with precision medicine. The site is described as “MSK’s Precision Oncology Knowledge Base/An FDA-Recognized Human Genetic Variant Database.” Yet, the website lists only 894 genes, 7893 gene mutations, 141 cancer types, and 147 drugs (as of Oct 30, 2024)^1^. However, there are 20,000–22,000 protein-coding genes in the human genome, and numerous mutations have been reported. There is a need to improve the current practice of cancer precision medicine through expansion of the knowledge base for genes associated with cancer and by validating functional involvement of the mutations.

Colorectal cancer (CRC) is the second most common cause of cancer deaths in the US. For 2024, the American Cancer Society estimates 106,590 new cases of colon cancer (54,210 for men and 52,380 for women), and 46,220 new cases of rectal cancer (27,330 for men and 18,890 for women). The death toll due to CRC is expected to reach 53,010 in 2024 (American Cancer Society, key statistics for 2024, retrieved 2/8/2024)^2^. Although advancements in screening and treatments contribute to decreased death rates among older people, for people under 55, the death rate has increased about 1% per year since the mid-2000s^2^. Thus, mortality due to CRC remains a major issue.

Racial disparities in CRC are recognized as an issue to be addressed. Mokdad et al. ^3^ reported that areas with high numbers of African Americans (AAs) in Alabama and American Indians (AIs) in Oklahoma suffer high incidences and mortality from cancers of the lung, breast, kidney, and colorectum. Reports on cancer disparities at the state level agree with the existence of CRC disparities for AAs and AIs in these states^4,5^. To explain the disparities, issues in various domains (socio-economic, environmental, cultural, lifestyle) were proposed as risk factors^6^. However, many of the risk factors are difficult to address.

Biological differences contribute to cancer disparities (e.g., breast cancer^7^). As these differences can be addressed in personalized medicine and drugs at the clinical practice level, further investigations, by use of molecular analysis tools, are needed to uncover racial disparities of cancers that will help advance the goals of preventive care and precision therapy. With this reasoning, we posited that “ethnic/racial groups may share biological patterning that occurs as a result of social constructs, as well as dietary habitats, geographic locations, and other cultural practice and lifestyles”^8^. Then, we focused on race as a representative factor that bundles several hard-to-separate biological patterning influences and proceeded to analyze molecular disparities of CRCs among racial groups.

In our previous study, we procured CRC DNA/RNA samples from Alabama AAs, Oklahoma AIs, and white Caucasians. By analysis of transcriptomic profiles, we found, in each group, notable differentially expressed genes (DEGs). Since the results demonstrated different usages of oncogenic or tumor-suppressive signaling, the molecular disparities in CRC among racial groups justified further analysis^8^.

In the present study, we performed whole-exome sequencing using tumor and normal tissue specimens from CRC patients. Since exome sequencing covers most protein-coding genes and is not limited to known oncogenes/tumor-suppressors, it is useful to uncover previously unidentified cancer-influencing genes and pathogenic mutations that can be new additions to the existing NGS panels for precision medicine. Here, we report race group-pronounced genes with differential variant allele frequencies (VAF) for whites, Alabama AAs, and Oklahoma AIs, and discuss survival-critical genes (SCGs) (i.e., genes whose mutations/alterations are associated with differences in the survival rates of patients, based on human CRC databases^9–11^), suggesting that the mutations have a functional impact on cancer and influence survival for each group. Most of the identified race group-pronounced SCGs are under-investigated, although the SCGs may represent targets for CRC drugs. Addition of the SCGs and targeting drugs to current precision medicine would be helpful to overcome racial CRC disparities.

## Results

### Identification of race group-pronounced genes with differential variant allele frequencies in CRCs of whites, Alabama AAs, and Oklahoma AIs

**Fig. 1:**
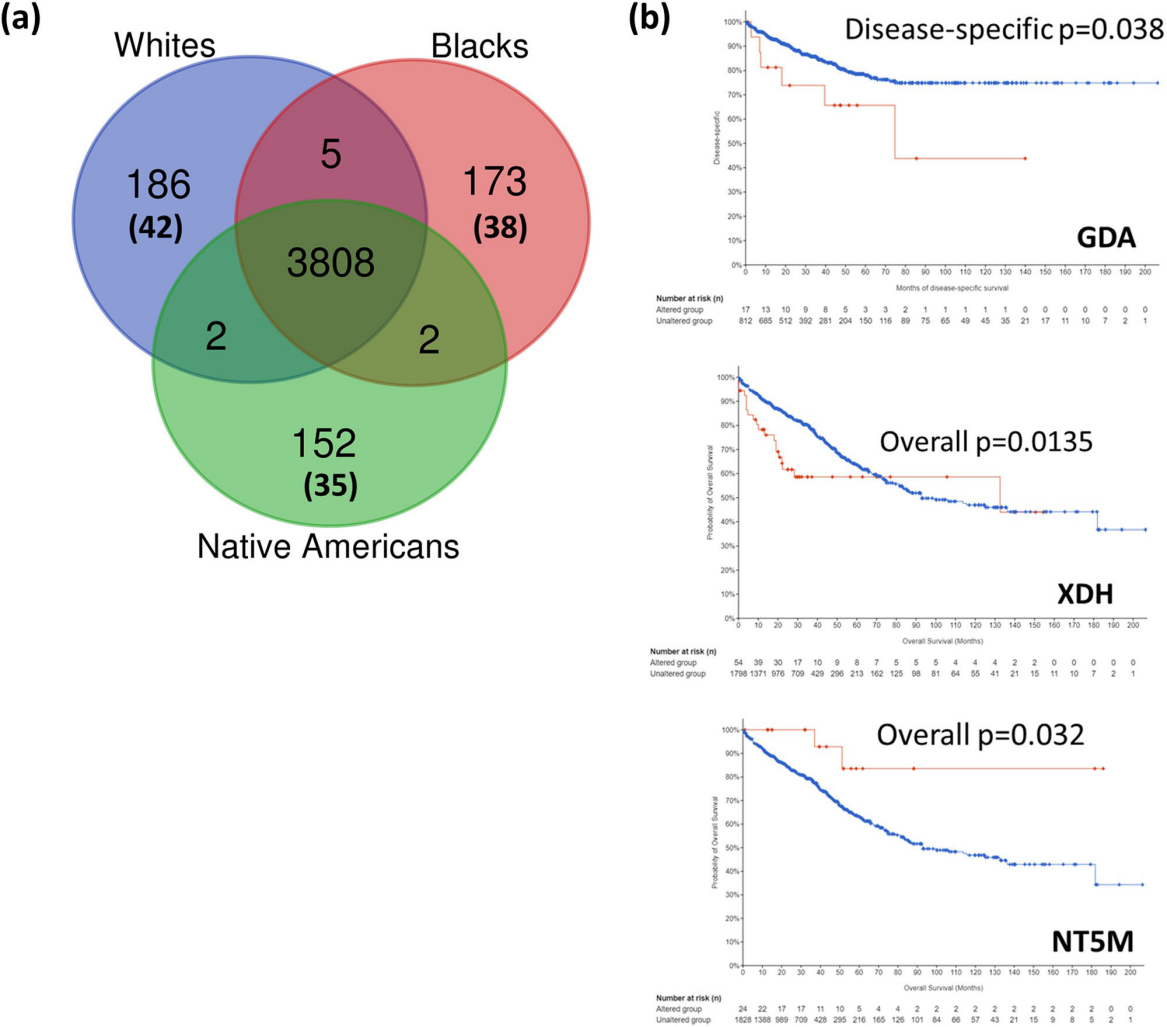
Genes with race-pronounced Variant Allele Frequencies in CRCs of white people, AAs, and AIs. **a** Venn diagram for Genes with Variant Allele Frequencies in CRCs of whites, AAs, and AIs. Variant detection and annotation analysis identified 186 genes with white CRC-pronounced variants, 173 AA genes with CRC-pronounced variants, and 153 genes with AI CRC-pronounced variants. A few variants occurred in a gene, or in a region not clearly annotated as a gene, resulting in the discrepancy between variant numbers in the Results section and gene numbers in the Venn diagram. Among the genes with race-pronounced variants, secondary analysis indicated that 42, 38, and 35 are SCGs, whose alterations are associated with differences in the survival rate of patients (*p* < 0.05). **b** Examples of SCGs. GDA, XDH, and NT5M were identified as SCGs in AA CRCs. Blue: unaltered. Red: altered. With GDA and XDH genes, alterations are associated with poorer survival. With the NT5M gene, alteration is associated with better overall survival.

We compared whole-exome sequencing data from CRCs of AA, AI, and white patients. The comparison analysis was limited to CRC; germline mutation analysis among races with non-diseased tissue was not covered. The variants were categorized based on the functional effect (UTRs, loss-of-function [LoF], missense, in-frame deletion/insertions, and introns). For white, AA, and AI CRCs, respectively, 200, 194, and 165 race group-pronounced variants were identified (*p* < 0.05) (Fig. 1a, Supplementary tables 1–3).

### For CRCs, genes with variant allele frequency differences did not correspond with DEGs

We hypothesized that the genes with variant allele frequency differences may be DEGs, and the profiles may represent similar profiles to previously identified DEGs. However, none of the genes with variant allele frequency differences showed > 2-fold gene expression differences (log2 fc >1 or < −1) for white CRCs or AA CRCs (Supplementary table 1–3). Among 165 genes with variant allele frequency differences for AI CRCs, only 3 showed >2-fold gene expression differences (SHANK1, PNKP, and PCDHGB7), and, in later analyses, none were identified as SCGs. Thus, genes with variant allele frequency differences were different from DEGs.

### Secondary screening for SCGs

Initial pathway analysis of white, AA, and AI genes with variant allele frequency differences did not show functional nodes in any of them. We attributed the outcome to inclusion of bystander genes whose mutations carry limited functional influence in cancer. To identify genes with variant allele frequency differences that are of functional importance to CRC, we consulted a CRC database for SCGs, i.e., genes whose mutations/alterations are associated with differences in the survival rates of patients (examples are shown in Fig. 1b)^9–11^. cBioportal, originally developed at Memorial Sloan Kettering Cancer Center, is an inclusive and continually updated database, compiling datasets from TCGA, GDC, GENIE and others as well as newly published studies. Thus, although cautionary interpretation is needed due to the nature of compiled datasets (e.g., data of newer analysis mode, such as exon sequencing or micro RNA, may be under-represented), cBioportal dataset is among the largest that are currently available. Another reason is that the cBioportal database comes with built-in analysis tools, including survival rate displays. The cBioportal database for “bowels cancer” included 19 studies, with 7661 CRC samples from 7435 patients (as of Jan-Feb, 2024). Of the 200, 194, and 165 genes with pronounced variants in white Caucasian, AA, and AI CRCs, respectively, 42, 38, and 35 genes were found to be SCGs (Table 1(a) white variant-SCGs, (b) AA variant-SCGs, (c) AI variant-SCGs, (d) type of variant in white, AA and AI). Notably, most variants are occurring in intron regions (Table 1(d)).

**Table 1:** Survival-Critical Genes (SCGs) with race-pronounced variant allele frequencies in CRC samples of White, African American and American Indian patients

(a) White CRC: SCGs with white-pronounced variants										
Identifier	Gene Names	Sequence Ontology	Effect	MAF	Beta	log2FoldChangeGeneExpression	*P*-Value	survival influence (*p* < 0.05)?	other notables *p* < 0.05	full gene name
rs3215471	SCGN	5_prime_UTR_variant	Other	0.40	−1.84	−0.05620146	3.54E-03	prog free *p* = 0.0343	MSI, TMB, race category	Secretagogin, EF-Hand Calcium Binding Protein
	GNG4	intron_variant	Other	0.22	18.95	−0.202686388	9.62E-03	prog free *p* = 0.0264	mutation count	G Protein Subunit Gamma 4
	LIPN	intron_variant	Other	0.31	19.01	−0.286501343	1.09E-02	prog free *p* = 0.0234	Race category, MSI,	Lipase Family Member N
rs12567713	FLVCR1	3_prime_UTR_variant	Other	0.20	18.66	−0.080004641	1.49E-02	Dis free *p* = 0.0484	MSI	FLVCR Choline And Heme Transporter 1
rs667627	FOXRED1	3_prime_UTR_variant	Other	0.20	18.66	0.090232312	1.49E-02	Prog free *p* = 0.0428	Race category, TMB, MSI, ICR high	FAD Dependent Oxidoreductase Domain Containing 1
rs6537	CHD1L	3_prime_UTR_variant	Other	0.40	19.01	−0.061599522	1.58E-02	Dis free *p* = 0.0476	TMB, MSI	Chromodomain Helicase DNA Binding Protein 1 Like
	NFASC	intron_variant	Other	0.22	19.56	−0.000640884	1.68E-02	Overall *p* = 0.0274	MSI, Race category, TMB, CIMP,	Neurofascin
	LOC112267934,PCDHA1,PCDHA2,PCDHA3,PCDHA4,PCDHA5,PCDHA6,PCDHA7,PCDHA8,PCDHA9,PCDHA10,PCDHA11	splice_region_variant	Other	0.30	19.12		1.92E-02	Prog free *p* = 0.0046	MSI, TMB, CIMP	Protocadherin gene cluster
	RGS1	upstream_gene_variant	Unknown	0.30	19.12	−0.324572117	1.92E-02	Dis free *p* = 0.00919	Race category, mut count	Regulator Of G Protein Signaling 1
	SFT2D1	intron_variant	Other	0.20	19.01	−0.020484766	1.96E-02	Overall *p* = 0.0103	Race category, MSI	SFT2 Domain Containing 1
	PLD1	intron_variant	Other	0.18	18.95	−0.138338021	2.18E-02	Dis free *p* = 0.0305	TMB, MSI,	Phospholipase D1
rs367544298	DNMT3A	intron_variant	Other	0.35	−1.30	0.107987337	2.25E-02	Overall *p* = 0.0217	MSI, race category, TMB, sex, ICR high, micro score,	DNA Methyltransferase 3 Alpha
	MYO1B	intron_variant	Other	0.25	19.46	−0.099018815	2.29E-02	Overall *p* = 0.00253	MSI, race category, TMB, CMS1	Myosin IB
	XDH	downstream_gene_variant	Unknown	0.25	19.46	−0.111715589	2.29E-02	Dis specific *p* = 0.0286	MSI, Race category, TMB, CIMP,	Xanthine Dehydrogenase
	DSCAML1	intron_variant	Other	0.25	19.01	0.074609586	2.29E-02	Overall *p* = 0.00065	MSI, TMB, CMS, CIMP,	DS Cell Adhesion Molecule Like 1
	PCDH10	3_prime_UTR_variant	Other	0.16	18.92	0.058149856	2.37E-02	Overall *p* = 0.0406	MSI, race category, TMB, CIMP, CMS, sex, ICR	Protocadherin 10
rs767542195	WDR35	intron_variant	Other	0.24	18.49	−0.202933257	2.41E-02	Dis free *p* = 0.0467	Race category, TMB, MSI,	WD Repeat Domain 35
	POLQ	intron_variant	Other	0.21	18.94	−0.252403164	2.55E-02	Overall *p* = 0.0491	MSI, race category, TMB, CMS1 and 4, CIMP	DNA Polymerase Theta
rs61738284	MKI67	missense_variant	Missense	0.17	18.66	−0.200139071	2.72E-02	Dis free *p* = 0.0453	MSI, race category, TMB, CIMP, ICR high, CMS1, sex	Marker Of Proliferation Ki-67
	MTHFSD	intron_variant	Other	0.19	18.89	0.076645439	2.75E-02	Prog free *p* = 0.0493	MSI, TMB	Methenyltetrahydrofolate Synthetase Domain Containing
	COL11A1	intron_variant	Other	0.18	18.66	−0.354833663	2.82E-02	Overall *p* = 0.0322	MSI, race category, TMB, CMS1, CIMP	Collagen Type XI Alpha 1 Chain
	MUC16	intron_variant	Other	0.33	18.76	−0.19095091	2.97E-02	Dis free *p* = 0.0295	MSI, race category, TMB, CMS1 and 3, ICR high,	Mucin 16, Cell Surface Associated
	FRYL	intron_variant	Other	0.33	19.01	−0.280691013	2.97E-02	Overall *p* = 0.045	MSI, race category, TMB, CMS1, CIMP,	FRY Like Transcription Coactivator
	FBXW12	intron_variant	Other	0.15	18.84	−0.243525124	3.03E-02	Prog free *p* = 0.0260	Race category, TMB	F-Box And WD Repeat Domain Containing 12
rs16971526	DNAH17	missense_variant	Missense	0.16	18.61	0.095539119	3.22E-02	Overall *p* = 0.00475	MSI, race category, TMB, CIMP, ICR high, CMS	Dynein Axonemal Heavy Chain 17
	INPP5D	intron_variant	Other	0.27	18.92	0.051346589	3.25E-02	Dis free *p* = 0.00259	Race category, TMB, MSI,	Inositol Polyphosphate-5-Phosphatase D
	DUOX1	intron_variant	Other	0.44	1.18	0.077735593	3.26E-02	Dis free *p* = 0.00272	MSI, TMB, race cetegory, CMS	Dual Oxidase 1
	CPS1	intron_variant	Other	0.18	18.80	−0.274611346	3.62E-02	Overall *p* = 0.0242	Race category, TMB, MSI,	Carbamoyl-Phosphate Synthase 1
	SALL2	3_prime_UTR_variant	Other	0.18	18.80	0.186086918	3.62E-02	Dis free *p* = 0.0452	MSI, race category, TMB, CIMP	Spalt Like Transcription Factor 2
rs11362069	DIDO1	intron_variant	Other	0.29	18.66	0.4624927	3.62E-02	Overall *p* = 0.003238	MSI, TMB, CIMP,	Death Inducer-Obliterator 1
	WDR62	intron_variant	Other	0.16	18.79	0.144751232	3.69E-02	Dis free *p* = 0.0300	MSI, race category, TMB, CIMP,	WD Repeat Domain 62
rs398000295	F13A1	splice_region_variant	Other	0.23	−1.13	−0.226005907	4.12E-02	Dis free *p* = 0.0281	Race category, TMB, MSI,	Coagulation Factor XIII A Chain
	FCGR2A	intron_variant	Other	0.15	18.72	0.126117921	4.50E-02	Dis free *p* = 0.0000429	MSI, TMB	Fc Gamma Receptor IIa
	DMGDH	intron_variant	Other	0.22	19.12	−0.160661248	4.58E-02	Prog free *p* = 0.0288	Race category, TMB, MSI, ICR high	Dimethylglycine Dehydrogenase
	MIOX	intron_variant	Other	0.22	19.12	0.346119458	4.58E-02	Overall *p* = 0.0122	MSI	Myo-Inositol Oxygenase
	NSD3	intron_variant	Other	0.19	18.74	−0.07335117	4.59E-02	Overall *p* = 0.0207	MSI, TMB, sex,	Nuclear Receptor Binding SET Domain Protein 3
rs11180815	NAP1L1	3_prime_UTR_variant	Other	0.20	18.53	−0.330809573	4.64E-02	Prog free *p* = 0.0381	Race category, TMB, MSI,	Nucleosome Assembly Protein 1 Like 1
	GDF6	downstream_gene_variant	Unknown	0.25	18.77	0.36400869	4.73E-02	Prog free *p* = 0.004	MSI, CIMP, ethinicity	Growth Differentiation Factor 6
	GLB1	intron_variant	Other	0.25	18.77	−0.056541977	4.73E-02	Overall *p* = 0.0218	Race category, TMB, MSI, CIMP	Galactosidase Beta 1
	ZMYND11	intron_variant	Other	0.25	18.77	−0.085319918	4.73E-02	Overall *p* = 0.0263	TMB, race category, MSI,	Zinc Finger MYND-Type Containing 11
rs546527484	ABCB1	intron_variant	Other	0.40	18.66	−0.129421568	4.83E-02	Overall *p* = 0.0007919	Race category, TMB, MSI,	ATP Binding Cassette Subfamily B Member 1
	LYAR	intron_variant	Other	0.30	18.80	0.000345182	4.83E-02	Prog free *p* = 0.0307, dis free *p* = 0.0471	Race category, TMB, MSI	Ly1 Antibody Reactive

(b) AA CRC: SCGs with AA-pronounced variants											
Predictor	Identifier	Gene Names	Sequence Ontology	Effect	MAF	Beta	log2FoldChangeGeneExpression	*P*-Value	survival influence (*p* < 0.05)?	other notables *p* < 0.05	full gene name
1:213178828-Ins		ANGEL2	intron_variant	Other	0.18	18.64	0.00131132	1.11E-02	Dis free *p* = 0.0263	Race category, MSI, TMB, CIMP	Angel Homolog 2
1:92573371-Ins		BTBD8	intron_variant	Other	0.18	19.12	0.17321944	1.22E-02	Overall *p* = 0.0274	Race category, TMB, Micro score	BTB Domain Containing 8
10:88556184-Ins		BMPR1A	intron_variant	Other	0.31	18.75	0.01988386	1.42E-02	Overall *p* = 0.0238	TMB, MSI, BMI	Bone Morphogenetic Protein Receptor Type 1A
11:117886314-Ins-2		SMIM35	intron_variant	Other	0.16	19.05	−0.0039115	1.44E-02	Prog free *p* = 0.009	Race category	Small Integral Membrane Protein 35
3:57269383-Ins		APPL1	intron_variant	Other	0.22	19.34	0.03505803	1.68E-02	Overall *p* = 0.0396	Race category, TMB, MSI, CMS1 and 3,	Adapter Protein, Phosphotyrosine Interacting With PH Domain And Leucine Zipper 1
17:37579508-Ins		MED1	intron_variant	Other	0.17	18.83	−0.0674786	1.76E-02	Overall *p* = 0.028	TMB, MSI,	Mediator Complex Subunit 1
5:71521792-Ins		MRPS27	intron_variant	Other	0.17	19.01	0.07325817	1.76E-02	Prog free *p* = 0.0235	Race category, TMB, MSI	Mitochondrial Ribosomal Protein S27
16:18839297-Ins		SMG1	intron_variant	Other	0.17	19.01	0.04762202	1.76E-02	Overall p = 0.0254	Race category, TMB, MSI, CMS1 and 3,	SMG1 Nonsense Mediated MRNA Decay Associated PI3K Related Kinase
19:37101428-Ins		ZNF382	intron_variant	Other	0.15	19.41	0.06689293	1.78E-02	Prog free *p* = 0.0439	Race category, TMB, MSI,	Zinc Finger Protein 382
1:175129682-Ins		KIAA0040	3_prime_UTR_variant	Other	0.20	19.01	−0.0335749	1.96E-02	Dis free *p* = 0.0022		KIAA0040
1:36068784-Ins-2		PSMB2	3_prime_UTR_variant	Other	0.20	19.01	0.15878608	1.96E-02	Overall *p* = 0.0253	MSI, TMB, CMS	Proteasome 20S Subunit Beta 2
17:7574340-Ins		TP53	intron_variant	Other	0.18	18.95	−0.0467685	2.18E-02	Overall *p* = 0.0000015	MSI, race category, CMS, CIMP,	Tumor Protein P53
20:62378693-Ins	rs1199903552	ZBTB46	intron_variant	Other	0.18	18.95	−0.0388824	2.18E-02	Overall *p* = 0.0276	MSI, CIMP	Zinc Finger And BTB Domain Containing 46
1:9005719-Ins		CA6	upstream_gene_variant	Unknown	0.25	19.46	0.03497811	2.29E-02	Dis free *p* = 0.0478	TMB	Carbonic Anhydrase 6
9:74828655-Ins		GDA	intron_variant	Other	0.25	19.46	0.10055252	2.29E-02	Dis specific *p* = 0.038	Race category, MSI, TMB	Guanine Deaminase
3:164704708-Ins		SI	intron_variant	Other	0.25	19.46	0.07352369	2.29E-02	Dis free *p* = 0.0365	Race category, TMB, CIMP,	Sucrase-Isomaltase
17:38142763-Ins		PSMD3	intron_variant	Other	0.16	18.92	−0.0139838	2.37E-02	Dis specific *p* = 0.0379	MSI, TMB,	Proteasome 26S Subunit, Non-ATPase 3
12:122818749-Del		CLIP1	intron_variant	Other	0.21	18.94	0.10104381	2.55E-02	Dis free *p* = 0.008663	Race category, TMB, MSI,	CAP-Gly Domain Containing Linker Protein 1
19:55589357-Ins		EPS8L1	intron_variant	Other	0.21	18.94	0.08528299	2.55E-02	Prog free *p* = 0.0492	Race category, MSI, TMB, CMS, CIMP	EPS8 Signaling Adapter L1
3:4741740-Del		ITPR1	intron_variant	Other	0.21	18.94	−0.0098828	2.55E-02	Dis free *p* = 0.0301	MSI, race category, TMB,	Inositol 1,4,5-Trisphosphate Receptor Type 1
19:50496360-Del		VRK3	intron_variant	Other	0.21	18.94	0.05629575	2.55E-02	Overall *p* = 0.00992	Race category, MSI, TMB	VRK Serine/Threonine Kinase 3
17:3840695-Del	rs34866939	ATP2A3	intron_variant	Other	0.30	1.15	−0.1656394	2.64E-02	Overall *p* = 0.0110	TMB, MSI, CIMP, CMS	ATPase Sarcoplasmic/Endoplasmic Reticulum Ca2+ Transporting 3
9:79319596-Ins		PRUNE2	intron_variant	Other	0.19	18.89	0.02486063	2.75E-02	Dis free *p* = 0.0274	MSI, race category, TMB, CIMP, CMS1, ICR high	Prune Homolog 2 With BCH Domain
16:67318608-Ins		PLEKHG4	splice_region_variant	Other	0.14	18.82	−0.0264875	3.13E-02	Overall *p* = 0.001095	Race category, TMB, MSI, ICR high, micro score	Pleckstrin Homology And RhoGEF Domain Containing G4
1:156087782-Del		LMNA	intron_variant	Other	0.27	18.92	−0.1318537	3.25E-02	Dis free *p* = 0.0236	Race category, MSI, TMB, CIMP, CMS	Lamin A/C
5:58272398-Ins		PDE4D	intron_variant	Other	0.27	18.92	0.07264981	3.25E-02	Overall *p* = 0.0258	TMB	Phosphodiesterase 4D
1:211923219-Ins		LPGAT1	3_prime_UTR_variant	Other	0.15	19.15	0.1328728	3.53E-02	Dis free *p* = 0.0159	MSI	Lysophosphatidylglycerol Acyltransferase 1
15:54630732-Ins		UNC13C	intron_variant	Other	0.16	18.79	0.05769984	3.69E-02	Overall *p* = 0.00152	Race category, TMB, MSI, CIMP, CMS	Unc-13 Homolog C
12:131451185-Ins		ADGRD1	intron_variant	Other	0.11	19.05	−0.0096823	4.09E-02	Overall *p* = 0.0460	TMB, MSI, CIMP,	Adhesion G Protein-Coupled Receptor D1
2:31606871-Del		XDH	intron_variant	Other	0.17	19.08	0.00091117	4.33E-02	Dis specific *p* = 0.0286	MSI, race category, TMB, CIMP	Xanthine Dehydrogenase
7:21744896-Ins		DNAH11	intron_variant	Other	0.17	18.73	0.13863206	4.54E-02	Dis free *p* = 0.0290	MSI, TMB, race category, CIMP, CMS,	Dynein Axonemal Heavy Chain 11
11:8942745-Ins		C11orf16	intron_variant	Other	0.22	19.12	−0.1367376	4.58E-02	Overall *p* = 0.0370	TMB	Chromosome 11 Open Reading Frame 16
12:57494010-Del		STAT6	intron_variant	Other	0.22	19.12	−0.0708193	4.58E-02	Prog free *p* = 0.0143, overall *p* = 0.0486	Race category, TMB, MSI, CMS, CIMP	Signal Transducer And Activator Of Transcription 6
17:17249950-Ins		NT5M	intron_variant	Other	0.25	18.77	−0.0246793	4.73E-02	Overall *p* = 0.0185	Race category, MSI	5’,3’-Nucleotidase, Mitochondrial
1:156531520-Ins-2		IQGAP3	intron_variant	Other	0.30	18.80	−0.1142018	4.83E-02	Overall *p* = 0.0433	MSI, race category, TMB, CIMP, CMS	IQ Motif Containing GTPase Activating Protein 3
12:104131673-Ins		STAB2	intron_variant	Other	0.30	18.80	0.07340242	4.83E-02	Dis free *p* = 0.003116, overall *p* = 0.0215	MSI, race category, TMB, CMS1,	Stabilin 2
6:109796433-Ins-2		ZBTB24	intron_variant	Other	0.30	18.80	0.00222211	4.83E-02	Overall *p* = 0.007818	Race category, TMB, MSI	Zinc Finger And BTB Domain Containing 24
1:174987485-Ins		MRPS14	intron_variant	Other	0.14	19.01	0.18557195	4.99E-02	Dis free *p* = 0.0130	mut count	Mitochondrial Ribosomal Protein S14

(c) AI CRC: SCGs with AI-pronounced variants											
Predictor	Identifier	Gene Names	Sequence Ontology	Effect	MAF	Beta	log2FoldChangeGeneExpression	*P*-Value	survival influence (*p* < 0.05)?	other notables *p* < 0.05	full gene name
5:112047955-Ins	rs72545070	APC	intron_variant	Other	0.32	1.56	−0.6689439	2.86E-03	overall 0.0231	MSI, Race category, TMB, CIMP, CMS	Adenomatosis Polyposis Coli
11:51516036-SNV	rs11246608	OR4C46	missense_variant	Missense	0.26	3.91	−0.52560157	3.00E-03	dis specific 0.0457	MSI, TMB, Race category,	Olfactory Receptor Family 4 Subfamily C Member 46
1:153926916-Del		CRTC2	intron_variant	Other	0.13	19.94	0.313932567	4.10E-03	Dis free 0.0480	MSI, TMB	CREB Regulated Transcription Coactivator 2
17:28811150-Ins		GOSR1	intron_variant	Other	0.13	19.65	−0.25347783	4.78E-03	Dis free 0.004, overall 0.0147	Race category, TMB	Golgi SNAP Receptor Complex Member 1
18:42646635-Del-2	rs34125334	SETBP1	3_prime_UTR_variant	Other	0.21	1.87	−0.0924814	5.49E-03	overall 0.0099	TMB, MSI, CMS1, ICR high, race category, CIMP	SET Binding Protein 1
2:51256013-SNV	rs62143026	NRXN1	5_prime_UTR_premature_start_codon_gain_variant	Missense	0.07	19.51	−0.3557083	9.26E-03	Dis free 0.0256	MSI, TMB, CIMP, Race category	Neurexin 1
3:68934434-SNV	rs4855535	TAFA4	5_prime_UTR_variant	Other	0.09	19.53	−0.48190021	9.78E-03	Dis free 0.00155	Race category, MSI	TAFA Chemokine Like Family Member 4
8:134308938-Del	rs36215434	NDRG1	intron_variant	Other	0.25	1.75	0.270232705	1.01E-02	Dis specific 0.00851	mut count	N-Myc Downstream Regulated 1
4:54139976-Del	rs150594118	SCFD2	intron_variant	Other	0.14	1.79	−0.33823649	1.54E-02	Overall *p* = 0.004482	Race category, TMB, MSI	Sec1 Family Domain Containing 2
15:55881107-SNV	rs1992237	PYGO1	5_prime_UTR_variant	Other	0.27	1.21	−0.30053686	1.66E-02	Prog free *p* = 0.0334	Race category, MSI, TMB, CIMP, sex	Pygopus Family PHD Finger 1
10:97366107-SNV	rs8758	ALDH18A1	3_prime_UTR_variant	Other	0.19	1.35	−0.53830009	1.66E-02	Overall *p* = 0.002169	TMB, race category, MSI, CMS1	Aldehyde Dehydrogenase 18 Family Member A1
22:23603475-Ins		BCR	intron_variant	Other	0.05	19.91	0.223184195	2.06E-02	Overall *p* = 0.0473	MSI, race category, TMB,	BCR Activator Of RhoGEF And GTPase
2:227915536-Ins		COL4A4	intron_variant	Other	0.05	19.91	−0.3356373	2.06E-02	Prog free *p* = 0.0211	Race category, TMB,	Collagen Type IV Alpha 4 Chain
2:44127040-Del		LRPPRC	intron_variant	Other	0.05	19.91	−0.61644278	2.06E-02	Prog free *p* = 0.0149	MSI, TMB, race category, CIMP, CMS,	Leucine Rich Pentatricopeptide Repeat Containing
1:72400727-Ins		NEGR1	intron_variant	Other	0.05	19.91	−0.30982241	2.06E-02	Overall *p* = 0.00346		Neuronal Growth Regulator 1
2:214204856-Ins-2		SPAG16	intron_variant	Other	0.05	19.97	−0.67605724	2.17E-02	Dis free *p* = 0.0344	TMB, MSI	Sperm Associated Antigen 16
8:99105387-Del	rs1282798097	ERICH5	intron_variant	Other	0.05	20.08	-0.13848266	2.44E-02	Dis specific *p* = 0.00284	mut count	Glutamate Rich 5
3:5257384-Ins		EDEM1	intron_variant	Other	0.06	20.01	−0.13901577	2.79E-02	Overall *p* = 0.002308	Race category, TMB, MSI, ICR high	ER Degradation Enhancing Alpha-Mannosidase Like Protein 1
6:129464881-Ins		LAMA2	intron_variant	Other	0.06	20.01	−0.5873067	2.79E-02	Dis free *p* = 0.0395	MSI, race category, TMB, CIMP	Laminin Subunit Alpha 2
6:162206708-Ins-2		PRKN	intron_variant	Other	0.06	20.01	−0.1064017	2.79E-02	Prog free *p* = 0.0037	Race category, TMB, MSI, sex,	Parkin RBR E3 Ubiquitin Protein Ligase
1:156764385-Ins		PRCC	intron_variant	Other	0.06	19.98	−0.1733402	3.00E-02	Prog free *p* = 0.0283	Race category, TMB, MSI,	Proline Rich Mitotic Checkpoint Control Factor
7:127963422-Ins		RBM28	intron_variant	Other	0.06	19.98	−0.00917593	3.00E-02	Dis specific *p* = 0.0128	Race category, MSI, TMB, CIMP	RNA Binding Motif Protein 28
7:87149830-Ins		ABCB1	intron_variant	Other	0.07	19.94	−0.30076228	3.24E-02	Overall *p* = 0.0007919	Race category, TMB, MSI,	ATP Binding Cassette Subfamily B Member 1
11:72141200-Ins		CLPB	intron_variant	Other	0.07	19.94	−0.11102142	3.24E-02	Overall *p* = 0.0245	Race category, TMB, MSI,	ClpB Family Mitochondrial Disaggregase
18:7034437-Ins		LAMA1	intron_variant	Other	0.07	19.94	−0.4036241	3.24E-02	Overall *p* = 0.0246	MSI, TMB, CMS, race category, CIMP, ICR high, micro score, sex,	Laminin Subunit Alpha 1
6:160888506-Ins		LPAL2	non_coding_exon_variant	Other	0.07	19.94	−0.57402913	3.24E-02	Overall *p* = 0.0005864		Lipoprotein(A) Like 2 (Pseudogene)
22:44286810-Ins		PNPLA5	intron_variant	Other	0.07	19.94	0.360257292	3.24E-02	Dis free *p* = 0.0446	Race category, TMB, MSI,	Patatin Like Phospholipase Domain Containing 5
10:38120454-Ins		ZNF248	3_prime_UTR_variant	Other	0.07	19.94	−0.49914159	3.24E-02	Overall *p* = 0.0186	TMB, race category, MSI,	Zinc Finger Protein 248
16:15839438-Del	rs1421049064	MYH11	intron_variant	Other	0.07	19.65	−0.17889679	3.53E-02	Overall *p* = 0.0482	MSI, race category, TMB, CIMP, CMS	Myosin Heavy Chain 11
6:72993631-Ins		RIMS1	intron_variant	Other	0.07	19.65	−0.23745293	3.53E-02	Overall *p* = 0.0000948	MSI, race category, TMB, CMS,	Regulating Synaptic Membrane Exocytosis 1
21:11021164-Ins	rs145115496	BAGE2	3_prime_UTR_variant	Other	0.17	1.45	−0.52943687	3.53E-02	Dis free *p* = 0.0491	Race category, sex, MSI	BAGE Family Member 2 (Pseudogene)
17:47780450-Del		SLC35B1	intron_variant	Other	0.05	19.76	0.044222516	3.99E-02	Overall *p* = 0.0476	Race category, TMB	Solute Carrier Family 35 Member B1
16:24941975-Ins		ARHGAP17	intron_variant	Other	0.09	19.76	0.025098443	4.74E-02	Dis free *p* = 0.006566	Race category, TMB, MSI, CMS, CIMP	Rho GTPase Activating Protein 17
2:127841378-Del		BIN1	5_prime_UTR_variant	Other	0.09	19.76	0.762904004	4.74E-02	Prog free *p* = 0.004467	Race category, TMB, MSI	Bridging Integrator 1
12:1021159-SNV	rs10849584	RAD52	3_prime_UTR_variant	Other	0.09	19.76	−0.12418819	4.74E-02	Overall *p* = 0.0457	Race category, TMB	RAD52 Homolog, DNA Repair Protein

(d) Variant type summary									
	Intron	3′-UTR	5′-UTR	Up G	Down G	missense	splice	others	Total
White	28	6	1	1	2	2	2	0	42
AA/black	33	3	0	1	0	0	1	0	38
AI/NA	24	5	3	0	0	1	0	2	35

(a) White-pronounced variant SCGs. Of 186 genes with race-pronounced variants in CRCs from white patients, 42 genes are identified as SCGs. (b) AA-pronounced variant SCGs. Of 173 genes with race-pronounced variants in AA patients, 38 are SCGs. (c) AI-pronounced variant SCGs. Of 153 genes with race-pronounced variants in AI patients, 35 are SCGs.

The genes are listed in the order of *p*-value from low to high (<0.05). Gene expression level compared with normal colon is shown in a log2FoldChange column, in which <−1 or >1 is determined a significant fold change. Listed in the “other notables” column are notable clinical attributes that show significant differences (*p* < 0.05) between the gene “altered” and “unaltered” CRCs; MSI: Microsatellite Instability status, TMB: Tumor Mutational Burden (nonsynonymous), ICR: immunologic constant of rejection score, CIMP: CpG island methylator phenotype, CMS: Consensus Molecular Subtypes. The clinical attributes are as defined in the cBioportal database. Brief notes for the gene function and numbers of PubMed publications, a surrogate score for cumulative research activity, are included in Supplementary Data (Excel Tables 1–3). (d) Variant type summary. In all groups, intron variants were most frequently found. (Intron: intron variant, 3′-UTR: 3-prime UTR variant, 5′-UTR: 5-prime UTR variant, Up G: Upstream Gene variant, Down G: Downstream Gene variant, missense: missense variant, splice: splice region variant).

Supplementary Table 4 shows the top 200 variants (3′ or 5′ UTRs, missense, or intronic), whose frequencies differed significantly between tumor vs. benign tissues. The functionality was assessed with GnomAD, CADD, and ClinVar. However, for Whites and African Americans, only a few remained after the Bonferroni correction, whereas ~68 variants for Native Americans showed FDR *p* values between 0.039 and 0.0022.

Red highlighted text indicates that the mutation (SNP) creates the binding sites for both miRNA and transcription factors; the yellow highlighted text is the binding site for either a transcription factor or a miRNA.

### White CRC variant-pronounced SCGs

**Fig. 2:**
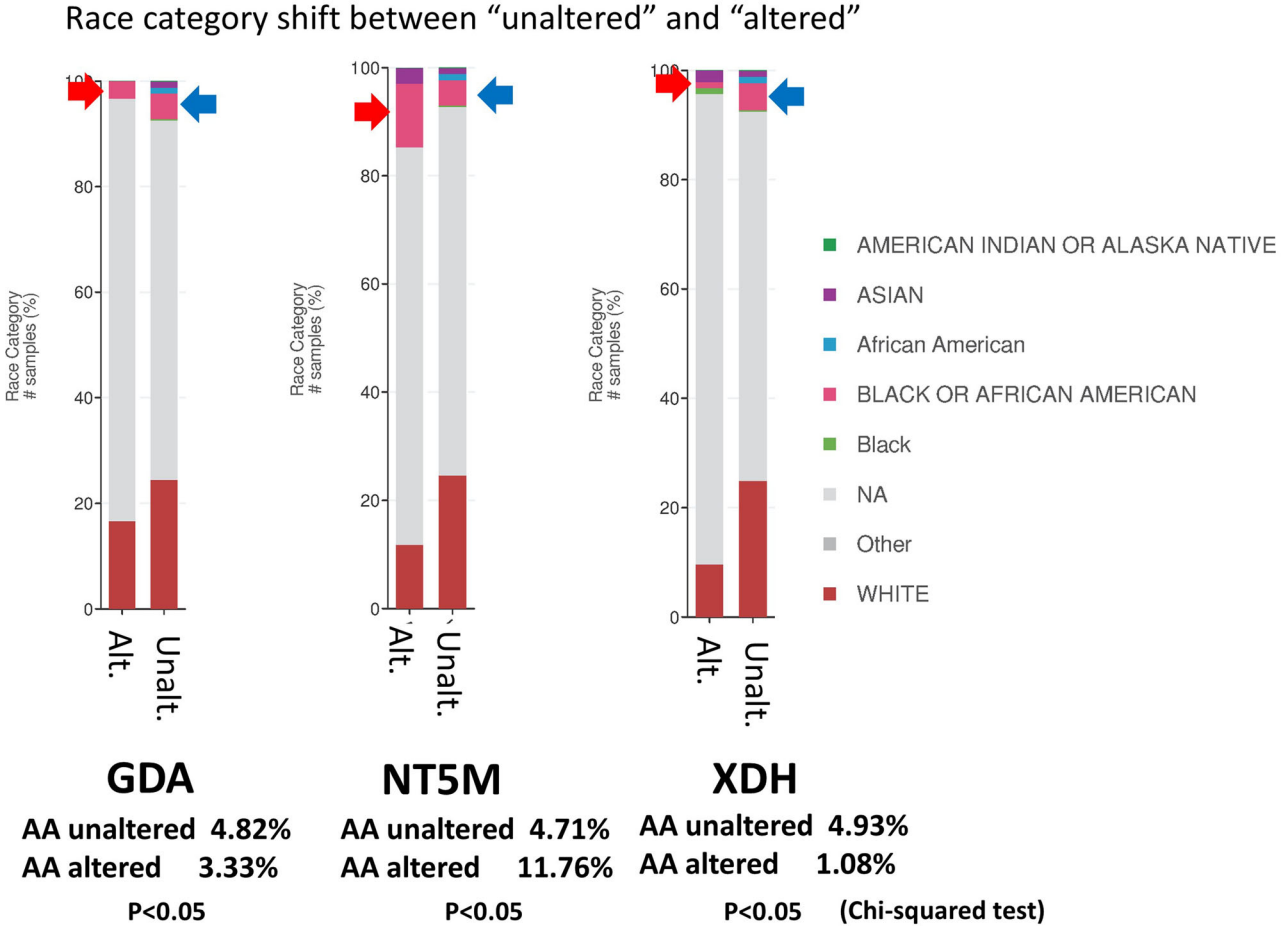
GDA, XDH, and NT5M gene alterations associated with race category. For CRCs with available race category information, the race category profiles were compared between GDA-, XDH-, or NT5M-altered CRCs and unaltered CRCs. Significant differences (chi-square analysis (*p* < 0.05)) among race categories between altered and unaltered CRCs suggest that the gene is involved in a race group-specific manner, confirming results of this study in the larger pool of CRCs in the database.

(shown in Table 1(a)). Pathway analysis of the 42 genes did not show a significant enrichment in a pathway (B-H *p* < 0.05). However, among the 42 SCGs, an association of a gene alteration with a race category was found for 29 genes, according to the cBioportal database, suggesting that most (69.0%) of the white variant-pronounced SCGs may be involved in an ethnicity-specific role for survival (examples of race category changes for AAs are shown in Fig. 2.). Other clinical attributes associated with all 42 of the SCGs (100%) were TMB/MSI/mutational counts, suggesting that the SCGs may be involved in genomic instability and/or a mutator phenotype.

Notable SCGs were identified with functions in nucleic acid metabolism (CHD1L, XDH, POLQ, MTHFSD, NAP1L1), DNA methylation (DNMT3A), the mucosal barrier (MUC16), transcription (SALL2, LYAR), the cell cycle (MKI67, FBXW12), multidrug resistance-associated drug pump (ABCB1), and tumor suppressor for other types of cancers (DIDO1). Alterations in 14 SCGs were associated with CIMP (CpG island methylator phenotype), which may reflect global genome hypermethylation and switched-off tumor suppressor genes. Alterations in 10 SCGs (MYO1B, DSCAML1, PCDH10, POLQ, MKI67, COL11A1, MUC16, FRYL, DNAH17, DUOX1) were associated with changes of consensus molecular subtypes (CMS). As CMS are transcription-based and subcategorized to 4 types (CMS1 “Immune”, CMS2 “Canonical”, CMS3 “metabolic”, and CMS4 “mesenchymal”)^12,13^, the SCGs in CRCs may be involved in switching of transcriptional patterns and/or molecular type. In relation to tumor-immune cell interactions, 6 of the 10 CMS change-associated SCGs (MYO1B, POLQ, MKI67, COL11A1, MUC16, FRYL) were associated with a CMS1 “immune”-type increase, and 6 (MYO1B, DSCAML1, PCDH10, MUC16, FRYL, DNAH17) may be involved in cell-cell interactions and/or motility, which facilitate immune infiltration.

Samples from Whites are best represented in existing CRC databases. As such, detailed analyses of Whites tend to be similar to existing analyses that do not distinguish racial groups (data not shown).

### Alabama AA CRC variant-pronounced SCGs

(Shown in Table 1 (b)). Pathway analysis for the 38 SCGs showed significant enrichment in 11 pathways (B-H *p* < 0.05); Guanosine Nucleotides Degradation III, Purine Nucleotides Degradation II (Aerobic), Nucleotide catabolism, Urate Biosynthesis/Inosine 5’-phosphate Degradation, Adenosine Nucleotides Degradation II (GDA, NT5M, XDH); BAG2 Signaling Pathway, Transcriptional regulation by RUNX3 (PSMB2, PSMD3, TP53); Mitotic Metaphase and Anaphase, Cell Cycle Checkpoints, RHO GTPases activate IQGAPs (CLIP1, LMNA, PSMB2, PSMD3, TP53, IQGAP3); and Huntington’s Disease Signaling (ITPR1, PSMB2, PSMD3, TP53). Of the 38 SCGs, associations of gene alterations with race category were found for 25 genes (65.8%). MSI/TMB/mutational counts were associated with 36 SCGs (94.7%). With exceptions of 7 genes with well-demonstrated involvements in CRCs (>10 CRC-related publications; i.e., BMPR1A, MED1, TP53, SI, ATP2A3, LMNA, STAT6), most (31 of 38) of the AA-variant SCGs were under-reported with limited numbers of publications (Supplementary Table 2).

**Fig. 3:**
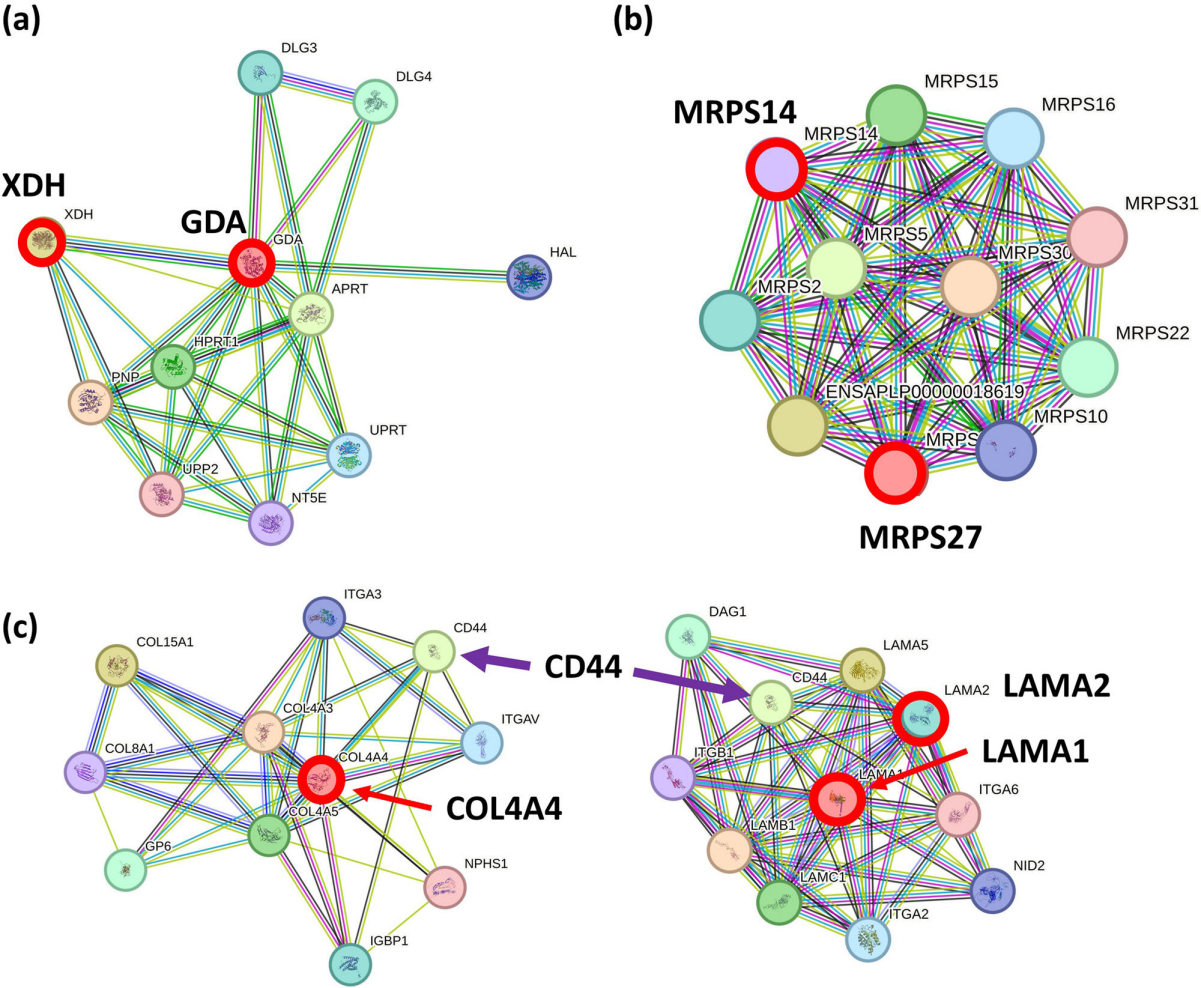
Protein interaction map in identified Pathways. **a** GDA-XDH in AA CRC-pronounced variant SCG. Both are involved in purine metabolism. **b** MRPS14 and MRPS27 in AA CRC-pronounced variant SCG. Both are mitochondrial ribosome subunits, implicating mitochondrial fitness affected in AA CRCs. **c** COL4A4, LAMA1, and LAMA2 in AI CRC-pronounced variant SCG are components of a network made of integrins and CD44. In CRCs, CD44 is a cancer stem cell marker^16^.

Notable AA CRC pronounced variant-SCGs included (a) GDA (Guanine Deaminase) and XDH (Xanthine Dehydrogenase), involved in nucleic acid and purine metabolism. Specifically, GDA catalyzes the hydrolytic deamination of guanine, producing xanthine and ammonia. XDH is a key enzyme in purine degradation. (Fig. 3a; Supplementary Fig. 1(b) MRPS27 and MRPS14 are mitochondrial ribosome subunits. Intron variants in these genes may influence mitochondrial ribosome stoichiometry and cause dysfunctional mitochondria. (Fig. 3b, c) PSMB2 and PSMD3 are 26S proteasome subunits for ubiquitin-mediated proteolysis. (d) BMPR1A (Bone Morphogenetic Protein Receptor Type 1A); its associated signaling demonstrates involvement in CRC development. For example, disease-causing variants in BMPR1a are present in juvenile polyposis syndrome^14^. Alterations in BMPR1A are also associated with BMI (Body Mass Index). (e) An intron variant in tumor suppressor, TP53. (f) An intron variant in STAT6 (Signal Transducer And Activator Of Transcription 6), which transduces IL4 and IL13-mediated signaling, regulates the expression of genes involved in the immune response, cell survival, and tumor proliferation and metastasis, hence, it has oncogenic functions in CRC^15^. (g) ANGEL2 (Angel Homolog 2), BTBD8 (BTB domain containing 8), and SMIM35 (Small Integral Membrane Protein 35), have the lowest *p*-values of *p* = 0.0111, 0.0122, and 0.0144, respectively. Yet, they are under-investigated genes with limited characterization.

Alterations in 14 SCGs (ANGEL2, TP53, ZBTB46, SI, EPS8L1, ATP2A3, PRUNE2, LMNA, UNC13C, ADGRD1, XDH, DNAH11, STAT6, IQGAP3) were associated with CIMP, and there were 13 SCGs with altered CMS (APPL1, SMG1, PSMB2, TP53, EPS8L1, ATP2A3, PRUNE2, LMNA, UNC13C, DNAH11, STAT6, IQGAP3, STAB2).

### Oklahoma AI CRC variant-pronounced SCGs

(shown in Table 1(c)). The 35 AI SCG list is a mix of (i) well-known CRC-related genes (APC, NDRG1, ABCB1), (ii) known oncogene/tumor suppressor/cancer-influencing genes in other types of cancers but not well-investigated in CRC (CRTC2, SETBP1, NRXN1, COL4A4, LRPPRC, NEGR1, EDEM1, LAMA2, PRKN, PRCC, CLPB, LAMA1, MYH11, RIMS1, RAD52), and (iii) under-investigated genes (the remaining 17 genes).

Pathway analysis on the 35 SCGs identified enrichment in one pathway, extracellular matrix organization (COL4A4, LAMA1, LAMA2, NRXN1). These genes are likely involved in the tumor microenvironment. COL4A4 encodes type IV collagen, interacting with integrin family proteins involved in cell-cell interaction as surface receptors (e.g., CD44, ITGA3, ITGAV). LAMA1 and LAMA2 are laminin subunits that interact with integrins (e.g., CD44, ITGA2, ITGA6). Together, COL4A4, LAMA1, and LAMA2 function to form the basement membrane structure. Shared interacting protein CD44 is a cancer stem cell marker for CRC^16^ (Fig. 3c). NRXN1 (neurexin) normally functions as a synapse-organizing molecule, yet NRXN1 can act as a receptor for cell-cell interactions, and expression is highly related to recurrence of gastric cancers. Consistently, patients with mutated NRXN1 showed better prognoses^17^.

Of the 35 SCGs, association of gene alteration with race category was evident for 29 genes (82.9%), again suggesting race-specific involvements of the variant-pronounced SCGs in CRCs. MSI/TMB/mutational counts were associated with 33 SCGs (94.3%). Alterations in 10 SCGs (APC, SETBP1, NRXN1, PYGO1, LRPPRC, LAMA2, ABCB1, LAMA1, MYH11, ARHGAP17) were associated with CIMP, and 8 SCGs, CMS (APC, SETBP1, ALDH18A1, LRPPRC, LAMA1, MYH11, RIMS1, ARHGAP17).

Notable AI variant SCGs include: (a) An intron variant of canonical tumor suppressor APC (Adenomatous Polyposis Coli). (b) An intron variant of ABCB1 (ATP Binding Cassette Subfamily B Member 1), a multi-drug pump involved in drug resistance to cancers. (c) SETBP1 (SET Binding Protein 1), which binds to the SET nuclear oncogene involved in DNA replication. SETBP1 mutations are a diagnostic marker for hematological malignancies^1^. SETBP1 mutations are also found in solid tumors^18^. In CRC cells, SET-SETBP1 and PP2A form a heterotrimeric complex^19^. However, the impact of the SETBP1 mutation in CRC has not been demonstrated. (d) LRPPRC (Leucine Rich Pentatricopeptide Repeat Containing) has multiple functions that can influence cancer. LRPPRC binds to CDK6-mRNA, increasing the stability and expression of CDK6. The LRPPRC-STAT3- SLIRP heterotrimeric complex is required for the stability of mature, mitochondrially encoded mRNAs and for transport to the mitochondrial ribosome^20^. LRPPRC is proposed to be a pan-cancer prognostic and immune biomarker^21^.

As many of the race-pronounced variant SCGs are associated with CMS distribution changes, we analyzed CMS distribution among race groups. However, most of the samples were not predicted to be in any class, and there were no significant differences in the distribution of CMS between race groups (data not shown).

## Discussion

Variant analysis focusing on CRCs from the three racial groups showed three sets of SCGs with race-pronounced variants. These differential variant SCG genes are different from DEGs identified in a previous study. Re-analysis of transcriptome data revealed that most of the mutated genes did not show significant gene expression-level changes, suggesting that up- or down-regulations of DEGs are mostly by posttranslational and/or epigenetic events rather than by exon mutations. The results showed that, in CRCs, modulations of gene expression and race-pronounced exon mutations are generally independent events.

As shown in Table 1(d), variants in introns were most frequently observed, implicating a role of these variants that may have eluded mRNA sequencing of previous generations. Whether an intron variant identified in this study can influence the function of a gene requires further case-by-case investigation. However, many among the listed intronic variants were 3′UTR variants, which create miRNA binding sites influencing protein expression or stability. Other intronic variants found in our data are predicted to create or abolish transcription factor binding sites (Supplementary Tables 4, 5).

Moreover, reports suggest that introns influence gene expression through interaction with splicing machinery^22,23^. Mechanistically, an intron mutation can result in retention of introns in mature transcripts, disrupting open reading frame and gene function. Intron mutations can be pathogenic, as for vacuolar sorting protein 13 gene (VPS13) variants of spastic ataxia patients^24^. In the case of VPS13 intronic variants, they caused altered splicing.

Involvements of splicing machinery in cancer are emerging (e.g., refs. ^22,23^). Our previous analysis of race group DEGs identified higher expressions of splicing machinery components in AIs^8^. Hence, there are likely under-investigated intron variants that interfere with protein functions, which, directly or indirectly, influence susceptibility to cancer.

Whether the mutation is a cancer-influencing pathogenic variant or is benign is a point that needs to be determined through validation studies. With sequencing data alone, the mutations remain as variants of uncertain significance (VUS). To aid evaluation, we consulted a secondary CRC database to identify genes of probable significance, i.e., SCGs. Analyzing whether a gene of interest is an SCG has been utilized as a rationale in various hypothesis-driven studies. With accumulating cancer datasets, SCG analysis is a data-driven approach to assess functional significance in cancer biology. Although the effects of a particular variant still need to be proven, SCG analysis is a viable method to identify functionally significant genes in the context of cancer from the standpoint of survival of patients, a clinically relevant parameter^25,26^.

The database consultation also showed that alterations in most of the SCGs with race-pronounced variants identified in this study are associated with a racial category, suggesting that the SCGs with race-pronounced variants are involved in an ethnicity-specific manner. Although human biology has revealed various racial differences at the molecular level, including DNA sequence and DEGs, their roles remain largely unidentified. These molecular differences can be exploited for disease preventive, diagnostic, and/or therapeutic purposes. The present study adds another proof-of-principle to investigate further the molecular racial differences toward clinical improvements. The approach has merits for racial minority patients, at least for investigation of diseased tissues in the context of personalized medicine.

Procuring human tissue samples for molecular investigation in the context of racial differences has met concerns and reluctance on donors, especially with AIs, in this study. Research engaging with AAs, AIs or members of other racial or ethnic minority groups must consider the potential for mistrust of the scientific community, arising from a long history of unethical research practices^27–29^. Moreover, many AIs are enrolled members of Tribes, which as domestic sovereign nations, have rights governing data use, data sharing and dissemination of findings that are unique among under-represented groups in the United States. Although this study was conducted in an NCI-designated state institute, if future research in precision medicine were to involve direct collaboration with Tribes or their healthcare delivery systems for tissue or other data acquisition, Tribal consultation and compliance with research protection protocols would be required.

White-pronounced variant SCGs did not show functional concentrations on pathways, but AA- and AI-pronounced variant SCGs did. Notably for AA CRCs, nucleic acid and purine metabolism (GDA, XDH) were pronounced. There are correlations in enzymes of the purine salvage pathway and clinical/biological aggressiveness of human colon carcinoma^30^. For pancreatic ductal adenocarcinoma, the CD37-dependent adenosine pathway inhibits immune cells, leading to immune suppression and implicating that products of the nucleotide metabolism pathway function as immune regulators and promote cancer immuno-surveillance. For *Drosophila*, XDH has a protective effect with respect to generation of reactive oxygen species and nitric oxide, particularly in the gut^31,32^. This pathway may serve as a druggable target.

Notable for AA CRCs is mitochondrial fitness, as suggested by mitochondrial ribosomal proteins (MRPS27 and MRPS14 variants). MRPS27 is an age-associated, differentially expressed gene. A homozygous variant in the MRPS14 (uS14m) was present in a patient with perinatal hypertrophic cardiomyopathy, growth retardation, muscle hypotonia, elevated lactate, dysmorphia, and mental retardation^33^. In skeletal muscle and fibroblasts from a patient, there was biochemical deficiency in complex IV of the respiratory chain^33^. Collectively, mitochondrial ribosomal proteins are involved in production of electron transport chain proteins, and malfunctions of mitochondrial ribosomes can result in metabolic disease^34^. Over the past decade, mitochondrial fitness has emerged as potential target for cancer therapy (e.g., refs. ^35,36^). As such, mitochondrial fitness in AA CRCs is a potentially druggable target for further investigation.

In a previous study, Guda et al. ^37^ identified novel somatic mutations in HTR1F, FLCN, and EPHA6 exclusively in AA patients with colon cancer, and these mutations were associated with adverse clinical outcomes. We checked HTR1F, FLCN, and EPHA6 from two viewpoints; (1) whether the genes are mutated (or identified as AA-pronounced variants) in our datasets, and (2) whether these genes are “survival critical” with the current database available at 2024. A difference from the 2015 study and 2024 study is the amount of accumulated cancer data, which affects evaluation of patients’ survival (“survival-critical”).

For (1): Our dataset did not identify HTR1F, FLCN, and EPHA6 as AA-pronounced variants. We speculate that the cause of the difference is patient population (i.e., the general AA population vs the Alabama-local AA population).

For (2): With consultation of data in the cBioportal bowel cancer (as of 9/5/2024, 24 studies, total 8611 samples), HTR1F and FLCN did not meet our “survival-critical gene” criteria. [i.e., HTR1F (overall survival *p* = 0.764, progression-free survival 0.497); FLCN (overall survival *p* = 0.607, disease-specific survival *p* = 0.131)]. However, EPHA6 showed progression-free survival (*p* = 0.02), meeting the criteria.

Hence, we note that the locality of patients’ population and cancer data size would be issues to consider when population-based studies from two different timepoints are compared.

AI-pronounced variant SCGs showed enrichment in one pathway: extracellular matrix organization (COL4A4, LAMA1, LAMA2, NRXN1). These proteins interact with integrins and, of particular interest, is CD44 (Fig. 3c). In CRCs, CD44 is a cancer stem cell marker^16^. Whether functions of the extracellular matrix organization pathway and interacting CRC stem cell marker CD44 and integrins are modulated in AI CRCs, allowing them to show differential responses to their targeting drugs, needs to be determined. In a previous DEG study, AI CRCs showed higher expression of PTGS2/COX2 and splicing regulators (RNU1-2, CLK1, CLK4)^8^. Intron variant of LRPPRC (Leucine-Rich Pentatricopeptide Repeat Containing) and PRCC (Proline-Rich Mitotic Checkpoint Control Factor) may partially explain abnormally high expressions of splicing regulators, as LRPPRC is an RNA-binding protein and functions in RNA polyadenylation, transport, and stability; it serves as a pan-cancer prognostic biomarker^21^. PRCC also may be involved in pre-mRNA splicing^38^. However, the lack of sufficient AI CRC samples is a limitation for advancing research in this area, a concern that should be addressed. For example, as of Sept 10, 2024, the cBioportal database carries 8625 bowel cancer samples, yet only 5 were identified as AIs. None was with exome sequencing or carried sufficient data parameters to compare directly with the dataset used in this study. This illustrates the paucity of molecular information.

As determined with the CRC database, alterations of the SCGs with race-pronounced variants are associated with TMB/MSI/mutation counts (100%, 94.7%, and 94.3% of race-pronounced variant SCGs in white, AA, and AI CRCs, respectively). Thus, most of the race-pronounced variant SCGs are functionally involved in the mutator phenotype or genomic instability. The fact that functions of most of the SCGs are understudied prevents us from investigating functions on genomic instability of the SCGs beyond correlation or association reports. However, the prediction seems to be true, as determined by biological evidence for some of the SCGs. Examples include DNA methyltransferase DNMT3A^39^; DNA repair enzyme POLQ^40^; mitotic marker and mitotic chromosome-coating surfactant MKI67^41^; a myeloproliferative disease suppressor DIDO1^42^; a mediator complex subunit MED1^43^; a tumor suppressor TP53^44^; nuclear lamin LMNA^45^; a transcriptional regulator ZBTB24^46^; a tumor suppressor APC^47^; a centrosome regulator NDRG1^48^; an E3 ubiquitin ligase PRKN^49^; and a DNA repair protein RAD52^50^.

The present study found SCGs with race-pronounced variants that need further validation. We will consider multitudes of factors to select gene(s) for further validation, such as (a) statistical significance, (b) being an SCG, (c) type of variant/mutation (variants occurring in open reading frame may be prioritized with an assumption of direct effects on the protein function, compared with likely indirect effects of intron variants), (d) amount and quality of existing work, and (e) available resources. However, given the current gap in knowledge, the application and the selection of an appropriate model system for validation studies are challenging and limited. In part, because race has been a sensitive issue in medical biology, it has not been considered as a major parameter for molecular studies. A basic validation study (e.g., in vitro gene/genome manipulation in cultured cells) may have to be designed to consider and incorporate race-pronounced differences. For example, which CRC cell lines are best suited to investigate the effects of race-pronounced DEGs or variant SCGs may need to be determined. Emerging molecular racial disparities in cancers indicate a need to refine study models to reflect the elements of ethnicity-driven differences.

Overall, the current report focuses on (a) the presence of race-pronounced variants in CRCs, which leads to proposing (b) a need for further functional validation and (c) inclusion of validated genes in the NGS diagnostic panel and the drug development pipeline as targets. This approach will help refine precision medicine through discovering race-pronounced variants (that would otherwise be diluted in White-dominant samples) and validating them as druggable target genes.

This DNA mutation analysis of white, AA, and AI CRCs found another layer of molecular racial disparities. We propose that upcoming studies on cancer disparities should incorporate this viewpoint of race-pronounced molecular disparities in cancer and in cancer-development processes, toward effective precision therapy and prevention.

We admit following limitations. For AI samples, our analysis was limited to CRCs and analysis on germline mutations was not included. As an AI ancestry reference sequence is not established, we used a white or Asian ancestry sequence as reference. The number of Oklahoma AI CRC samples was small, and sample procurement from AIs remains challenging for this and other projects that rely on tissue specimens. Race was based on self-identification by patients, which may not accurately reflect biological ancestry at the genomic level. Refinements in the ancestry-specific reference genome may advance the knowledge and the impact of biology in cancer development.

## Methods

### Samples

Samples were collected as previously described^8^. The study was conducted in accordance with the Declaration of Helsinki. We procured DNA and RNA samples, collected during surgery, from 20 white patients (CRC and normal/benign; *N* = 40) and 20 AA patients (*N* = 40) (total 80 RNA and 80 DNA samples), as well as sera from 12 white CRC patients and 8 AA patients (from Dr. U. Manne, UAB) (the protocol approved by the IRB of UAB; # IRB 020830005. An informed consent waiver was granted by the IRB for this purpose). Samples were de-identified, other than racial information, which was not shared with data analysts until analyses were completed. Measurements were taken from distinct samples. Except for samples with quality issues (e.g., DNA/RNA degradation), all samples were included and analyzed. Due to the de-identification, some co-variate analyses were not conducted. We also procured CRC tissue samples from prospectively consented patients (*n* = 7, white and *n* = 6, AI) (from Dr. K.T. Morris, OUHSC, Surgery Department), but they did not come with sera. With Trizol reagent (Thermo Fischer/Invitrogen) DNA and RNA were extracted from the CRC samples from Dr. Morris. We followed sample collection, procurement, and analysis protocols approved by UAB and OUHSC, as appropriate. Written informed consent from participants was under the OUHSC-approved IRB protocol #7565. The PI of the protocol was Dr. Morris. Race/ethnicity categorization (White, Black or African American, American Indian or Alaska Native, Asian, and Native Hawaiian or Other Pacific Islander) was provided by researchers in accordance with guidelines provided by the U.S. Office of Management and Budget (OMB). These data are based on self-identification of participants.

### Cancer whole exome sequencing

Tissue DNA was extracted with Trizol (ThermoFischer/Invitrogen) and stored at −80 ^o^C until use. DNA samples were submitted to the OUHSC Institutional Research Core Facility. Exome libraries were built using Agilent’s SureSelect XT HS2 Library and Target Enrichment Kit following Agilent’s established protocols. The library construction was accomplished with 4–200 ng of DNA. During library construction, each of the libraries was indexed in order to multiplex for sequencing. Samples were normalized and pooled onto a 150 paired-end run on Illumina’s NextSeq 2000.

### Read processing

Our exome sequencing pipeline is a combined suite of Illumina software and other “industry standard” software packages (i.e., Bowtie, SAMTools, and in-house custom scripts). Raw sequence data (FASTQ format) were preprocessed to produce BAM files. Sequence reads were mapped to the reference genome (hg19) using the Bowtie2 program^51^ and the sorting and indexing of the reads using SAMtools (v1.10)^52^. The aligned sequence data underwent a QC protocol before variant detection and annotation. The assessment was comprised of (a) total reads; (b) library complexity— the ratio of unique reads to total reads mapped to target; (c) capture efficiency—the ratio of reads mapped to human versus reads mapped to target; (d) coverage distribution—80% at 20X required for completion; (e) capture uniformity; (f) raw error rates; (g) the Transition/Transversion ratio (Ti/Tv); (h) distribution of known and novel variants relative to dbSNP—typically < 7% using dbSNP build 155 in samples of European ancestry^53^; (i) fingerprint concordance > 99%; (j) sample homozygosity and heterozygosity and (k) sample contamination validation as described previously^54,55^. Samples with an overall alignment rate below 70% were excluded from further analysis. After stringent sample-level quality control, we analyzed 55 of 61 samples of individuals with sequence data.

### Variant detection and annotation

We used human genome version GRCh38.p14 as reference. Variants were then called using VarScan (v2.3.9), which produced files on SNPs and indels separately. The annotation process was accomplished with SVS version 8.9.1 (Golden Helix, Bozeman, MT, USA) using the formatted output (VCF-variant call format). The variants derived from sequencing data were annotated using annotation servers such as 1KG Phase 3-Variant Frequencies 5a with Genotype counts, dbNSFP Functional Predictions 3.0, and RefSeq Genes 105.20190906 v3, NCBI using SVS. These servers return annotations, including dbSNP rsID, gene names, accession numbers, predicted functional effect (e.g., splice-site, nonsynonymous, missense), prediction if the mutation is damaging using (SIFT, PolyPhen2, MutationTaster Pred, FATHMM) predictions, ancestral allele, dbSNP allele frequencies, and known clinical associations. To note, AI reference genome remains incomplete, and we in part used Asian genome as reference. To analyze genome beyond accuracy of self-identification statement, improving infrastructure of molecular ethnicity study should be in the scope of future studies^56^.

To explore the role of the variants in the intronic or splice regions affecting gene regulation, we used various bioinformatics tools. The effect of these variants on transcription factor binding sites (TFBS) was analyzed using the atSNP search^57^. This search is a comprehensive web database that evaluates motif matches to the human genome with both reference and variant alleles and assesses the overall significance of the variant alterations on the motif matches. The miRNASNP-v3 tool was used to predict the effects of SNVs on pre-miRNA secondary structure changes, functional enrichment analysis of gained or lost targets by variations in miRNA, and miRNA target-binding alterations^58^.

### Statistical analysis

Association analyses were performed on 33 tumor and 22 normal samples. The variants were categorized based on their functional effect (UTRs, loss-of-function (LoF), missense, in-frame deletion/insertion, and introns). Statistical significance between the mutation or /minor allele frequencies (MAF) was calculated using the chi-square test. Variants showing statistically significant differences (*p* < =0.05) in the MAF were selected for further analysis. Distribution of the significant variants was analyzed across ethnicities to identify variants common or unique for a racial group. The association analysis was performed with SVS version 8.9.1 (Golden Helix, Bozeman, MT, USA).

### Re-assessments of transcriptomic alterations

To re-analyze RNAseq data of a previous study and to compare transcription of genes of interest that emerged from the present study, RNAseq data from the Gene Expression Omnibus (GEO) (NIH, US) GSE237684 were downloaded and re-analyzed as previously described^8^. Briefly, reads were normalized using DESeq. The normalized read counts were log-transformed and base-lined to the data set, resulting in normalized signal values. Gene expression of the normalized signal values between the control and experimental groups was determined. A moderated *t*-test, *p* < 0.05, was considered significant. A greater than 2-fold difference (log2 gene expression >1 or <−1) was considered differentially expressed. CMS was analyzed as described^13^. We used an R package CMScaller to conduct the CMS analysis by race groups. CMScaller (available at https://github.com/peterawe/CMScaller) provides CMS classification of colorectal cancer pre-clinical models based on a filtered set of cancer cell-intrinsic, subtype-enriched gene expression markers^13^. We input the RNAseq data (from previous study^8^; original data deposited to Gene Expression Omnibus (GEO) (NIH, US) as GSE237684) into the CMScaller, which returned the distribution of samples in the CMS groups.

### SCG identification

For each of the genes in the list (Supplementary Tables 1–3), we consulted cBioportal (www.cbioportal.com)^9–11^. The database consulting was performed during January 2024. For aggregated CRC, “Bowel cancer” datasets including 7661 samples/7435 patients from 19 studies were used. For the dataset with mRNA counts, we selected the “Sidra LUMC-AC-ICAM” study dataset with 384 CRC samples^59^. Based on the cBioportal algorism, CRCs with the gene of interest altered and unaltered were grouped, and the survival rate of patients, as well as various clinical attributes, were compared between gene-altered and gene-unaltered groups. The “Survival rate” included all types offered as an option—overall, disease-specific, disease-free, and progression-free. Any survival type with a *p*-value < 0.05 was identified as SCGs. Relevant clinical attributes (i.e., Race category; TMB/Tumor Mutational Burden (nonsynonymous); MSI/Microsatellite instability status; mICRo score; ICR HML/Immunologic Constant of Rejection genes high, medium, and low; CMS/Consensus Molecular Subtypes; Sex/gender; CIMP/CpG island methylator phenotype category) were selected and, if the *p*-value was <0.05, recorded, figures were generated with integrated algorithms and downloaded.

### Protein-protein interaction map

We used String ver12.0 (https://string-db.org/) to visualize protein-protein interactions of select genes of interest. Downloaded data are freely available under a ‘Creative Commons BY 4.0’ license. The default setting was used [i.e., medium confidence (0.400), no more than 10 interactions, FDR < 0.05, strength > 0.01, minimum content of network = 2, no clustering].

## Supplementary information

Supplementary Information Supplementary Fugure1 Tables 1 to 5

## Data Availability

The exome sequencing data generated in this study have been submitted to the Sequence Read Archive (SRA) (NIH, US) and will be publicly available upon publication of this manuscript (SRA accession number: PRJNA1166322).
