# Supplementary material for: Mutational disparities in colorectal cancers of White Americans, Alabama African Americans, And Oklahoma American Indians
Source: NPJ Precis Oncol. 2024 Dec 23;8:288. doi: 10.1038/s41698-024-00782-9 (PMC11666716; doi:10.1038/s41698-024-00782-9)
Supplement: Supplementary file 1 — Supplementary Information Supplementary Fugure1 Tables 1 to 5 [file 41698_2024_782_MOESM1_ESM.pdf]

(a)

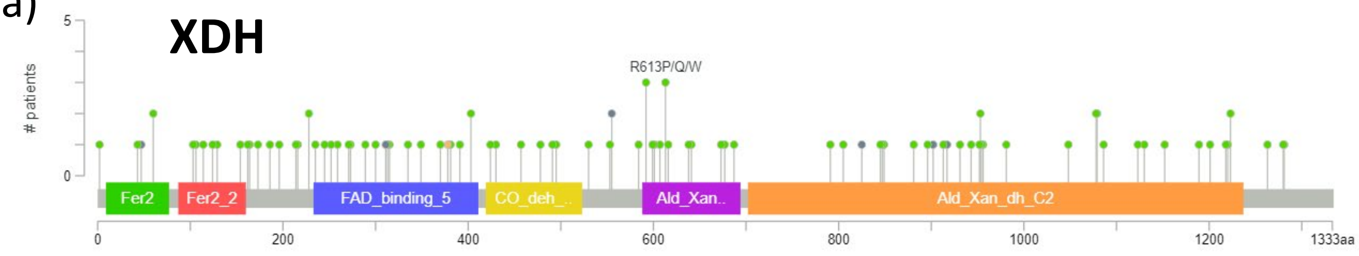

Color Codes

Mutation diagram circles are colored with respect to the corresponding mutation types. In case of different mutation types at a single position, color of the circle is determined with respect to the most frequent mutation type.

Mutation types and corresponding color codes are as follows:

- **Missense Mutations** (putative driver)
- **Missense Mutations** (unknown significance)
- **Truncating Mutations** (putative driver): Nonsense, Nonstop, Frameshift deletion, Frameshift insertion, Splice site
- **Truncating Mutations** (unknown significance): Nonsense, Nonstop, Frameshift deletion, Frameshift insertion, Splice site
- **Inframe Mutations** (putative driver): Inframe deletion, Inframe insertion
- **Inframe Mutations** (unknown significance): Inframe deletion, Inframe insertion
- **Splice Mutations** (putative driver)
- **Splice Mutations** (unknown significance)
- **Fusion Mutations**
- **Other Mutations** (putative driver): All other types of mutations
- **Other Mutations** (unknown significance): All other types of mutations

(b)

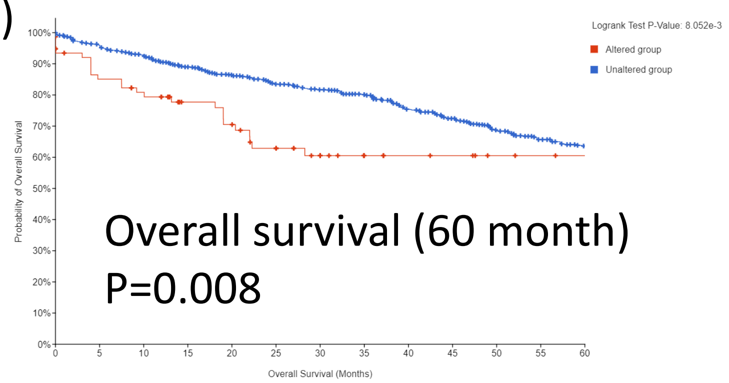

Number at risk (n)

Altered group	77	61	56	43	39	32	24	17	15	14	11	10	9
Unaltered group	2542	2125	1974	1653	1369	1174	987	770	590	498	386	306	261

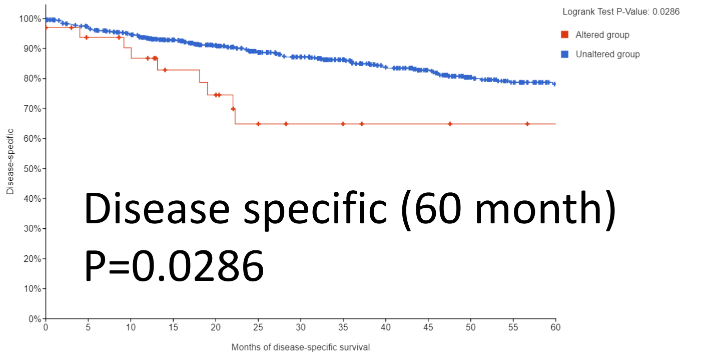

Number at risk (n)

Altered group	33	28	26	20	18	13	11	10	9	9	8	8	7
Unaltered group	796	716	672	587	504	445	390	337	280	249	201	173	146

Supplementary Figure 1

“XDH is a survival-critical gene whose alteration is associated with poorer survival.

(a) Mapping of 124 alterations of the XDH gene in CRC samples. Based on bowel cancer samples in cBioportal, 124 CRCs had altered XDH; 3472 CRCs did not. The alterations occurred throughout open reading frames of the XDH gene. Mutations in introns may be assessed through whole-genome exon sequencing and not through entries from mRNA sequencing.

(b) The overall survival rate was poorer for patients with XDH-altered CRCs (p=0.008, 60 months). Survival rates were calculated by a cBioportal algorithm. Analysis was performed on Sept 6, 2024, with 8611 samples from 24 studies.

(c) The disease-specific survival rate is poorer for patients with XDH-altered CRCs (p=0.0286, 60 months).”

Supplementary Table 1. Data for whites

Genes with race-pronounced variant allele frequencies in CRC samples of whites (sheet 1).

SCGs with race-pronounced variant allele frequencies in CRC samples of whites (sheet 2), including brief notes on gene function and PubMed publication numbers (as of 1/31-2/2, 2024).

Supplementary Table 2. Data for AAs

Genes with race-pronounced variant allele frequencies in CRC samples of AAs (sheet 1)

SCGs with race-pronounced variant allele frequencies in CRC samples of AAs (sheet 2), including brief notes on gene function and PubMed publication numbers (as of 1/31-2/2, 2024).

Supplementary Table 3. Data for AIs

Genes with race-pronounced variant allele frequencies in CRC samples of AIs (sheet 1).

SCGs with race-pronounced Variant Allele Frequencies in CRC samples of AIs (sheet 2) including brief notes on gene function and PubMed publication numbers (as of 1/31-2/2, 2024).

Predictor	Identifier	Gene Names	Sequence Ontology	Effect	MAF	Beta	SE	P-Value	survival influence (p<0.0	other notables p<0.05	full gene name	protein and function
22:18910285-Del	rs5844366	PRODH	intron_variant	Other	0.43	19.63	19372660.35	2.54E-04	no	MSI, TMB, race category	proline dehydrogenase 1	a mitochondrial protein that catalyzes the first step in proline degradation
13:47279376-Ins	rs11483731	LRCH1	intron_variant	Other	0.32	19.01	17935598.34	8.09E-04	no	mutation count, TMB, race categc	Leucine-Rich Repeats And Calponin	Acts as a negative regulator of GTPase CDC42 by sequestering CDC42-guanine exchange factor DOCK8
12:1902766-SNV	rs2058111	CACNA2D4	3_prime_UTR_variant	Other	0.32	18.84	16777216.00	1.12E-03	no	mutation count, TMB, race categc	Calcium Voltage-Gated Channel Au	a protein in the voltage-dependent calcium channel complex
2:219509618-SNV	rs2230115	ZNF142	missense_variant	Missense	0.44	1.45	0.59	3.18E-03	no	MSI, TMB, race category, sex	Zinc Finger Protein 142	May be involved in transcriptional regulation.
6:25652569-Del	rs3215471	SCGN	5_prime_UTR_variant	Other	0.40	-1.84	0.76	3.54E-03	prog free p=0.0343	MSI, TMB, race category	Secretagogin, EF-Hand Calcium Bin	a secreted calcium-binding protein, thought to be involved in KCL-stimulated calcium flux and cell proliferation
9:130457373-Ins					0.27	18.79	19372660.35	5.12E-03				
16:75301624-Ins		BCAR1	intron_variant	Other	0.30	19.56	27397079.00	5.47E-03	no	MSI, TMB, race category, CIMP hi	BCAR1 Scaffold Protein, Cas Family	Docking protein which plays a central coordinating role for tyrosine kinase-based signaling related to cell adhesion
19:51412775-Del		KLK4	intron_variant	Other	0.30	19.56	27397079.00	5.47E-03	no	mutation count, TMB, race categc	Kallikrein Related Peptidase 4	a serine protease
6:31829596-Ins		NEU1	intron_variant	Other	0.25	18.86	21221686.14	6.76E-03	no	MSI, TMB, mutation count	Neuraminidase 1	Catalyzes the removal of sialic acid (N-acetylneuraminic acid) moieties from glycoproteins and glycolipids
1:205312767-SNV	rs7526603	KLHDC8A	5_prime_UTR_variant	Other	0.25	18.66	19372660.35	9.11E-03	no (dis free p=0.0573)	MSI, TMB, race category, CIMP hi	Kelch Domain Containing 8A	a kelch domain-containing protein which is upregulated in cancer
16:71956594-Ins	rs3038732	IST1	intron_variant	Other	0.24	18.79	21221686.14	9.36E-03	no	MSI, TMB, race category	IST1 Factor Associated With ESCRT	ESCRT-III-like protein involved in cytokinesis, nuclear envelope reassembly and endosomal tubulation
1:235746949-Ins		GNG4	intron_variant	Other	0.22	18.95	23726566.41	9.62E-03	prog free p=0.0264	mutation count	G Protein Subunit Gamma 4	Involved in negative regulation of cell growth.
10:90534075-Ins		LIPN	intron_variant	Other	0.31	19.01	23726566.41	1.09E-02	prog free p=0.0234	Race category, MSI,	Lipase Family Member N	a lipase that is highly expressed in granular keratinocytes in the epidermis, and plays a role in the differentiation of keratinocytes
15:23258265-Del	rs199827219	GOLGA8IP	intron_variant	Other	0.27	-1.35	0.62	1.16E-02	no (Overall p=0.0736)		Golgin A8 Family Member I, Pseudo	Predicted to be involved in Golgi organization
3:48929310-Ins		SLC25A20	intron_variant	Other	0.20	19.63	33554432.00	1.29E-02	no	Race category	Solute Carrier Family 25 Member 2(	mediates the transport of acylcarnitines into mitochondrial matrix for their oxidation by the mitochondrial fatty acid-oxidation pathway
12:55354821-Del		TESPA1	intron_variant	Other	0.20	19.63	33554432.00	1.29E-02	no	Race category, TMB, MSI	Thymocyte Expressed, Positive Sele	Required for the development and maturation of T-cells,Plays a role in T-cell antigen receptor (TCR)-mediated activation of the ERK and NFAT signaling pathways
19:3543479-Del	rs34196068	C19orf71	intron_variant	Other	0.29	18.59	21221686.14	1.43E-02	no	Race category, MSI, TMB,	TEKTIP1/Tektin Bundle Interacting I	Located at the center of the tektin bundle where may function to recruit tektins or stabilize the bundle.
1:213069563-SNV	rs12567713	FLVCR1	3_prime_UTR_variant	Other	0.20	18.66	21221686.14	1.49E-02	Dis free p=0.0484	MSI	FLVCR Choline And Heme Transpor	a heme transporter that may play a critical role in erythropoiesis by protecting developing erythroid cells from heme toxicity
11:126147697-SNV	rs667627	FOXRED1	3_prime_UTR_variant	Other	0.20	18.66	21221686.14	1.49E-02	Prog free p=0.0428	Race category, TMB, MSI, ICR higl	FAD Dependent Oxidoreductase Dc	may function as a chaperone protein required for the function of mitochondrial complex I
1:146767320-SNV	rs6537	CHD1L	3_prime_UTR_variant	Other	0.40	19.01	23726566.41	1.58E-02	Dis free p=0.0476	TMB, MSI	Chromodomain Helicase DNA Bindi	a DNA helicase protein involved in DNA repair
1:204931531-Del		NFASC	intron_variant	Other	0.22	19.56	33554432.00	1.68E-02	Overall p=0.0274	MSI, Race category, TMB, CIMP,	Neurofascin	an L1 family immunoglobulin cell adhesion molecule, functions in neurite outgrowth
19:51465201-Ins	rs112376245	KLK6	intron_variant	Other	0.21	-1.49	0.69	1.77E-02	no	Race category, MSI, TMB,	Kallikrein Related Peptidase 6	a member of the kallikrein subfamily of the peptidase S1 family of serine proteases
7:107013040-Ins		COG5	frameshift_variant	LoF	0.20	18.79	23726566.41	1.80E-02	no	Race category, MSI, TMB,	Component Of Oligomeric Golgi Co	one of eight proteins (Cog1-8) which form a Golgi-localized complex (COG) required for normal Golgi morphology and function
17:7999589-SNV	rs3809882	ALOXE3	3_prime_UTR_variant	Other	0.48	1.04	0.48	1.89E-02	no	TMB, race category, MSI	Arachidonate Lipoxygenase 3	synthesizes a unique type of epoxy alcohol (8R-hydroxy-11R,12R-epoxyeicosa-5Z,9E,14Z-trienoic acid) from 12R-hydroperoxyeicosatetraenoic acid (12R-HPETE). This epoxy alcohol can activate the the nuclear receptor peroxisome proliferator-activated receptor alpha (PPARalpha),
5:140254821-Ins	HA2,PCDHA3,PCDHA4,PCDHA5,PCDHA6,PC		splice_region_variant	Other	0.30	19.12	27397079.00	1.92E-02	Prog free p=0.0046	MSI, TMB, CIMP	Protocadherin gene cluster	
3:183059200-Del		MCF2L2	intron_variant	Other	0.30	19.12	27397079.00	1.92E-02	no	Race category, TMB, CMS1 and 3	MCF.2 Cell Line Derived Transformi	Probably functions as a guanine nucleotide exchange factor.
7:103767477-Del		ORC5	intron_variant	Other	0.30	19.12	27397079.00	1.92E-02	no	MSI, Race category, TMB, CIMP,	Origin Recognition Complex Subuni	Component of the origin recognition complex (ORC) that binds origins of replication
6:170844209-Del		PSMB1	3_prime_UTR_variant	Other	0.30	19.12	27397079.00	1.92E-02	no		Proteasome 20S Subunit Beta 1	Non-catalytic component of the 20S core proteasome complex
18:59959003-Del		RELCH	intron_variant	Other	0.30	19.12	27397079.00	1.92E-02	no	TMB, race category, MSI, CMS1, I(	RAB11 Binding And LisH Domain, Cc	Regulates intracellular cholesterol distribution from recycling endosomes to the trans-Golgi network through interactions with RAB11 and OSBP
1:192544816-Del		RGS1	upstream_gene_variant	Unknown	0.30	19.12	27397079.00	1.92E-02	Dis free p=0.00919	Race category, mut count	Regulator Of G Protein Signaling 1	Regulates G protein-coupled receptor signaling cascades
16:57247659-Ins		RSPRY1	intron_variant	Other	0.30	19.12	27397079.00	1.92E-02	no (overall p=0.0536)	Race category, MSI, TMB,	Ring Finger And SPRY Domain Conta	a glycoprotein that contains a RING-type zinc finger domain and an SPRY domain of unknown function
4:85748220-Del		WDFY3	intron_variant	Other	0.30	19.12	27397079.00	1.92E-02	no	MSI, TMB, race category, CIMP, C	WD Repeat And FYVE Domain Cont	Required for selective macroautophagy (aggrephagy).
2:56606764-Ins		CCDC85A	intron_variant	Other	0.20	19.01	27397079.00	1.96E-02	no	Race category, MSI, TMB, sex	Coiled-Coil Domain Containing 85A	May play a role in cell-cell adhesion and epithelium development through its interaction with proteins of the beta-catenin family.
18:67727624-Del		RTTN	intron_variant	Other	0.20	19.01	27397079.00	1.96E-02	no	TMB,MSI, CMS1 and 3, ICR high, n	Rotatin	Involved in the genetic cascade that governs left-right specification. Plays a role in the maintenance of a normal ciliary structure.
6:166743628-Ins		SFT2D1	intron_variant	Other	0.20	19.01	27397079.00	1.96E-02	Overall p=0.0103	Race category, MSI	SFT2 Domain Containing 1	May be involved in fusion of retrograde transport vesicles derived from an endocytic compartment with the Golgi complex.
5:34888746-Del		TTC23L	intron_variant	Other	0.20	18.79	19372660.35	1.96E-02	no	Race category, TMB, MSI,	Tetratricopeptide Repeat Domain 2	Predicted to be located in cytoplasm; microtubule cytoskeleton; and midbody.
10:102673063-SNV	rs3802725	SLF2	3_prime_UTR_variant	Other	0.36	1.17	0.59	1.97E-02	no	TMB, MSI, race category, micro sc	SMC5-SMC6 Complex Localization	Plays a role in the DNA damage response (DDR) pathway by regulating postreplication repair of UV-damaged DNA and genomic stability maintenance
7:150813978-Ins	rs76473497	AGAP3	intron_variant	Other	0.23	18.60	21221686.14	2.09E-02	no	MSI, Rrace category, TMB, CIMP,	ArfGAP With GTPase Domain, Anky	an essential component of the N-methyl-D-aspartate (NMDA) receptor signaling complex which mediates long-term potentiation in synapses
2:219146868-SNV	rs2292554	PNKD,TMBIM1	5_prime_UTR_variant	Other	0.35	1.18	0.60	2.17E-02				Variation at 2q35 (PNKD and TMBIM1) influences colorectal cancer risk and identifies a pleiotropic effect with inflammatory bowel disease
3:171426433-Ins		PLD1	intron_variant	Other	0.18	18.95	27397079.00	2.18E-02	Dis free p=0.0305	TMB, MSI,	Phospholipase D1	Implicated as a critical step in numerous cellular pathways, including signal transduction, membrane trafficking, and the regulation of mitosis.
7:92977998-Ins		VPS50	intron_variant	Other	0.18	18.95	27397079.00	2.18E-02	no	TMB, race category	VPS50 Subunit Of EARP/GARPII Cor	Enables SNARE binding activity.
2:25497685-Ins	rs367544298	DNMT3A	intron_variant	Other	0.35	-1.30	0.64	2.25E-02	Overall p=0.0217	MSI, race category, TMB, sex, ICR	DNA Methyltransferase 3 Alpha	a DNA methyltransferase that is thought to function in de novo methylation, rather than maintenance methylation.
3:121507372-Del	rs141690316	IQCB1	intron_variant	Other	0.17	18.55	16777216.00	2.25E-02	no (Dis specific p=0.0710)	Race category, TMB	IQ Motif Containing B1	Involved in ciliogenesis.
18:59174447-Ins		CDH20	intron_variant	Other	0.25	19.46	33554432.00	2.29E-02	no (prog free p=0.0978)	TMB, MSI, race category,	Cadherin 20	a calcium dependent cell-cell adhesion glycoprotein
1:44587669-Del		KLF17	intron_variant	Other	0.25	19.46	33554432.00	2.29E-02	no	Race category, TMB	KLF Transcription Factor 17	Transcription repressor that binds to the promoter of target genes and prevents their expression.
2:192246057-Ins		MYO1B	intron_variant	Other	0.25	19.46	33554432.00	2.29E-02	Overall p=0.00253	MSI, race category, TMB, CMS1	Myosin IB	Involved in actin filament organization and post-Golgi vesicle-mediated transport.
9:116080657-Ins		WDR31	intron_variant	Other	0.25	19.46	33554432.00	2.29E-02	no	Race category, TMB, MSI	WD Repeat Domain 31	may facilitate formation of heterotrimeric or multiprotein complexes
2:31557091-Ins		XDH	downstream_gene_variant	Unknown	0.25	19.46	33554432.00	2.29E-02	Dis specific p=0.0286	MSI, Race category, TMB, CIMP,	Xanthine Dehydrogenase	Key enzyme in purine degradation. contributes to the generation of reactive oxygen species.
15:20862860-Ins					0.25	19.46	33554432.00	2.29E-02				
10:135339843-Del					0.25	19.46	33554432.00	2.29E-02				
11:60774304-Del		CD6	intron_variant	Other	0.25	19.01	27397079.00	2.29E-02	no (dis specific p=0.0748)	MSI, TMB, race category,	CD6 Molecule	Cell adhesion molecule that mediates cell-cell contacts and regulates T-cell responses via its interaction with ALCAM/CD166, important for continuation of T cell activation
11:117391736-Del		DSCAML1	intron_variant	Other	0.25	19.01	27397079.00	2.29E-02	Overall p=0.00065	MSI, TMB, CMS, CIMP,	DS Cell Adhesion Molecule Like 1	Cell adhesion molecule that plays a role in neuronal self-avoidance
2:56102253-Del		EFEMP1	intron_variant	Other	0.25	19.01	27397079.00	2.29E-02	no	Race category, TMB	EGF Containing Fibulin Extracellular	Binds EGFR, the EGF receptor, inducing EGFR autophosphorylation and the activation of downstream signaling pathways. May play a role in cell adhesion and migration.
8:95448623-Ins		FSBP,RAD54B	intron_variant	Other	0.25	19.01	27397079.00	2.29E-02				
2:54849365-Ins-2		SPTBN1	intron_variant	Other	0.25	19.01	27397079.00	2.29E-02	no	MSI, TMB, race category, CIMP, C	Spectrin Beta, Non-Erythrocytic 1	an actin crosslinking and molecular scaffold protein that links the plasma membrane to the actin cytoskeleton, and functions in the determination of cell shape, arrangement of transmembrane proteins, and organization of organelles.
12:57642314-Ins		STAC3	intron_variant	Other	0.25	19.01	27397079.00	2.29E-02	no (overall p=0.0686)	Race category, TMB	SH3 And Cysteine Rich Domain 3	Required for normal excitation-contraction coupling in skeletal muscle
19:53627440-Ins		ZNF415	intron_variant	Other	0.25	19.01	27397079.00	2.29E-02	no	Race category, TMB	Zinc Finger Protein 415	Involved in transcriptional regulation
6:107097124-Del	rs3040665	QRSL1	intron_variant	Other	0.25	18.59	21221686.14	2.30E-02	no (dis free p=0.0849)	TMB, MSI	Glutaminyl-TRNA Amidotransferase	Involved in glutaminyl-tRNAGln biosynthesis via transamidation and mitochondrial translation.
4:134111466-Del		PCDH10	3_prime_UTR_variant	Other	0.16	18.92	27397079.00	2.37E-02	Overall p=0.0406	MSI, race category, TMB, CIMP, C	Protocadherin 10	a cadherin-related neuronal receptor thought to function in the establishment of specific cell-cell connections in the brain.
2:20179533-Del	rs767542195	WDR35	intron_variant	Other	0.24	18.49	16777216.00	2.41E-02	Dis free p=0.0467	Race category, TMB, MSI,	WD Repeat Domain 35	involved in ciliogenesis and ciliary protein trafficking
13:39453239-Del		FREM2	intron_variant	Other	0.21	18.94	27397079.00	2.55E-02	no	MSI, TMB, CMS1	FRAS1 Related Extracellular Matrix	localizes to the basement membrane, important for the integrity of skin and renal epithelia
17:45376531-Ins		ITGB3	intron_variant	Other	0.21	18.94	27397079.00	2.55E-02	no	Race category, TMB, MSI, CIMP,	Integrin Subunit Beta 3	receptor for cytotactin, fibronectin, laminin, matrix metalloproteinase-2, osteopontin, osteomodulin, prothrombin, thrombospondin, vitronectin and von Willebrand factor.
3:121258271-Ins		POLQ	intron_variant	Other	0.21	18.94	27397079.00	2.55E-02	Overall p=0.0491	MSI, race category, TMB, CMS1 a	DNA Polymerase Theta	DNA polymerase that promotes microhomology-mediated end-joining (MMEJ), an alternative non-homologous end-joining (NHEJ) machinery triggered in response to double-strand breaks in DNA
7:122774381-Ins-2		SLC13A1	intron_variant	Other	0.21	18.94	27397079.00	2.55E-02	no	Race category, TMB	Solute Carrier Family 13 Member 1	Sodium:sulfate symporter that mediates sulfate reabsorption in the kidney and small intestine
1:94487286-Del	rs55860151	ABCA4	intron_variant	Other	0.17	18.66	23726566.41	2.72E-02	no	MSI, race category, TMB, CIMP	ATP Binding Cassette Subfamily A M	a retina-specific ABC transporter with N-retinylidene-PE as a substrate.
10:129904474-SNV	rs61738284	MKI67	missense_variant	Missense	0.17	18.66	23726566.41	2.72E-02	Dis free p=0.0453	MSI, race category, TMB, CIMP, I(	Marker Of Proliferation Ki-67	has a high net electrical charge and acts as a surfactant, dispersing chromosomes and enabling independent chromosome motility
12:69995273-SNV	rs7200	CCT2	3_prime_UTR_variant	Other	0.19	18.89	27397079.00	2.75E-02	no	Race category, TMB, MSI	Chaperonin Containing TCP1 Subun	Component of the chaperonin-containing T-complex (TRiC), a molecular chaperone complex that assists the folding of proteins upon ATP hydrolysis
16:86575216-Ins		MTHFSD	intron_variant	Other	0.19	18.89	27397079.00	2.75E-02	Prog free p=0.0493	MSI, TMB	Methenyltetrahydrofolate Syntheta	Enables RNA binding activity
1:103348627-Del		COL11A1	intron_variant	Other	0.18	18.66	23726566.41	2.82E-02	Overall p=0.0322	MSI, race category, TMB, CMS1, C	Collagen Type XI Alpha 1 Chain	May play an important role in fibrillogenesis by controlling lateral growth of collagen II fibrils.
2:186604030-Ins-2		FSIP2,FSIP2-AS2	intron_variant	Other	0.20	18.66	23726566.41	2.95E-02	no	MSI, race category, TMB, CMS, ICI	Fibrous Sheath Interacting Protein :	a protein associated with the sperm fibrous sheath
17:6554921-SNV	rs74504320	MED31	5_prime_UTR_variant	Other	0.20	18.66	23726566.41	2.95E-02	no (overall p=0.0874)	Race category	Mediator Complex Subunit 31	Component of the Mediator complex, a coactivator involved in the regulated transcription of nearly all RNA polymerase II-dependent genes.
5:149385920-Ins		HMGXB3	intron_variant	Other	0.33	19.01	27397079.00	2.97E-02	no (dis specific p=0.0777)	Race category, TMB, MSI, CMS1	HMG-Box Containing 3	one of the non-canonical high mobility group (HMG) genes
19:9037996-Ins		MUC16	intron_variant	Other	0.33	18.76	19372660.35	2.97E-02	Dis free p=0.0295	MSI, race category, TMB, CMS1 a	Mucin 16, Cell Surface Associated	thought to play a role in forming a barrier, protecting epithelial cells from pathogens. Products of this gene have been used as a marker for different cancers, with higher expression levels associated with poorer outcomes.
3:155218297-Ins		PLCH1	intron_variant	Other	0.33	19.01	27397079.00	2.97E-02	no	MSI, race category, TMB, CMS1	Phospholipase C Eta 1	an enzyme to generate second messengers inositol 1,4,5-trisphosphate (IP3) and diacylglycerol (DAG)
1:94998138-Del		F3	intron_variant	Other	0.33	19.01	27397079.00	2.97E-02	no	Race category, mut count	Coagulation Factor III, Tissue Facto	Initiates blood coagulation by forming a complex with circulating factor VII or VIIa.
4:48549561-Ins		FRYL	intron_variant	Other	0.33	19.01	27397079.00	2.97E-02	Overall p=0.045	MSI, race category, TMB, CMS1, C	FRY Like Transcription Coactivator	Plays a key role in maintaining the integrity of polarized cell extensions during morphogenesis, regulates the actin cytoskeleton
18:44683394-Ins		IER3IP1	intron_variant	Other	0.33	19.01	27397079.00	2.97E-02	no	Race category	Immediate Early Response 3 Intera	localized to the endoplasmic reticulum (ER) and may play a role in the ER stress response by mediating cell differentiation and apoptosis.
16:68380892-Ins		PRMT7	intron_variant	Other	0.33	19.01	27397079.00	2.97E-02	no	MSI, race category, TMB, CIMP,	Protein Arginine Methyltransferase	an enzyme transfers single methyl groups to arginine residues to generate monomethylarginines on histone proteins as well as other protein substrates
3:48420835-Del		FBXW12	intron_variant	Other	0.15	18.84	27397079.00	3.03E-02	Prog free p=0.0260	Race category, TMB	F-Box And WD Repeat Domain Con	Substrate-recognition component of the SCF (SKP1-CUL1-F-box protein)-type E3 ubiquitin ligase complex
12:53679654-Ins		ESPL1	intron_variant	Other	0.22	18.66	23726566.41	3.11E-02	no	MSI, race category, TMB, CMS, CII	Extra Spindle Pole Bodies Like 1, Se	Caspase-like protease, which plays a central role in the chromosome segregation by cleaving the SCC1/RAD21 subunit of the cohesin complex at the onset of anaphase.
19:33467676-SNV	rs55853151	FAAP24	3_prime_UTR_variant	Other	0.22	18.66	23726566.41	3.11E-02	no	MSI	FA Core Complex Associated Protei	a component of the Fanconi anemia (FA) core complex, which plays a crucial role in DNA damage response
12:96063948-Del	rs3833792	NTN4,PGAM1P5	intron_variant	Other	0.33	1.10	0.59	3.12E-02				
3:130311398-SNV	rs73868925	COL6A6	missense_variant	Missense	0.16	18.61	23726566.41	3.22E-02	no	MSI, TMB, CIMP, race category	Collagen Type VI Alpha 6 Chain	Collagen VI acts as a cell-binding protein.
17:76547634-SNV	rs16971526	DNAH17	missense_variant	Missense	0.16	18.61	23726566.41	3.22E-02	Overall p=0.00475	MSI, race category, TMB, CIMP, I(	Dynein Axonemal Heavy Chain 17	Plays a major role in sperm motility, implicated in sperm flagellar assembly and beating
5:169810796-SNV	rs11739136	KCNIP1,KCNMB1	missense_variant	Missense	0.16	18.61	23726566.41	3.22E-02				
2:158406592-Ins-2		ACVR1C	intron_variant	Other	0.27	18.92	27397079.00	3.25E-02	no	TMB, MSI	Activin A Receptor Type 1C	Plays a role in cell differentiation, growth arrest and apoptosis.
4:100232929-Ins		ADH1B	intron_variant	Other	0.27	18.92	27397079.00	3.25E-02	no	TMB, MSI,	Alcohol Dehydrogenase 1B (Class I)	plays a major role in ethanol catabolism
3:112324705-Del		CCDC80	intron_variant	Other	0.27	18.92	27397079.00	3.25E-02	no	MSI, race category, TMB, CIMP	Coiled-Coil Domain Containing 80	Promotes cell adhesion and matrix assembly.
15:76032249-Ins-2		DNM1P35	splice_region_variant	Other	0.27	18.92	27397079.00	3.25E-02	no		Dynamin 1 Pseudogene 35	a Pseudogene.
2:234054660-Del		INPP5D	intron_variant	Other	0.27	18.92	27397079.00	3.25E-02	Dis free p=0.00259	Race category, TMB, MSI,	Inositol Polyphosphate-5-Phosphat	functions as a negative regulator of myeloid cell proliferation and survival
5:133887961-Del		JADE2	intron_variant	Other	0.27	18.92	27397079.00	3.25E-02	no (dis free p=0.0559)	MSI, TMB, race category,	Jade Family PHD Finger 2	Scaffold subunit of some HBO1 complexes, which have a histone H4 acetyltransferase activity, a E3 ubiquitin-protein ligase targeting protein histone demethylase KDM1A and self
2:75881800-Ins-2		MRPL19	intron_variant	Other	0.27	18.92	27397079.00	3.25E-02	no	Race category, TMB, MSI	Mitochondrial Ribosomal Protein L	a 39S large subunit protein of mitochondrial ribosome
10:69918731-Ins		MYPN	intron_variant	Other	0.27	18.92	27397079.00	3.25E-02	no (dis free p=0.0628)	Race category, TMB, MSI	Myopalladin	Component of the sarcomere that tethers together nebulin (skeletal muscle) and nebulette (cardiac muscle) to alpha-actinin, at the Z lines.
12:81674982-Ins		PPFIA2,PPFIA2-AS1	intron_variant	Other	0.27	18.92	27397079.00	3.25E-02				
4:26361434-Ins-2		RBPJ	intron_variant	Other	0.27	18.92	27397079.00	3.25E-02	no	Race category, TMB,	Recombination Signal Binding Prote	a transcriptional regulator important in the Notch signaling pathway.
15:45445690-Del		DUOX1	intron_variant	Other	0.44	1.18	0.62	3.26E-02	Dis free p=0.00272	MSI, TMB, race cetegory, CMS	Dual Oxidase 1	generates hydrogen peroxide and thereby plays a role in the activity of thyroid peroxidase, lactoperoxidase, and in lactoperoxidase-mediated antimicrobial defense at mucosal surfaces.
1:183083528-Ins		LAMC1	intron_variant	Other	0.25	18.66	23726566.41	3.32E-02	no	Race category, TMB, MSI,	Laminin Subunit Gamma 1	thought to mediate the attachment, migration and organization of cells into tissues during embryonic development by interacting with other extracellular matrix components
15:40565970-Ins		BUB1B-PAK6,PAK6	intron_variant	Other	0.23	18.86	27397079.00	3.42E-02				
4:140275066-Ins		NAA15	intron_variant	Other	0.23	18.86	27397079.00	3.42E-02	no	Race category, TMB, MSI,	N-Alpha-Acetyltransferase 15, NatA	Auxillary subunit of N-terminal acetyltransferase complexes which display alpha (N-terminal) acetyltransferase (NAT) activity, The NAT activity may be important for vascular, hematopoietic and neuronal growth and development
1:117484227-Ins		PTGFRN	intron_variant	Other	0.23	18.86	27397079.00	3.42E-02	no	MSI, race category, TMB, CMS1 a	Prostaglandin F2 Receptor Inhibitor	Inhibits the binding of prostaglandin F2-alpha (PGF2-alpha) to its specific FP receptor, by decreasing the receptor number rather than the affinity constant.
9:114147817-Del		ECPAS	intron_variant	Other	0.15	19.15	33554432.00	3.53E-02	no	MSI, race category, TMB,	Ecm29 Proteasome Adaptor And Sc	Adapter/scaffolding protein that binds to the 26S proteasome, motor proteins and other compartment specific proteins. May play a role in ERAD and other enhanced proteolysis
19:17450545-Del-2		GTPBP3	intron_variant	Other	0.15	19.15	33554432.00	3.53E-02	no (dis free p=0.0597)	Race category, TMB, MSI,	GTP Binding Protein 3, Mitochondri	localized to the mitochondria and may play a role in mitochondrial tRNA modification.
8:141756992-Del-2	rs369382658	PTK2	intron_variant	Other	0.15	18.90	23726566.41	3.53E-02	no (overall p=0.0640)	TMB, MSI, race category	Protein Tyrosine Kinase 2	Non-receptor protein-tyrosine kinase that plays an essential role in regulating cell migration, adhesion, spreading, reorganization of the actin cytoskeleton, formation and disassembly of focal adhesions and cell protrusions, cell cycle progression, cell proliferation and apoptosis.
16:71571834-Del		CHST4	3_prime_UTR_variant	Other	0.15	19.15	33554432.00	3.53E-02	no (prog free p=0.0743)	Race category, TMB, CIMP	Carbohydrate Sulfotransferase 4	localized to the Golgi and is involved in the modification of glycan structures on ligands of the lymphocyte homing receptor L-selectin.
16:71571835-Ins		CHST4	3_prime_UTR_variant	Other	0.15	19.15	33554432.00	3.53E-02	see line 105			
5:121776565-Ins		MGC32805,SNCAIP	intron_variant	Other	0.15	19.15	33554432.00	3.53E-02				
12:9220663-Ins		A2M	intron_variant	Other	0.20	18.58	19372660.35	3.54E-02	no (prog free p=0.0525)	Race category, TMB, MSI, CIMP,	Alpha-2-Macroglobulin	a protease inhibitor and cytokine transporter.
18:12093931-Ins		ANKRD62	5_prime_UTR_variant	Other	0.20	18.83	27397079.00	3.54E-02	no	Race category, MSI, TMB	Ankyrin Repeat Domain 62	function unknown
4:68357747-Ins		CENPC	intron_variant	Other	0.20	18.58	19372660.35	3.54E-02	no	MSI, race category, TMB, CIMP,	Centromere Protein C	a centromere autoantigen and a component of the inner kinetochore plate
16:81705792-Del		CMIP	intron_variant	Other	0.20	18.65	19372660.35	3.54E-02	no	TMB, race category, MSI, CIMP,	C-Maf Inducing Protein	a c-Maf inducing protein that plays a role in T-cell signaling pathway
22:31980957-Ins		SFI1	intron_variant	Other	0.20	18.58	19372660.35	3.54E-02	no	TMB, MSI, race category, CMS1,	SFI1 Centrin Binding Protein	Plays a role in the dynamic structure of centrosome-associated contractile fibers via its interaction with CETN2.
6:152737500-Ins		SYNE1	intron_variant	Other	0.20	18.58	19372660.35	3.54E-02	no	MSI, race category, TMB, CIMP, C	Spectrin Repeat Containing Nuclear	Multi-isomeric modular protein which forms a linking network between organelles and the actin cytoskeleton to maintain the subcellular spatial organization. As a component of the LINC (LInker of Nucleoskeleton and Cytoskeleton) complex involved in the connection between the nuclear lamina
4:69188417-Ins		YTHDC1	intron_variant	Other	0.20	18.58	19372660.35	3.54E-02	no (dis free p=0.0743)	Race category, TMB, MSI,	YTH N6-Methyladenosine RNA Binc	Regulator of alternative splicing
3:130687973-Ins		ATP2C1	intron_variant	Other	0.18	18.80	27397079.00	3.62E-02	no	Race category, TMB, MSI, CIMP,	ATPase Secretory Pathway Ca2+ Tr	ATP-driven pump that supplies the Golgi apparatus with Ca(2+) and Mn(2+) ions, both essential cofactors for processing and trafficking of newly synthesized proteins in the secretory pathway
2:211521227-Del		CPS1	intron_variant	Other	0.18	18.80	27397079.00	3.62E-02	Overall p=0.0242	Race category, TMB, MSI,	Carbamoyl-Phosphate Synthase 1	The mitochondrial enzyme encoded by this gene catalyzes synthesis of carbamoyl phosphate from ammonia and bicarbonate. This reaction is the first committed step of the urea cycle,
5:147513280-Del		SPINK5	intron_variant	Other	0.18	18.80	27397079.00	3.62E-02	no	MSI, race category, TMB, CIMP,	Serine Peptidase Inhibitor Kazal Typ	Serine protease inhibitor, probably important for the anti-inflammatory and/or antimicrobial protection of mucous epithelia.
17:40647286-Ins		ATP6V0A1	intron_variant	Other	0.18	18.80	27397079.00	3.62E-02	no (dis free p=0.0975)	Race category, MSI, TMB, CMS1 a	ATPase H+ Transporting V0 Subunit	a component of vacuolar ATPase (V-ATPase), a multisubunit enzyme that mediates acidification of eukaryotic intracellular organelles.
2:216295342-Ins		FN1	intron_variant	Other	0.18	18.80	27397079.00	3.62E-02	no	MSI, race category, TMB, CIMP,	Fibronectin 1	involved in cell adhesion and migration processes
12:101012177-Ins		GAS2L3	intron_variant	Other	0.18	18.80	27397079.00	3.62E-02	no	Race category, MSI, TMB	Growth Arrest Specific 2 Like 3	Cytoskeletal linker protein. May promote and stabilize the formation of the actin and microtubule network.
15:40949712-Del		KNL1	intron_variant	Other	0.18	18.80	27397079.00	3.62E-02	no	TMB, race category, MSI, CMS,	Kinetochore Scaffold 1	a component of the multiprotein assembly that is required for creation of kinetochore-microtubule attachments and chromosome segregation
15:41028609-Ins		RMDN3	3_prime_UTR_variant	Other	0.18	18.80	27397079.00	3.62E-02	no (overall p=0.0617)	TMB, MSI, ICR high, CMS,	Regulator Of Microtubule Dynamic	Involved in cellular calcium homeostasis regulation.
14:21990772-Ins		SALL2	3_prime_UTR_variant	Other	0.18	18.80	27397079.00	3.62E-02	Dis free p=0.0452	MSI, race category, TMB, CIMP	Spalt Like Transcription Factor 2	Probable transcription factor that plays a role in eye development before, during, and after optic fissure closure.
20:61524385-Del	rs11362069	DIDO1	intron_variant	Other	0.29	18.66	23726566.41	3.62E-02	Overall p=0.003238	MSI, TMB, CIMP,	Death Inducer-Obliterator 1	Tumor suppressor. Required for early embryonic stem cell development.
12:120221620-Del		CIT	intron_variant	Other	0.16	18.79	27397079.00	3.69E-02	no	MSI, race category, TMB, CIMP, C	Citron Rho-Interacting Serine/Thre	a serine/threonine-protein kinase that functions in cell division and efficient cytokinesis.
5:138171074-Ins-2		CTNNA1	intron_variant	Other	0.16	18.79	27397079.00	3.69E-02	no	MSI, race category, TMB, CIMP	Catenin Alpha 1	Associates with the cytoplasmic domain of a variety of cadherins.
2:26477129-Ins		HADHB	inframe_insertion	Missense	0.16	18.79	27397079.00	3.69E-02	no	Race category, TMB, MSI	Hydroxyacyl-CoA Dehydrogenase T	the beta subunit of the mitochondrial trifunctional protein, which catalyzes the last three steps of mitochondrial beta-oxidation of long chain fatty acids
12:27180189-Ins		MED21	intron_variant	Other	0.16	18.79	27397079.00	3.69E-02	no	mut count	Mediator Complex Subunit 21	Component of the Mediator complex, a coactivator involved in the regulated transcription of nearly all RNA polymerase II-dependent genes.
15:48427021-Ins		MYEF2,SLC24A5	3_prime_UTR_variant	Other	0.16	18.79	27397079.00	3.69E-02				
5:41805572-Ins		OXCT1	intron_variant	Other	0.16	18.79	27397079.00	3.69E-02	no	TMB, MSI	3-Oxoacid CoA-Transferase 1	Key enzyme for ketone body catabolism. Catalyzes the first, rate-limiting step of ketone body utilization in extrahepatic tissues,
19:36585274-Ins		WDR62	intron_variant	Other	0.16	18.79	27397079.00	3.69E-02	Dis free p=0.0300	MSI, race category, TMB, CIMP,	WD Repeat Domain 62	Required for cerebral cortical development. Plays a role in neuronal proliferation and migration
1:17387860-Ins					0.16	18.79	27397079.00	3.69E-02				
21:45500092-Ins		TRAPPC10	intron_variant	Other	0.14	18.77	27397079.00	3.74E-02	no	Race category, TMB, MSI, CIMP, C	Trafficking Protein Particle Comple	part of the multisubunit transport protein particle (TRAPP) complex and may be involved in vesicular transport from the endoplasmic reticulum to the Golgi.
15:73852260-Ins		REC114	3_prime_UTR_variant	Other	0.14	18.77	27397079.00	3.74E-02	no	Race category, TMB,	REC114 Meiotic Recombination Pro	Required for DNA double-strand breaks (DSBs) formation in unsynapsed regions during meiotic recombination.
14:95081463-Del		SERPINA3	intron_variant	Other	0.14	18.77	27397079.00	3.74E-02	no	Race category, TMB, MSI,	Serpin Family A Member 3	a member of the serpin family of proteins, a group of proteins that inhibit serine proteases.
15:76577888-Ins		ETFA	intron_variant	Other	0.21	18.59	23726566.41	3.86E-02	no	Race category, TMB	Electron Transfer Flavoprotein Subu	participates in catalyzing the initial step of the mitochondrial fatty acid beta-oxidation
1:92944337-Ins		GFI1	intron_variant	Other	0.33	1.15	0.63	3.88E-02	no	Race category, TMB, MSI,	Growth Factor Independent 1 Trans	a nuclear zinc finger protein that functions as a transcriptional repressor. This protein plays a role in diverse developmental contexts, including hematopoiesis and oncogenesis.
5:140048707-SNV	rs35983033	WDR55	missense_variant	Missense	0.17	18.56	23726566.41	3.97E-02	no	Race category, TMB, MSI, CMS, IC	WD Repeat Domain 55	Nucleolar protein that acts as a modulator of rRNA synthesis.
7:99013991-Del		BUD31	intron_variant	Other	0.33	18.66	23726566.41	4.08E-02	no	mut count	BUD31 Homolog	Involved in mRNA splicing, via spliceosome and positive regulation of androgen receptor activity.
6:6318922-Ins	rs398000295	F13A1	splice_region_variant	Other	0.23	-1.13	0.62	4.12E-02	Dis free p=0.0281	Race category, TMB, MSI,	Coagulation Factor XIII A Chain	encodes the coagulation factor XIII A subunit
15:89753220-SNV	rs2710	RLBP1	3_prime_UTR_variant	Other	0.32	1.03	0.59	4.25E-02	no	Race category, TMB	Retinaldehyde Binding Protein 1	Soluble retinoid carrier essential the proper function of both rod and cone photoreceptors.
11:61165732-Ins	rs11382548	TMEM216	frameshift_variant	LoF	0.32	1.03	0.59	4.25E-02	no	Race category, MSI, TMB	Transmembrane Protein 216	Part of the tectonic-like complex which is required for tissue-specific ciliogenesis and may regulate ciliary membrane composition.
16:28840590-Ins		ATXN2L	intron_variant	Other	0.17	19.08	33554432.00	4.33E-02	no	MSI, race category, TMB, CIMP,	Ataxin 2 Like	Involved in the regulation of stress granule and P-body formation.
12:88477495-Ins		CEP290	intron_variant	Other	0.17	19.08	33554432.00	4.33E-02	no	MSI, race category, TMB, CMS, ICI	Centrosomal Protein 290	Involved in early and late steps in cilia formation.
3:71050020-Ins-3		FOXP1	intron_variant	Other	0.17	19.08	33554432.00	4.33E-02	no	Race category, TMB, MSI, sex,	Forkhead Box P1	may act as a tumor suppressor as it is lost in several tumor types and maps to a chromosomal region (3p14.1) reported to contain a tumor suppressor gene(s)
13:32977677-Ins		N4BP2L1	intron_variant	Other	0.17	19.08	33554432.00	4.33E-02	no (dis free p=0.0511)	Race category, MSI, ICR high, micr	NEDD4 Binding Protein 2 Like 1	An important paralog of this gene is N4BP2 that is a kinase involved in DNA repair
6:16306507-Ins		ATXN1	3_prime_UTR_variant	Other	0.14	18.75	27397079.00	4.47E-02	no (overall p=0.0798)	MSI, race category, TMB, CMS, CII	Ataxin 1	Chromatin-binding factor that repress Notch signaling in the absence of Notch intracellular domain by acting as a CBF1 corepressor.
7:16130380-Ins		CRPPA	3_prime_UTR_variant	Other	0.14	18.75	27397079.00	4.47E-02	no	Race category, TMB,	CDP-L-Ribitol Pyrophosphorylase A	Cytidylyltransferase required for protein O-linked mannosylation
6:73820971-Ins		KCNQ5	intron_variant	Other	0.15	18.72	27397079.00	4.50E-02	no	Race category, TMB, MSI,	Potassium Voltage-Gated Channel S	form a potassium channel which contributes to M-type current, a slowly activating and deactivating potassium conductance which plays a critical role in determining the subthreshold electrical excitability of neurons.
16:47697715-Ins		PHKB	intron_variant	Other	0.15	18.72	27397079.00	4.50E-02	no (overall p=0.0988)	Race category, TMB, MSI,	Phosphorylase Kinase Regulatory Su	Phosphorylase b kinase catalyzes the phosphorylation of serine in certain substrates, including troponin I. The beta chain acts as a regulatory unit and modulates the activity of the holoenzyme in response to phosphorylation.
1:161476597-Ins		FCGR2A	intron_variant	Other	0.15	18.72	27397079.00	4.50E-02	Dis free p=0.0000429	MSI, TMB	Fc Gamma Receptor IIa	By binding to IgG it initiates cellular responses against pathogens and soluble antigens.
17:29576977-Ins		NF1	intron_variant	Other	0.15	18.72	27397079.00	4.50E-02	no	MSI, TMB, race category, microsc	Neurofibromin 1	Stimulates the GTPase activity of Ras. appears to function as a negative regulator of the ras signal transduction pathway.
12:999542-Ins		WNK1	intron_variant	Other	0.15	18.72	27397079.00	4.50E-02	no	TMB, race category, MSI, CMS,	WNK Lysine Deficient Protein Kinas	Serine/threonine-protein kinase component of the WNK1-SPAK/OSR1 kinase cascade, which acts as a key regulator of blood pressure and regulatory volume increase by promoting ion influx
1:63299891-Del		ATG4C	intron_variant	Other	0.22	19.12	33554432.00	4.58E-02	no	Race category, TMB	Autophagy Related 4C Cysteine Pep	Cysteine protease that plays a key role in autophagy by mediating both proteolytic activation and delipidation of ATG8 family proteins
14:51096508-Ins		ATL1	intron_variant	Other	0.22	19.12	33554432.00	4.58E-02	no	Race category, TMB,	Atlastin GTPase 1	Functions in endoplasmic reticulum tubular network biogenesis
5:78324211-Ins		DMGDH	intron_variant	Other	0.22	19.12	33554432.00	4.58E-02	Prog free p=0.0288	Race category, TMB, MSI, ICR higl	Dimethylglycine Dehydrogenase	an enzyme involved in the catabolism of choline, catalyzing the oxidative demethylation of dimethylglycine to form sarcosine.
22:50927790-Ins		MIOX	intron_variant	Other	0.22	19.12	33554432.00	4.58E-02	Overall p=0.0122	MSI	Myo-Inositol Oxygenase	nvolved in inositol catabolic process.
7:95221694-Ins		PDK4	intron_variant	Other	0.22	19.12	33554432.00	4.58E-02	no	Race category, TMB, MSI	Pyruvate Dehydrogenase Kinase 4	located in the matrix of the mitochondria and inhibits the pyruvate dehydrogenase complex by phosphorylating one of its subunits, thereby contributing to the regulation of glucose metabolism
13:79940644-Ins-3		RBM26	intron_variant	Other	0.22	19.12	33554432.00	4.58E-02	no	TMB, MSI,	RNA Binding Motif Protein 26	Predicted to be involved in mRNA processing.
3:27431393-Ins		SLC4A7	intron_variant	Other	0.22	19.12	33554432.00	4.58E-02	no (dis free p=0.0663)	MSI, race category, TMB,	Solute Carrier Family 4 Member 7	a sodium bicarbonate cotransporter.
13:32892973-Ins		BRCA2	intron_variant	Other	0.19	18.49	19372660.35	4.59E-02	no	MSI, TMB, CIMP, sex, BMI, CMS,	BRCA2 DNA Repair Associated	Involved in double-strand break repair and/or homologous recombination.
10:85984006-Ins		LRIT2	intron_variant	Other	0.19	18.74	27397079.00	4.59E-02	no	TMB, race category,	Leucine Rich Repeat, Ig-Like And Tr	Predicted to be integral component of membrane.
12:32963562-Ins		PKP2	intron_variant	Other	0.19	18.49	19372660.35	4.59E-02	no (overall p=0.0830)	MSI, race category, TMB,	Plakophilin 2	Regulates focal adhesion turnover resulting in changes in focal adhesion size, cell adhesion and cell spreading, potentially via transcriptional modulation of beta-integrins
3:132295651-Ins		ACAD11,NPHP3-ACAD11	intron_variant	Other	0.19	18.77	27397079.00	4.59E-02				
6:38810040-Ins		DNAH8	intron_variant	Other	0.19	18.49	19372660.35	4.59E-02	no (dis free p=0.0554)	MSI, race category, TMB, CIMP, C	Dynein Axonemal Heavy Chain 8	a heavy chain of an axonemal dynein involved in sperm and respiratory cilia motility.
9:103309971-Ins		MSANTD3-TMEFF1,TMEFF1	intron_variant	Other	0.19	18.49	19372660.35	4.59E-02				
8:38172087-Del		NSD3	intron_variant	Other	0.19	18.74	27397079.00	4.59E-02	Overall p=0.0207	MSI, TMB, sex,	Nuclear Receptor Binding SET Dom	methylates histone H3 at lysine residues 4 and 27, which represses gene transcription.
2:85510481-Ins		TCF7L1	intron_variant	Other	0.19	18.74	27397079.00	4.59E-02	no	Race category, TMB, MSI,	Transcription Factor 7 Like 1	Participates in the Wnt signaling pathway, contains a high mobility group-box DNA binding domain and participates in the regulation of cell cycle genes and cellular senescence.
20:31292299-Del	rs59379723	COMMD7	splice_region_variant	Other	0.20	18.53	23726566.41	4.64E-02	no	TMB, MSI, CMS	COMM Domain Containing 7	May modulate activity of cullin-RING E3 ubiquitin ligase (CRL) complexes, Associates with the NF-kappa-B complex and suppresses its transcriptional activity
12:76442907-SNV	rs11180815	NAP1L1	3_prime_UTR_variant	Other	0.20	18.53	23726566.41	4.64E-02	Prog free p=0.0381	Race category, TMB, MSI,	Nucleosome Assembly Protein 1 Lik	participates in DNA replication and may play a role in modulating chromatin formation and contribute to the regulation of cell proliferation.
19:19004466-Del		CERS1,GDF1	intron_variant	Other	0.21	18.75	27397079.00	4.65E-02				
1:225541605-Ins		DNAH14	intron_variant	Other	0.21	18.75		4.65E-02	no	Race category, TMB, MSI, CMS,	Dynein Axonemal Heavy Chain 14	an axonemal dynein heavy chain
14:60475025-Ins		LRRC9	intron_variant	Other	0.21	18.75	27397079.00	4.65E-02	no (prog free p=0.0533)	TMB, CMS	Leucine Rich Repeat Containing 9	An important paralog of this gene is LRRCC1 that is a centrosomal protein
16:67899265-Del		NUTF2	intron_variant	Other	0.21	18.75	27397079.00	4.65E-02	no	MSI	Nuclear Transport Factor 2	plays indirectly a more general role in cargo receptor-mediated nucleocytoplasmic transport.
10:99139583-Del		RRP12	intron_variant	Other	0.21	18.75	27397079.00	4.65E-02	no	Race category, MSI, TMB, CMS1 a	Ribosomal RNA Processing 12 Hom	Predicted to be involved in rRNA processing.
1:118634155-Ins		SPAG17	intron_variant	Other	0.21	18.75	27397079.00	4.65E-02	no	TMB, race category, MSI, CIMP,	Sperm Associated Antigen 17	a central pair protein present in the axonemes of cells with a "9 + 2" organization of microtubules.
12:66638665-Ins		IRAK3	intron_variant	Other	0.21	18.75		4.65E-02	no	Race category, TMB, MSI,	Interleukin 1 Receptor Associated K	Putative inactive protein kinase which regulates signaling downstream of immune receptors including IL1R and Toll-like receptors
11:26529951-Del		ANO3	intron_variant	Other	0.25	18.77	27397079.00	4.73E-02	no	TMB, race category, MSI,	Anoctamin 3	little is known about the function
15:76222522-Del		FBXO22	3_prime_UTR_variant	Other	0.25	18.77	27397079.00	4.73E-02	no	Race category, TMB	F-Box Protein 22	Substrate-recognition component of the SCF (SKP1-CUL1-F-box protein)-type E3 ubiquitin ligase complex.
11:10514803-Ins		AMPD3	intron_variant	Other	0.25	18.77	27397079.00	4.73E-02	no	Race category, TMB, MSI, micro s	Adenosine Monophosphate Deami	a highly regulated enzyme that catalyzes the hydrolytic deamination of adenosine monophosphate to inosine monophosphate, a branch point in the adenylate catabolic pathway.
4:10559856-Ins		CLNK	intron_variant	Other	0.25	18.77	27397079.00	4.73E-02	no (dis specific p=0.060)	Race category, TMB	Cytokine Dependent Hematopoieti	An adapter protein which plays a role in the regulation of immunoreceptor signaling,
8:33229978-Ins		FUT10	3_prime_UTR_variant	Other	0.25	18.77	27397079.00	4.73E-02	no		Fucosyltransferase 10	Predicted to be involved in fucosylation. Predicted to act upstream of or within cerebral cortex radially oriented cell migration and neuronal stem cell population maintenance.
8:97154319-Ins		GDF6	downstream_gene_variant	Unknown	0.25	18.77	27397079.00	4.73E-02	Prog free p=0.004	MSI, CIMP, ethinicity	Growth Differentiation Factor 6	Growth factor that controls proliferation and cellular differentiation in the retina and bone formation. Plays a key role in regulating apoptosis during retinal development.
3:33076098-Ins		GLB1	intron_variant	Other	0.25	18.77	27397079.00	4.73E-02	Overall p=0.0218	Race category, TMB, MSI, CIMP	Galactosidase Beta 1	catalyzes the hydrolysis of a terminal beta-linked galactose residue from ganglioside substrates and other glycoconjugates.
5:93987396-Ins		SLF1	intron_variant	Other	0.25	18.77	27397079.00	4.73E-02	no	TMB, race category, CMS, MSI	SMC5-SMC6 Complex Localization	Plays a role in the DNA damage response (DDR) pathway by regulating postreplication repair of UV-damaged DNA and genomic stability maintenance
7:138189190-Del	rs763671174	TRIM24	intron_variant	Other	0.25	18.77	27397079.00	4.73E-02	no	Race category, TMB, MSI	Tripartite Motif Containing 24	mediates transcriptional control by interaction with the activation function 2 (AF2) region of several nuclear receptors, including the estrogen, retinoic acid, and vitamin D3 receptors.
10:226163-Del		ZMYND11	intron_variant	Other	0.25	18.77	27397079.00	4.73E-02	Overall p=0.0263	TMB, race category, MSI,	Zinc Finger MYND-Type Containing	Chromatin reader that specifically recognizes and binds histone H3.3 trimethylated at 'Lys-36' (H3.3K36me3) and regulates RNA polymerase II elongation.
7:87146048-Del	rs546527484	ABCB1	intron_variant	Other	0.40	18.66	23726566.41	4.83E-02	Overall p=0.0007919	Race category, TMB, MSI,	ATP Binding Cassette Subfamily B M	Translocates drugs and phospholipids across the membrane
11:61071219-Ins		DDB1	intron_variant	Other	0.30	18.80	27397079.00	4.83E-02	no (dis free p=0.0682)	MSI, race category, TMB, CMS, CII	Damage Specific DNA Binding Prote	functions in nucleotide-excision repair and binds to DNA following UV damage.
2:121555236-Ins	rs58254611	GLI2	intron_variant	Other	0.30	18.80	27397079.00	4.83E-02	no (prog free p=0.0757)	MSI, race category, TMB,	GLI Family Zinc Finger 2	Functions as transcription regulator in the hedgehog (Hh) pathway
6:149982749-Ins		LATS1	3_prime_UTR_variant	Other	0.40	18.66	23726566.41	4.83E-02	no (dis specific p=0.0885)	Race category, TMB, MSI, sex,	Large Tumor Suppressor Kinase 1	a putative serine/threonine kinase that localizes to the mitotic apparatus and complexes with cell cycle controller CDC2 kinase in early mitosis. a role as a tumor suppressor suggested.
4:4270106-Ins		LYAR	intron_variant	Other	0.30	18.80	27397079.00	4.83E-02	Prog free p=0.0307, dis fr	Race category, TMB, MSI	Ly1 Antibody Reactive	DNA-binding transcription factor binding activity; identical protein binding activity; and transcription regulator inhibitor activity.
5:37026201-Ins		NIPBL	intron_variant	Other	0.30	18.80	27397079.00	4.83E-02	no	MSI, race category, TMB, CIMP,CI	NIPBL Cohesin Loading Factor	Plays an important role in the loading of the cohesin complex on to DNA.
1:17420276-Del		PADI2	intron_variant	Other	0.30	18.80	27397079.00	4.83E-02	no	TMB, MSI	Peptidyl Arginine Deiminase 2	Catalyzes the deimination of arginine residues of proteins.
11:67160413-Ins		RAD9A	intron_variant	Other	0.30	18.80	27397079.00	4.83E-02	no	MSI, race category, TMB,	RAD9 Checkpoint Clamp Componen	Component of the 9-1-1 cell-cycle checkpoint response complex that plays a major role in DNA repair.
4:71628083-Ins		RUFY3	intron_variant	Other	0.30	18.80	27397079.00	4.83E-02	no	TMB, MSI,	RUN And FYVE Domain Containing	Plays a role in the generation of neuronal polarity formation and axon growth
6:152711928-Ins		SYNE1	intron_variant	Other	0.30	18.80	27397079.00	4.83E-02	see line 113			
3:9825555-Ins		TADA3	3_prime_UTR_variant	Other	0.30	18.80	27397079.00	4.83E-02	no	Race category, TMB, MSI	Transcriptional Adaptor 3	a component of the PCAF complex that is capable of efficiently acetylating histones in a nucleosomal context.
10:73565338-Ins		CDH23	intron_variant	Other	0.14	19.01	33554432.00	4.99E-02	no	MSI, race category, TMB, CMS, CII	Cadherin Related 23	CDH23 is required for establishing and/or maintaining the proper organization of the stereocilia bundle of hair cells in the cochlea and the vestibule during late embryonic/early postnatal development.
5:70939332-Del		MCCC2	intron_variant	Other	0.14	19.01	33554432.00	4.99E-02	no	Race category, TMB, MSI, sex,	Methylcrotonyl-CoA Carboxylase S	Carboxyltransferase subunit of the 3-methylcrotonyl-CoA carboxylase, an enzyme that catalyzes the conversion of 3-methylcrotonyl-CoA to 3-methylglutaconyl-CoA, a critical step for leucine and isovaleric acid catabolism.
4:71634492-Del		RUFY3	intron_variant	Other	0.14	19.01	33554432.00	4.99E-02	see line 196			

Supplementary Table 1a White

Identifier	Gene Names	Sequence Ontology	Effect	MAF	Beta	log2FoldChang	P-Value	survival influence (p<0	other notables p<0.05	full gene name	protein and function	CRC publication#	Cancer pub #	All publication#
rs3215471	SCGN	5_prime_UTR_variant	Other	0.40	-1.84	-0.05620146	3.54E-03	prog free p=0.0343	MSI, TMB, race category	Secretagogin, EF-Hand Calcium Binding Protein	a secreted calcium-binding protein, thought to be involved in KCL-stimulated calcium flux and cel	2	30	126
	GNG4	intron_variant	Other	0.22	18.95	-0.202686388	9.62E-03	prog free p=0.0264	mutation count	G Protein Subunit Gamma 4	Involved in negative regulation of cell growth.	8	35	45
	LIPN	intron_variant	Other	0.31	19.01	-0.286501343	1.09E-02	prog free p=0.0234	Race category, MSI,	Lipase Family Member N	a lipase that is highly expressed in granular keratinocytes in the epidermis, and plays a role in the	0	3	62
rs12567713	FLVCR1	3_prime_UTR_variant	Other	0.20	18.66	-0.080004641	1.49E-02	Dis free p=0.0484	MSI	FLVCR Choline And Heme Transporter 1	a heme transporter that may play a critical role in erythropoiesis by protecting developing erythro	0	43	119
rs667627	FOXRED1	3_prime_UTR_variant	Other	0.20	18.66	0.090232312	1.49E-02	Prog free p=0.0428	Race category, TMB, MSI, ICR high	FAD Dependent Oxidoreductase Domain Containing 1	may function as a chaperone protein required for the function of mitochondrial complex I	0	1	24
rs6537	CHD1L	3_prime_UTR_variant	Other	0.40	19.01	-0.061599522	1.58E-02	Dis free p=0.0476	TMB, MSI	Chromodomain Helicase DNA Binding Protein 1 Like	a DNA helicase protein involved in DNA repair	0	88	111
	NFASC	intron_variant	Other	0.22	19.56	-0.000640884	1.68E-02	Overall p=0.0274	MSI, Race category, TMB, CIMP,	Neurofascin	an L1 family immunoglobulin cell adhesion molecule, functions in neurite outgrowth	0	15	249
	DHA3,PCDHA4,PCDHA5,PCD	splice_region_variant	Other	0.30	19.12		1.92E-02	Prog free p=0.0046	MSI, TMB, CIMP	Protocadherin gene cluster				
	RGS1	upstream_gene_variant	Unknown	0.30	19.12	-0.324572117	1.92E-02	Dis free p=0.00919	Race category, mut count	Regulator Of G Protein Signaling 1	Regulates G protein-coupled receptor signaling cascades	1	76	322
	SFT2D1	intron_variant	Other	0.20	19.01	-0.020484766	1.96E-02	Overall p=0.0103	Race category, MSI	SFT2 Domain Containing 1	May be involved in fusion of retrograde transport vesicles derived from an endocytic compartme	0	4	5
	PLD1	intron_variant	Other	0.18	18.95	-0.138338021	2.18E-02	Dis free p=0.0305	TMB, MSI,	Phospholipase D1	Implicated as a critical step in numerous cellular pathways, including signal transduction, membrar	9	206	832
rs367544298	DNMT3A	intron_variant	Other	0.35	-1.30	0.107987337	2.25E-02	Overall p=0.0217	MSI, race category, TMB, sex, ICR high, micro score,	DNA Methyltransferase 3 Alpha	a DNA methyltransferase that is thought to function in de novo methylation, rather than maintena	54	2467	4499
	MYO1B	intron_variant	Other	0.25	19.46	-0.099018815	2.29E-02	Overall p=0.00253	MSI, race category, TMB, CMS1	Myosin IB	Involved in actin filament organization and post-Golgi vesicle-mediated transport.	1	35	84
	XDH	downstream_gene_variant	Unknown	0.25	19.46	-0.111715589	2.29E-02	Dis specific p=0.0286	MSI, Race category, TMB, CIMP,	Xanthine Dehydrogenase	Key enzyme in purine degradation. contributes to the generation of reactive oxygen species.	3	57	815
	DSCAML1	intron_variant	Other	0.25	19.01	0.074609586	2.29E-02	Overall p=0.00065	MSI, TMB, CMS, CIMP,	DS Cell Adhesion Molecule Like 1	Cell adhesion molecule that plays a role in neuronal self-avoidance	1	6	38
	PCDH10	3_prime_UTR_variant	Other	0.16	18.92	0.058149856	2.37E-02	Overall p=0.0406	MSI, race category, TMB, CIMP, CMS, sex, ICR	Protocadherin 10	a cadherin-related neuronal receptor thought to function in the establishment of specific cell-cell	0	88	151
rs767542195	WDR35	intron_variant	Other	0.24	18.49	-0.202933257	2.41E-02	Dis free p=0.0467	Race category, TMB, MSI,	WD Repeat Domain 35	involved in ciliogenesis and ciliary protein trafficking	0	8	63
	POLQ	intron_variant	Other	0.21	18.94	-0.252403164	2.55E-02	Overall p=0.0491	MSI, race category, TMB, CMS1 and 4, CIMP	DNA Polymerase Theta	DNA polymerase that promotes microhomology-mediated end-joining (MMEJ), an alternative no	4	136	193
rs61738284	MKI67	missense_variant	Missense	0.17	18.66	-0.200139071	2.72E-02	Dis free p=0.0453	MSI, race category, TMB, CIMP, ICR high, CMS1, sex	Marker Of Proliferation Ki-67	has a high net electrical charge and acts as a surfactant, dispersing chromosomes and enabling inc	28	933	1318
	MTHFSD	intron_variant	Other	0.19	18.89	0.076645439	2.75E-02	Prog free p=0.0493	MSI, TMB	Methenyltetrahydrofolate Synthetase Domain Containing	Enables RNA binding activity	0	1	6
	COL11A1	intron_variant	Other	0.18	18.66	-0.354833663	2.82E-02	Overall p=0.0322	MSI, race category, TMB, CMS1, CIMP	Collagen Type XI Alpha 1 Chain	May play an important role in fibrillogenesis by controlling lateral growth of collagen II fibrils.	18	239	591
	MUC16	intron_variant	Other	0.33	18.76	-0.19095091	2.97E-02	Dis free p=0.0295	MSI, race category, TMB, CMS1 and 3, ICR high,	Mucin 16, Cell Surface Associated	thought to play a role in forming a barrier, protecting epithelial cells from pathogens. Products of	28	1113	1377
	FRYL	intron_variant	Other	0.33	19.01	-0.280691013	2.97E-02	Overall p=0.045	MSI, race category, TMB, CMS1, CIMP,	FRY Like Transcription Coactivator	Plays a key role in maintaining the integrity of polarized cell extensions during morphogenesis, re	0	7	18
	FBXW12	intron_variant	Other	0.15	18.84	-0.243525124	3.03E-02	Prog free p=0.0260	Race category, TMB	F-Box And WD Repeat Domain Containing 12	Substrate-recognition component of the SCF (SKP1-CUL1-F-box protein)-type E3 ubiquitin ligase	0	3	6
rs16971526	DNAH17	missense_variant	Missense	0.16	18.61	0.095539119	3.22E-02	Overall p=0.00475	MSI, race category, TMB, CIMP, ICR high, CMS	Dynein Axonemal Heavy Chain 17	Plays a major role in sperm motility, implicated in sperm flagellar assembly and beating	0	16	47
	INPP5D	intron_variant	Other	0.27	18.92	0.051346589	3.25E-02	Dis free p=0.00259	Race category, TMB, MSI,	Inositol Polyphosphate-5-Phosphatase D	functions as a negative regulator of myeloid cell proliferation and survival	4	107	373
	DUOX1	intron_variant	Other	0.44	1.18	0.077735593	3.26E-02	Dis free p=0.00272	MSI, TMB, race cetegory, CMS	Dual Oxidase 1	generates hydrogen peroxide and thereby plays a role in the activity of thyroid peroxidase, lactop	23	188	933
	CPS1	intron_variant	Other	0.18	18.80	-0.274611346	3.62E-02	Overall p=0.0242	Race category, TMB, MSI,	Carbamoyl-Phosphate Synthase 1	The mitochondrial enzyme encoded by this gene catalyzes synthesis of carbamoyl phosphate fror	5	133	517
	SALL2	3_prime_UTR_variant	Other	0.18	18.80	0.186086918	3.62E-02	Dis free p=0.0452	MSI, race category, TMB, CIMP	Spalt Like Transcription Factor 2	Probable transcription factor that plays a role in eye development before, during, and after optic	1	51	75
rs11362069	DIDO1	intron_variant	Other	0.29	18.66	0.4624927	3.62E-02	Overall p=0.003238	MSI, TMB, CIMP,	Death Inducer-Obliterator 1	Tumor suppressor. Required for early embryonic stem cell development.	0	33	62
	WDR62	intron_variant	Other	0.16	18.79	0.144751232	3.69E-02	Dis free p=0.0300	MSI, race category, TMB, CIMP,	WD Repeat Domain 62	Required for cerebral cortical development. Plays a role in neuronal proliferation and migration	0	18	125
rs398000295	F13A1	splice_region_variant	Other	0.23	-1.13	-0.226005907	4.12E-02	Dis free p=0.0281	Race category, TMB, MSI,	Coagulation Factor XIII A Chain	encodes the coagulation factor XIII A subunit	0	43	217
	FCGR2A	intron_variant	Other	0.15	18.72	0.126117921	4.50E-02	Dis free p=0.0000429	MSI, TMB	Fc Gamma Receptor IIa	By binding to IgG it initiates cellular responses against pathogens and soluble antigens.	0	132	516
	DMGDH	intron_variant	Other	0.22	19.12	-0.160661248	4.58E-02	Prog free p=0.0288	Race category, TMB, MSI, ICR high	Dimethylglycine Dehydrogenase	an enzyme involved in the catabolism of choline, catalyzing the oxidative demethylation of dimetl	1	12	59
	MIOX	intron_variant	Other	0.22	19.12	0.346119458	4.58E-02	Overall p=0.0122	MSI	Myo-Inositol Oxygenase	nvolved in inositol catabolic process.	0	15	119
	NSD3	intron_variant	Other	0.19	18.74	-0.07335117	4.59E-02	Overall p=0.0207	MSI, TMB, sex,	Nuclear Receptor Binding SET Domain Protein 3	methylates histone H3 at lysine residues 4 and 27, which represses gene transcription.	1	109	134
rs11180815	NAP1L1	3_prime_UTR_variant	Other	0.20	18.53	-0.330809573	4.64E-02	Prog free p=0.0381	Race category, TMB, MSI,	Nucleosome Assembly Protein 1 Like 1	participates in DNA replication and may play a role in modulating chromatin formation and contri	4	49	133
	GDF6	downstream_gene_variant	Unknown	0.25	18.77	0.36400869	4.73E-02	Prog free p=0.004	MSI, CIMP, ethinicity	Growth Differentiation Factor 6	Growth factor that controls proliferation and cellular differentiation in the retina and bone formati	1	11	160
	GLB1	intron_variant	Other	0.25	18.77	-0.056541977	4.73E-02	Overall p=0.0218	Race category, TMB, MSI, CIMP	Galactosidase Beta 1	catalyzes the hydrolysis of a terminal beta-linked galactose residue from ganglioside substrates a	3	42	334
	ZMYND11	intron_variant	Other	0.25	18.77	-0.085319918	4.73E-02	Overall p=0.0263	TMB, race category, MSI,	Zinc Finger MYND-Type Containing 11	Chromatin reader that specifically recognizes and binds histone H3.3 trimethylated at 'Lys-36' (H3	0	42	102
rs546527484	ABCB1	intron_variant	Other	0.40	18.66	-0.129421568	4.83E-02	Overall p=0.0007919	Race category, TMB, MSI,	ATP Binding Cassette Subfamily B Member 1	Translocates drugs and phospholipids across the membrane	200	3979	7448
	LYAR	intron_variant	Other	0.30	18.80	0.000345182	4.83E-02	Prog free p=0.0307, dis	Race category, TMB, MSI	Ly1 Antibody Reactive	DNA-binding transcription factor binding activity; identical protein binding activity; and transcripti	0	34	56

Supplementary Table 1b White SCGs

Predictor	Identifier	Gene Names	Sequence Ontology	Effect	MAF	Beta	SE	P-Value	survival influence (p<0	other notables p<0.05	full gene name	protein and function
11:129772172-SNV	rs1540408	PRDM10	3_prime_UTR_variant	Other	0.22	18.87	21221686.14	2.81E-03	no	MSI, TMB, race category, CIMP hig	PR/SET Domain 10	a transcription factor
3:15498334-Del	rs1297523120	COLQ	intron_variant	Other	0.23	18.96	21221686.14	3.70E-03	no	MSI, TMB, race category, methyla	Collagen Like Tail Subunit Of Asymmetri	a collagen-like molecule
1:155707000-Del		DAP3	intron_variant	Other	0.27	19.63	27397079.00	3.77E-03	no	MSI, mutation count, methylation	Death Associated Protein 3	Mammalian mitochondrial 28S ribosomal protein, apoptosis
1:19972671-Del	rs142481240	MICOS10-NBL1,NBL1	intron_variant	Other	0.27	19.63	27397079.00	3.77E-03	ND insufficient sample number		MICOS10-NBL1 Readthrough	read-through transcripts, component of the MICOS complex, a large protein complex of the mitochondrial inner membrane
11:48510654-SNV	rs7103557	OR4A47	missense_variant	Missense	0.21	18.93	21221686.14	4.05E-03	no	mutation count, TMB,	Olfactory Receptor Family 4 Subfamily A	Odorant receptor
1:144917841-SNV	rs1698681	PDE4DIP	missense_variant	Missense	0.21	18.93	21221686.14	4.05E-03	no	MSI, TMB, race category	Phosphodiesterase 4D Interacting Prote	to anchor phosphodiesterase 4D to the Golgi/centrosome region
10:93593551-Ins		TNKS2	intron_variant	Other	0.22	19.12	23726566.41	4.73E-03	no	MSI, TMB, race category,	Tankyrase 2	protein ADP-ribosylation
10:91479123-Ins-2		KIF20B	intron_variant	Other	0.21	19.41	27397079.00	5.17E-03	no	TMB, MSI, race category,	Kinesin Family Member 20B	plus-end-directed microtubule motor
2:231305222-Ins		SP100	5_prime_UTR_variant	Other	0.30	19.56	27397079.00	5.47E-03	no (dis free p=0.0867)	Race category, MSI, TMB,	SP100 Nuclear Antigen	tumor suppressor, a major constituent of the PML bodies
19:57988667-Ins	rs34678661	ZNF772	inframe_insertion	Missense	0.33	1.37	0.60	6.64E-03	no	Mutation count, TMB,	Zinc Finger Protein 772	regulation of transcription by RNA polymerase II.
9:97365939-Del		FBP1	intron_variant	Other	0.19	19.21	27397079.00	7.60E-03	no	MSI, TMB, race category	Fructose-Bisphosphatase 1	a gluconeogenesis regulatory enzyme
17:62130397-Ins		ERN1	intron_variant	Other	0.46	-1.70	0.77	9.13E-03	no	MSI, TMB	Endoplasmic Reticulum To Nucleus Sign	transmembrane protein kinase inositol-requiring enzyme 1
1:213178828-Ins		ANGEL2	intron_variant	Other	0.18	18.64	16777216.00	1.11E-02	Dis free p=0.0263	Race category, MSI, TMB, CIMP	Angel Homolog 2	Involved in 3'-UTR-mediated mRNA stabilization and negative regulation of mitotic cell cycle
10:70099332-Del	rs148334349	HNRNPH3	intron_variant	Other	0.18	18.64	16777216.00	1.11E-02	no (overall p=0.0634)	MSI, TMB	Heterogeneous Nuclear Ribonucleoprot	Involved in the splicing process and participates in early heat shock-induced splicing arrest
5:850774-Del	rs67067158	ZDHHC11	5_prime_UTR_variant	Other	0.18	18.64	16777216.00	1.11E-02	no	Race category, mutation count	Zinc Finger DHHC-Type Containing 11	Endoplasmic reticulum-localized palmitoyltransferase, DNA virus-triggered and CGAS-mediated innate immune response
1:92573371-Ins		BTBD8	intron_variant	Other	0.18	19.12	27397079.00	1.22E-02	Overall p=0.0274	Race category, TMB, Micro score	BTB Domain Containing 8	Involved in clathrin-mediated endocytosis at the synapse.
14:91773656-Del		CCDC88C	intron_variant	Other	0.18	19.12	27397079.00	1.22E-02	no	MSI, TMB, CMS1,	Coiled-Coil Domain Containing 88C	a negative regulator of the Wnt signalling pathway
1:32689478-Ins		EIF3I	intron_variant	Other	0.20	19.63	33554432.00	1.29E-02	no	Race category, TMB, MSI	Eukaryotic Translation Initiation Factor	Involved in translational initiation
12:23915548-Ins	rs71059921	SOX5	intron_variant	Other	0.38	19.35	27397079.00	1.36E-02	no	TMB, race category, CIMP, MSI	SRY-Box Transcription Factor 5	Transcription factor involved in chondrocytes differentiation and cartilage formation
1:150780608-Ins		CTSK	intron_variant	Other	0.19	18.84	23726566.41	1.41E-02	no (Dis free p= 0.0995)	Race category, MSI	Cathepsin K	a protease, May play an important role in extracellular matrix degradation.
10:88556184-Ins		BMPR1A	intron_variant	Other	0.31	18.75		1.42E-02	Overall p=0.0238	TMB, MSI, BMI	Bone Morphogenetic Protein Receptor	On ligand binding, forms a receptor complex consisting of two type II and two type I transmembrane serine/threonine kinases.
11:117886314-Ins-2		SMIM35	intron_variant	Other	0.16	19.05	27397079.00	1.44E-02	Prog free p=0.009	Race category	Small Integral Membrane Protein 35	Predicted to be integral component of membrane
12:120580211-Ins		GCN1	intron_variant	Other	0.40	19.01	23726566.41	1.58E-02	no (dis free p=0.0832)	MSI, race category, TMB, CIMP, IC	GCN1 Activator Of EIF2AK4	Directly binds to the ribosome and acts as a sentinel for colliding ribosomes
3:16253968-Ins-2		GALNT15	intron_variant	Other	0.23	19.08	27397079.00	1.67E-02	no	TMB, race category, MSI, CIMP	Polypeptide N-Acetylgalactosaminyltrar	Catalyzes the initial reaction in O-linked oligosaccharide biosynthesis
3:57269383-Ins		APPL1	intron_variant	Other	0.22	19.34	23726566.41	1.68E-02	Overall p=0.0396	Race category, TMB, MSI, CMS1 ar	Adaptor Protein, Phosphotyrosine Intera	Multifunctional adapter protein that binds to various membrane receptors, nuclear factors and signaling proteins to regulate many processes, such as cell proliferation, immune response, endosomal trafficking and cell metabolism
7:27139792-Ins		HOTAIRM1	non_coding_exon_variant	Other	0.22	19.56	33554432.00	1.68E-02	no	Mut count	HOXA Transcript Antisense RNA, Myeloi	an RNA Gene, associated with Leukemia and High Grade Glioma.
20:32661159-Ins		RALY	intron_variant	Other	0.22	19.56	33554432.00	1.68E-02	no	TMB, MSI, race category, CMS2	RALY Heterogeneous Nuclear Ribonucle	may play a role in pre-mRNA splicing and in embryonic development
10:93600943-Del		TNKS2	intron_variant	Other	0.22	19.56	33554432.00	1.68E-02	no	MSI, race category, TMB, sex, CMS	Tankyrase 2	Poly-ADP-ribosyltransferase involved in various processes such as Wnt signaling pathway, telomere length and vesicle trafficking
3:39116114-Ins		WDR48	intron_variant	Other	0.22	19.56	33554432.00	1.68E-02	no	Race category, MSI, TMB	WD Repeat Domain 48	Regulator of deubiquitinating complexes,
15:77271278-Ins					0.22	19.56	33554432.00	1.68E-02				
2:237076795-SNV	rs140196177	GBX2	5_prime_UTR_variant	Other	0.29	18.61	16777216.00	1.73E-02	no	Race category, MSI, CIMP, TMB	Gastrulation Brain Homeobox 2	May act as a transcription factor for cell pluripotency and differentiation in the embryo.
17:37579508-Ins		MED1	intron_variant	Other	0.17	18.83	19372660.35	1.76E-02	Overall p=0.028	TMB, MSI,	Mediator Complex Subunit 1	Component of the Mediator complex, a coactivator involved in the regulated transcription of nearly all RNA polymerase II-dependent genes.
5:71521792-Ins		MRPS27	intron_variant	Other	0.17	19.01	27397079.00	1.76E-02	Prog free p=0.0235	Race category, TMB, MSI	Mitochondrial Ribosomal Protein S27	RNA-binding component of the mitochondrial small ribosomal subunit (mt-SSU) that plays a role in mitochondrial protein synthesis
16:18839297-Ins		SMG1	intron_variant	Other	0.17	19.01	27397079.00	1.76E-02	Overall p=0.0254	Race category, TMB, MSI, CMS1 ar	SMG1 Nonsense Mediated MRNA Decay	Serine/threonine protein kinase involved in both mRNA surveillance and genotoxic stress response pathways
3:53783636-Del		CACNA1D	intron_variant	Other	0.15	19.41	33554432.00	1.78E-02	no	MSI, Rac category, TMB, CIMP, CN	Calcium Voltage-Gated Channel Subunit	The isoform alpha-1D gives rise to L-type calcium currents.
2:26507566-Ins		HADHB	intron_variant	Other	0.15	19.41	33554432.00	1.78E-02	no	Race category, TMB, MSI,	Hydroxyacyl-CoA Dehydrogenase Trifun	the beta subunit of the mitochondrial trifunctional protein, which catalyzes the last three steps of mitochondrial beta-oxidation of long chain fatty acids.
4:184626015-Ins		TRAPPC11	intron_variant	Other	0.15	19.41	33554432.00	1.78E-02	no	Race category, TMB, MSI,	Trafficking Protein Particle Complex Sub	involved in early stage endoplasmic reticulum-to-Golgi vesicle transport
19:37101428-Ins		ZNF382	intron_variant	Other	0.15	19.41	33554432.00	1.78E-02	Prog free p=0.0439	Race category, TMB, MSI,	Zinc Finger Protein 382	Functions as a sequence-specific transcriptional repressor.
3:53708042-Ins		CACNA1D	intron_variant	Other	0.20	19.01	27397079.00	1.96E-02	(see line 36)			
12:104406462-Ins		GLT8D2	intron_variant	Other	0.20	19.01	27397079.00	1.96E-02	no (overall p=0.0993)	Race category, TMB	Glycosyltransferase 8 Domain Containir	Predicted to enable glycosyltransferase activity. Predicted to be integral component of membrane.
11:5291209-Ins		HBE1	upstream_gene_variant	Unknown	0.20	19.01	27397079.00	1.96E-02	no	TMB, Micro score	Hemoglobin Subunit Epsilon 1	a beta-type chain of early mammalian embryonic hemoglobin.
1:175129682-Ins		KIAA0040	3_prime_UTR_variant	Other	0.20	19.01	27397079.00	1.96E-02	Dis free p=0.0022		KIAA0040	Uncharacterized Protein KIAA0040, Predicted to be integral component of membrane.
4:140624778-Del		MGST2	intron_variant	Other	0.20	19.01	27397079.00	1.96E-02	no	Race category, MSI, TMB	Microsomal Glutathione S-Transferase 2	Catalyzes the glutathione-dependent reduction of lipid hydroperoxides, such as 5-HPETE , catalyzes the conjugation of leukotriene A4 and reduced glutathione to produce leukotriene C4
1:36068784-Ins-2		PSMB2	3_prime_UTR_variant	Other	0.20	19.01	27397079.00	1.96E-02	Overall p=0.0253	MSI, TMB, CMS	Proteasome 20S Subunit Beta 2	Non-catalytic component of the 20S core proteasome complex
12:56962661-Ins		RBMS2	intron_variant	Other	0.20	19.01	27397079.00	1.96E-02	no	TMB, MSI, CIMP	RNA Binding Motif Single Stranded Inter	bind single stranded DNA/RNA, implicated in such diverse functions as DNA replication, gene transcription, cell cycle progression and apoptosis
11:118015028-Del		SCN4B	intron_variant	Other	0.20	19.01	27397079.00	1.96E-02	no (overall p=0.0708)	Race category, TMB, CMS1, MSI	Sodium Voltage-Gated Channel Beta Sul	Modulates channel gating kinetics.
19:21948412-Ins		ZNF100	intron_variant	Other	0.20	19.01	27397079.00	1.96E-02	no	Race category, TMB, MSI	Zinc Finger Protein 100	May be involved in transcriptional regulation.
4:6066508-Ins		JAKMIP1	intron_variant	Other	0.15	18.96	27397079.00	1.96E-02	no (Dis free p=0.0903)	MSI, Race category, TMB,	Janus Kinase And Microtubule Interactir	Associates with microtubules and may play a role in the microtubule-dependent transport of the GABA-B receptor. May play a role in JAK1 signaling and regulate microtubule cytoskeleton rearrangements.
1:20443106-Ins		PLA2G2D	intron_variant	Other	0.17	19.10	23726566.41	2.17E-02	no (overall p=0.0934)	Race category, MSI	Phospholipase A2 Group IID	Secretory calcium-dependent phospholipase A2 that primarily targets extracellular lipids, exerting anti-inflammatory and immunosuppressive functions
16:5517383-Del					0.18	18.95	27397079.00	2.18E-02				
15:44951105-Ins		SPG11	intron_variant	Other	0.18	18.95	27397079.00	2.18E-02	no (dis free p=0.0831)	MSI, race category, TMB, CIMP, CI	SPG11 Vesicle Trafficking Associated, Sp	a potential transmembrane protein that is phosphorylated upon DNA damage
17:7574340-Ins		TP53	intron_variant	Other	0.18	18.95	27397079.00	2.18E-02	Overall p=0.0000015	MSI, race category, CMS, CIMP,	Tumor Protein P53	a tumor suppressor protein containing transcriptional activation, DNA binding, and oligomerization domains.
20:62378693-Ins	rs1199903552	ZBTB46	intron_variant	Other	0.18	18.95	27397079.00	2.18E-02	Overall p=0.0276	MSI, CIMP	Zinc Finger And BTB Domain Containing	Predicted to be involved in regulation of transcription by RNA polymerase II.
6:31378226-Ins	rs200894869	MICA	intron_variant	Other	0.35	1.30	0.64	2.25E-02	no	MSI, TMB	MHC Class I Polypeptide-Related Seque	the highly polymorphic major histocompatability complex class I chain-related protein A.
7:152550448-Del		ACTR3B	intron_variant	Other	0.25	19.46	33554432.00	2.29E-02	no (prog free p=0.0666)	Race category, TMB, MSI, micro sc	Actin Related Protein 3B	Plays a role in the organization of the actin cytoskeleton.
13:32923395-Ins-2		BRCA2	intron_variant	Other	0.25	19.46	33554432.00	2.29E-02	no	MSI, TMB, CIMP, sex, BMI, CMS,	BRCA2 DNA Repair Associated	involved in maintenance of genome stability, specifically the homologous recombination pathway for double-strand DNA repair.
1:9005719-Ins		CA6	upstream_gene_variant	Unknown	0.25	19.46	33554432.00	2.29E-02	Dis free p=0.0478	TMB	Carbonic Anhydrase 6	Reversible hydration of carbon dioxide.found only in salivary glands and saliva.
12:75693580-Ins		CAPS2	intron_variant	Other	0.25	19.46	33554432.00	2.29E-02	no	Race category, TMB, MSI	Calcyphosine 2	a calcium-binding protein with 2 EF-hand motifs
9:74828655-Ins		GDA	intron_variant	Other	0.25	19.46	33554432.00	2.29E-02	Dis specific p=0.038	Race category, MSI, TMB	Guanine Deaminase	Catalyzes the hydrolytic deamination of guanine, producing xanthine and ammonia.
3:128864449-Ins		ISY1,ISY1-RAB43	intron_variant	Other	0.25	19.46	33554432.00	2.29E-02	ND insufficient sample r	Race category, MSI, TMB	ISY1-RAB43 readthrough	naturally occurring read-through transcription between the neighboring ISY1 (ISY1 splicing factor homolog) and RAB43 (RAB43, member RAS oncogene family) gene
12:25185510-Ins		LOC645177	intron_variant	Other	0.25	19.46	33554432.00	2.29E-02	ND insufficient sample r	ND insufficient sample number	lymphoid-restricted membrane prote	function not well known
9:14398786-Ins		NFIB	5_prime_UTR_variant	Other	0.25	19.46	33554432.00	2.29E-02	no	TMB, race category, MSI,	Nuclear Factor I B	Transcriptional activator of GFAP, essential for proper brain development
2:43919600-Ins		PLEKHH2	intron_variant	Other	0.25	19.46	33554432.00	2.29E-02	no (overall p=0.0953)	Race category, TMB, MSI	Pleckstrin Homology, MyTH4 And FERM	Predicted to be involved in negative regulation of actin filament depolymerization
3:164704708-Ins		SI	intron_variant	Other	0.25	19.46	33554432.00	2.29E-02	Dis free p=0.0365	Race category, TMB, CIMP,	Sucrase-Isomaltase	essential for the digestion of dietary carbohydrates including starch, sucrose and isomaltose.
11:121482058-Ins		SORL1	intron_variant	Other	0.25	19.46	33554432.00	2.29E-02	no	MSI, race category, TMB, CIMP, IC	Sortilin Related Receptor 1	Sorting receptor that directs several proteins to their correct location within the cell
1:77092934-Ins		ST6GALNAC3	intron_variant	Other	0.25	19.46	33554432.00	2.29E-02	no	Race category, TMB, MSI, CMS1	ST6 N-Acetylgalactosaminide Alpha-2,6-	Transfers the sialyl group (N-acetyl-alpha-neuraminyl or NeuAc) from CMP-NeuAc to the GalNAc residue on the NeuAc-alpha-2,3-Gal-beta-1,3-GalNAc sequence of glycoproteins and glycolipids forming an alpha-2,6-linkage.
9:116764088-Ins		ZNF618	intron_variant	Other	0.25	19.46	33554432.00	2.29E-02	no	Race category, TMB, MSI, ICR high	Zinc Finger Protein 618	Enables identical protein binding activity and transcription coregulator binding activity.
5:41201902-Del		C6	intron_variant	Other	0.25	19.01	27397079.00	2.29E-02	no (dis specific p=0.059	MSI, race category, TMB, CIMP, CI	Complement C6	Constituent of the membrane attack complex (MAC) that plays a key role in the innate and adaptive immune response by forming pores in the plasma membrane of target cells.
6:41566439-Del		FOXP4	intron_variant	Other	0.25	19.01	27397079.00	2.29E-02	no	Race category, MSI, TMB, CMS1, C	Forkhead Box P4	transcription factor family, may play a role in the development of tumors of the kidney and larynx
10:105163119-Ins		PDCD11	intron_variant	Other	0.25	19.01	27397079.00	2.29E-02	no	MSI, TMB, race category, sex	Programmed Cell Death 11	required for rRNA maturation and generation of 18S rRNA
12:81015823-Del	rs149289370	PTPRQ	intron_variant	Other	0.25	19.46	33554432.00	2.29E-02	no	Race category, TMB, CMS, MSI	Protein Tyrosine Phosphatase Receptor	catalyzes the dephosphorylation of phosphotyrosine and phosphatidylinositol and plays roles in cellular proliferation and differentiation, required for auditory function
9:4575869-Ins		SLC1A1	intron_variant	Other	0.25	19.46	33554432.00	2.29E-02	no	Race category, TMB, MSI, CMS1,	Solute Carrier Family 1 Member 1	Sodium-dependent, high-affinity amino acid transporter that mediates the uptake of L-glutamate and also L-aspartate and D-aspartate
17:61271290-Ins-2		TANC2	intron_variant	Other	0.25	19.01	27397079.00	2.29E-02	no	Race category, TMB, MSI, CMS1, C	Tetratricopeptide Repeat, Ankyrin Repe	Scaffolding protein in the dendritic spines which acts as immobile postsynaptic posts able to recruit KIF1A-driven dense core vesicles to dendritic spines.
20:38774764-Ins	rs61140196				0.25	19.46	33554432.00	2.29E-02				
20:40084337-Ins		CHD6	intron_variant	Other	0.16	18.92	27397079.00	2.37E-02	no	MSI, TMB, race category, sex,	Chromodomain Helicase DNA Binding P	DNA-dependent ATPase that plays a role in chromatin remodeling. Regulates transcription
7:74120881-Ins		GTF2I,LOC101926943	intron_variant	Other	0.16	18.92	27397079.00	2.37E-02				
20:39976084-Ins		LPIN3	intron_variant	Other	0.16	18.92	27397079.00	2.37E-02	no	Race category, MSI	Lipin 3	Magnesium-dependent phosphatidate phosphatase enzyme which catalyzes the conversion of phosphatidic acid to diacylglycerol during triglyceride, phosphatidylcholine and phosphatidylethanolamine biosynthesis therefore regulates fatty acid metabolism.
11:95570806-Ins		MTMR2	intron_variant	Other	0.16	18.92	27397079.00	2.37E-02	no	Race category, TMB, MSI	Myotubularin Related Protein 2	Phosphatase that acts on lipids with a phosphoinositol headgroup.
17:38142763-Ins		PSMD3	intron_variant	Other	0.16	18.92	27397079.00	2.37E-02	Dis specific p=0.0379	MSI, TMB,	Proteasome 26S Subunit, Non-ATPase 3	Component of the 26S proteasome,
6:37281477-Del		TBC1D22B	intron_variant	Other	0.16	18.92	27397079.00	2.37E-02	no	TMB, MSI, CMS1	TBC1 Domain Family Member 22B	May act as a GTPase-activating protein for Rab family protein(s).
4:106534506-Ins		ARHGEF38	intron_variant	Other	0.14	18.64	19372660.35	2.52E-02	no	mut count	Rho Guanine Nucleotide Exchange Facto	May act as a guanine-nucleotide releasing factor.
4:101386749-Ins		EMCN	intron_variant	Other	0.14	18.64	19372660.35	2.52E-02	no (overall p=0.0610)	Race category, TMB	Endomucin	interferes with the assembly of focal adhesion complexes and inhibits interaction between cells and the extracellular matrix.
4:144532776-Ins		FREM3	intron_variant	Other	0.14	18.89	27397079.00	2.52E-02	no	MSI, race category, TMB, CMS,	FRAS1 Related Extracellular Matrix 3	the family of FRAS1/FREM extracellular matrix proteins and may play a role cell adhesion.
1:179503108-Ins		AXDND1	intron_variant	Other	0.14	18.64	19372660.35	2.52E-02	no	Race category, TMB, CMS	Axonemal Dynein Light Chain Domain Co	May be essential for spermiogenesis and male fertility probably by regulating the manchette dynamics, spermatid head shaping and sperm flagellum assembly.
13:73321320-Del		BORA	intron_variant	Other	0.21	18.94	27397079.00	2.55E-02	no (dis free p=0.0551)	MSI	BORA Aurora Kinase A Activator	an activator of the protein kinase Aurora A
18:67614751-Ins		CD226	5_prime_UTR_variant	Other	0.21	18.94	27397079.00	2.55E-02	no	MSI, TMB	CD226 Molecule	Involved in intercellular adhesion, lymphocyte signaling, cytotoxicity and lymphokine secretion mediated by cytotoxic T-lymphocyte (CTL) and NK cell
12:122818749-Del		CLIP1	intron_variant	Other	0.21	18.94	27397079.00	2.55E-02	Dis free p=0.008663	Race category, TMB, MSI,	CAP-Gly Domain Containing Linker Prote	Binds to the plus end of microtubules and regulates the dynamics of the microtubule cytoskeleton.,plays an important role in intracellular vesicle trafficking
19:55589357-Ins		EPS8L1	intron_variant	Other	0.21	18.94	27397079.00	2.55E-02	Prog free p=0.0492	Race category, MSI, TMB, CMS, CI	EPS8 Signaling Adaptor L1	Stimulates guanine exchange activity of SOS1. May play a role in membrane ruffling and remodeling of the actin cytoskeleton.
1:170508176-Ins		GORAB	intron_variant	Other	0.21	18.94	27397079.00	2.55E-02	no (prog free p=0.0886)	Race category, TMB	Golgin, RAB6 Interacting	may function in the secretory pathway
3:4741740-Del		ITPR1	intron_variant	Other	0.21	18.94	27397079.00	2.55E-02	Dis free p=0.0301	MSI, race catgeory,TMB,	Inositol 1,4,5-Trisphosphate Receptor T	an intracellular receptor for inositol 1,4,5-trisphosphate. Upon stimulation, this receptor mediates calcium release from the endoplasmic reticulum.
1:184854065-Ins		NIBAN1	intron_variant	Other	0.21	18.94	27397079.00	2.55E-02	no	Race category, TMB, MSI,	Niban Apoptosis Regulator 1	highly expressed in several cancer cells and may serve as a prognostic marker for certain cancers.may play a role in regulating p53-mediated apoptosis.
2:197738542-Ins		PGAP1	intron_variant	Other	0.21	18.94	27397079.00	2.55E-02	no (overall p=0.0578)	Race category, TMB, MSI,	Post-GPI Attachment To Proteins Inosito	functions early in the glycosylphosphatidylinositol (GPI) biosynthetic pathway, catalyzing the inositol deacylation of GPI.
2:28069803-Ins-2		RBKS	intron_variant	Other	0.21	18.94	27397079.00	2.55E-02	no	Race category, TMB	Ribokinase	phosphorylates ribose to form ribose-5-phosphate in the presence of ATP and magnesium as a first step in ribose metabolism.
2:165755375-Del		SLC38A11	intron_variant	Other	0.21	18.94	27397079.00	2.55E-02	no	Race category, TMB	Solute Carrier Family 38 Member 11	Predicted to be involved in amino acid transmembrane transport.
10:75863319-Ins-3		VCL	intron_variant	Other	0.21	18.94	27397079.00	2.55E-02	no	Race category, TMB, MSI,	Vinculin	a cytoskeletal protein associated with cell-cell and cell-matrix junctions
19:50496360-Del		VRK3	intron_variant	Other	0.21	18.94	27397079.00	2.55E-02	Overall p=0.00992	Race category, MSI, TMB	VRK Serine/Threonine Kinase 3	Inactive kinase that suppresses ERK activity by promoting phosphatase activity of DUSP3 which specifically dephosphorylates and inactivates ERK in the nucleus.
17:3840695-Del	rs34866939	ATP2A3	intron_variant	Other	0.30	1.15	0.60	2.64E-02	Overall p=0.0110	TMB, MSI, CIMP, CMS	ATPase Sarcoplasmic/Endoplasmic Retic	catalyzes the hydrolysis of ATP coupled with the translocation of calcium from the cytosol to the sarcoplasmic reticulum lumen, and is involved in calcium sequestration associated with muscular excitation and contraction.
18:43469684-Ins		EPG5	intron_variant	Other	0.13	18.81	27397079.00	2.65E-02	no	MSI, TMB, CMS, CIMP	Ectopic P-Granules 5 Autophagy Tetheri	functions in autophagy during starvation conditions
6:79657194-Ins		PHIP	intron_variant	Other	0.19	18.89	27397079.00	2.75E-02	no	MSI, race category, TMB, CIMP,	Pleckstrin Homology Domain Interacting	binds to the insulin receptor substrate 1 protein and regulates glucose transporter translocation in skeletal muscle cells.
22:42310078-Ins-2		SHISA8	intron_variant	Other	0.19	18.89	27397079.00	2.75E-02	no	mut count	Shisa Family Member 8	May regulate trafficking and current kinetics of AMPA-type glutamate receptor (AMPAR) at synapses.
10:75855988-Del		VCL	intron_variant	Other	0.19	18.89	27397079.00	2.75E-02	see line 96			
4:100336567-Ins		ADH7	intron_variant	Other	0.19	18.89	27397079.00	2.75E-02	no	Race category, MSI, TMB	Alcohol Dehydrogenase 7 (Class IV), Mu	may participate in the synthesis of retinoic acid, a hormone important for cellular differentiation
5:41199711-Ins		C6	intron_variant	Other	0.19	18.89	27397079.00	2.75E-02	see line 69			
1:47842518-Del		CMPK1	3_prime_UTR_variant	Other	0.19	18.89	27397079.00	2.75E-02	no	TMB	Cytidine/Uridine Monophosphate Kinas	Plays an important role in de novo pyrimidine nucleotide biosynthesis.
2:47602201-Ins		EPCAM	intron_variant	Other	0.19	18.89	27397079.00	2.75E-02	no	Race category, MSI, TMB,	Epithelial Cell Adhesion Molecule	May act as a physical homophilic interaction molecule, a carcinoma-associated antigen
5:127612084-Del		FBN2	intron_variant	Other	0.19	18.89	27397079.00	2.75E-02	no	MSI, TMB, race category, CIMP, CI	Fibrillin 2	a component of connective tissue microfibrils and may be involved in elastic fiber assembly
9:79319596-Ins		PRUNE2	intron_variant	Other	0.19	18.89	27397079.00	2.75E-02	Dis free p=0.0274	MSI, race category, TMB, CIMP, CI	Prune Homolog 2 With BCH Domain	May play an important role in regulating differentiation, survival and aggressiveness of the tumor cells.
1:29018312-Del		GMEB1	intron_variant	Other	0.33	19.01	27397079.00	2.97E-02	no	TMB, MSI, CMS, CIMP	Glucocorticoid Modulatory Element Bin	modulate the transactivation of the glucocorticoid receptor when it is bound to glucocorticoid response elements.
16:70995731-Ins		HYDIN	intron_variant	Other	0.33	19.01	27397079.00	2.97E-02	no	MSI, race category, TMB, CIMP	HYDIN Axonemal Central Pair Apparatus	Required for ciliary motility.
1:65332389-Del		JAK1	intron_variant	Other	0.33	19.01	27397079.00	2.97E-02	no	MSI, race category, TMB, sex, CIM	Janus Kinase 1	Tyrosine kinase of the non-receptor type, involved in the IFN-alpha/beta/gamma signal pathway, a key component of the interleukin-6 (IL-6)/JAK1/STAT3 immune and inflammation response
7:150693421-Ins-2		NOS3	intron_variant	Other	0.33	19.01	27397079.00	2.97E-02	no (dis free p=0.0520)	Race category, TMB, MSI, CMS	Nitric Oxide Synthase 3	Produces nitric oxide (NO) which is implicated in vascular smooth muscle relaxation through a cGMP-mediated signal transduction pathway
13:78211387-Del-2	rs755741313	SCEL	intron_variant	Other	0.33	19.01	27397079.00	2.97E-02	no	TMB, race category, MSI	Sciellin	a precursor to the cornified envelope of terminally differentiated keratinocytes.
19:8495843-Ins		MARCHF2	intron_variant	Other	0.15	18.84	27397079.00	3.03E-02	no	Race category, MSI, TMB	Membrane Associated Ring-CH-Type Fin	MARCH family of membrane-bound E3 ubiquitin ligase, may regulate early endosome-to-trans-Golgi network (TGN) trafficking
16:3594163-Del	rs60164526	NLRC3	intron_variant	Other	0.41	-1.15	0.58	3.10E-02	no	Race category, TMB, MSI	NLR Family CARD Domain Containing 3	a cytosolic regulator of innate immunity, directly interacts with stimulator of interferon genes (STING), to prevent its proper trafficking, resulting in disruption of STING-dependent activation of the innate immune response
7:73483689-SNV	rs8326	ELN	3_prime_UTR_variant	Other	0.38	-0.97	0.49	3.12E-02	no (overall p=0.0973)	MSI, TMB, CIMP	Elastin	Major structural protein of tissues such as aorta and nuchal ligament, which must expand rapidly and recover completely.
7:137092746-Del	rs3217275	DGKI	splice_region_variant	Other	0.14	18.82	27397079.00	3.13E-02	no	Race category, MSI, TMB, CIMP,	Diacylglycerol Kinase Iota	may play a crucial role in the production of phosphatidic acid in the retina or in recessive forms of retinal degeneration.
16:67318608-Ins		PLEKHG4	splice_region_variant	Other	0.14	18.82	27397079.00	3.13E-02	Overall p=0.001095	Race category, TMB, MSI, ICR high	Pleckstrin Homology And RhoGEF Doma	can function as a guanine nucleotide exchange factor (GEF) and may play a role in intracellular signaling and cytoskeleton dynamics at the Golgi apparatus.
1:12304296-Ins		VPS13D	splice_region_variant	Other	0.14	18.82	27397079.00	3.13E-02	no	MSI, TMB, race category, CMS, sex	Vacuolar Protein Sorting 13 Homolog D	In yeast, vacuolar-protein-sorting-13 proteins are involved in trafficking of membrane proteins between the trans-Golgi network and the prevacuolar compartment.
17:74684401-SNV	rs2286587	MXRA7	missense_variant	Missense	0.13	18.80	27397079.00	3.22E-02	no	MSI, TMB	Matrix Remodeling Associated 7	Located in endoplasmic reticulum.
1:18704098-Del		IGSF21	intron_variant	Other	0.27	18.92	27397079.00	3.25E-02	no	Race category, MSI, TMB, CMS	Immunoglobin Superfamily Member 21	Involved in synaptic inhibition in the brain.
1:156087782-Del		LMNA	intron_variant	Other	0.27	18.92	27397079.00	3.25E-02	Dis free p=0.0236	Race category, MSI, TMB, CIMP, C	Lamin A/C	part of the nuclear lamina
5:58272398-Ins		PDE4D	intron_variant	Other	0.27	18.92	27397079.00	3.25E-02	Overall p=0.0258	TMB	Phosphodiesterase 4D	has 3',5'-cyclic-AMP phosphodiesterase activity and degrades cAMP, which acts as a signal transduction molecule in multiple cell types
11:129772066-Ins		PRDM10	3_prime_UTR_variant	Other	0.27	18.92	27397079.00	3.25E-02	no	MSI, TMB, race category, CIMP, se	PR/SET Domain 10	a transcription factor that contains C2H2-type zinc-fingers, may be involved in the development of the central nerve system (CNS), as well as in the pathogenesis of neuronal storage disease.
11:117969558-Ins		TMPRSS4	intron_variant	Other	0.27	18.92	27397079.00	3.25E-02	no	Race category, MSB, TMB,	Transmembrane Serine Protease 4	a gene overexpressed in pancreatic carcinoma, Plasma membrane-anchored serine protease
6:31378229-Ins	rs56728307	MICA	intron_variant	Other	0.38	1.23	0.65	3.28E-02	see line 55			
19:48184643-Del	rs58014830	BICRA	intron_variant	Other	0.29	-1.24	0.65	3.32E-02	no (dis specific p=0.090	MSI, race category, TMB, CIMP, CI	BRD4 Interacting Chromatin Remodeling	Component of SWI/SNF chromatin remodeling subcomplex GBAF that carries out key enzymatic activities, changing chromatin structure by altering DNA-histone contacts within a nucleosome in an ATP-dependent manner, May play a role in BRD4-mediated gene
11:108186360-Ins		ATM,C11orf65	intron_variant	Other	0.15	18.94	23726566.41	3.53E-02				
1:40776148-Ins		COL9A2	intron_variant	Other	0.15	19.15	33554432.00	3.53E-02	no	Race category, TMB, MSI, CIMP	Collagen Type IX Alpha 2 Chain	one of the three alpha chains of type IX collagen, the major collagen component of hyaline cartilage.
12:2904179-Ins		FKBP4,ITFG2-AS1	5_prime_UTR_variant	Other	0.15	19.15	33554432.00	3.53E-02				
12:123343235-Ins		HIP1R	intron_variant	Other	0.15	18.90	23726566.41	3.53E-02	no (dis free p=0.0590)	MSI, TMB, race category, CIMP,	Huntingtin Interacting Protein 1 Related	Component of clathrin-coated pits and vesicles, that may link the endocytic machinery to the actin cytoskeleton.
1:211923219-Ins		LPGAT1	3_prime_UTR_variant	Other	0.15	19.15	33554432.00	3.53E-02	Dis free p=0.0159	MSI	Lysophosphatidylglycerol Acyltransferas	Lysophospholipid acyltransferase involved in fatty acyl chain remodeling of glycerophospholipids in the endoplasmic reticulum membrane
21:42817284-Ins		MX1	intron_variant	Other	0.15	19.15	33554432.00	3.53E-02	no	Race category, TMB, MSI, CMS,	MX Dynamin Like GTPase 1	Interferon-induced dynamin-like GTPase with antiviral activity against a wide range of RNA viruses and some DNA viruses.
5:170341297-Ins		RANBP17	intron_variant	Other	0.15	19.15	33554432.00	3.53E-02	no	Race category, TMB, MSI	RAN Binding Protein 17	May function as a nuclear transport receptor.
22:42271255-Ins		SREBF2	intron_variant	Other	0.15	19.15	33554432.00	3.53E-02	no	Race category, TMB, MSI, CIMP,	Sterol Regulatory Element Binding Trans	Key transcription factor that regulates expression of genes involved in cholesterol biosynthesis
18:72167381-Del		CNDP2	intron_variant	Other	0.15	19.15	33554432.00	3.53E-02	no	MSI, TMB, race category, CIMP,	Carnosine Dipeptidase 2	a nonspecific dipeptidase, Plays a role in the regulation of cell cycle arrest and apoptosis
3:64641615-Ins		ADAMTS9	intron_variant	Other	0.20	18.58	19372660.35	3.54E-02	no (prog free p=0.0747)	MSI, TMB, race category, CIMP, CI	ADAM Metallopeptidase With Thrombo	Cleaves the large aggregating proteoglycans, aggrecan and versican, Has a protease-independent function in promoting the transport from the endoplasmic reticulum to the Golgi apparatus of a variety of secretory cargos.
1:24137067-Ins-3		HMGCL	intron_variant	Other	0.20	18.83	27397079.00	3.54E-02	no	mut count	3-Hydroxy-3-Methylglutaryl-CoA Lyase	a mitochondrial enzyme that catalyzes the final step of leucine degradation and plays a key role in ketone body formation.
22:38042705-Ins		PDXP-DT,SH3BP1	intron_variant	Other	0.20	18.58	19372660.35	3.54E-02				
3:8775923-Ins		CAV3	intron_variant	Other	0.13	19.12	33554432.00	3.56E-02	no	Race category, TMB	Caveolin 3	May act as a scaffolding protein within caveolar membranes. Interacts directly with G-protein alpha subunits and can functionally regulate their activity.
8:70539623-Del		SULF1	intron_variant	Other	0.13	19.12	33554432.00	3.56E-02	no	MSI, TMB, race category, CIMP,	Sulfatase 1	an extracellular heparan sulfate endosulfatase.
1:156095195-Del	rs111616133	LMNA	intron_variant	Other	0.18	18.80	27397079.00	3.62E-02	see line 122			
12:41421525-Ins		CNTN1	intron_variant	Other	0.18	18.80	27397079.00	3.62E-02	no	Race category, TMB, MSI, CIMP, IC	Contactin 1	mediate cell surface interactions during nervous system development.
22:25150663-Ins		PIWIL3	intron_variant	Other	0.18	18.80	27397079.00	3.62E-02	no	Race category, TMB, MSI, CMS1,	Piwi Like RNA-Mediated Gene Silencing	thought to function in maintenance of germline cells.
3:15498335-Del	rs141471889	COLQ	intron_variant	Other	0.26	1.11	0.60	3.68E-02	see line 3			
6:38749240-Del		DNAH8	intron_variant	Other	0.16	18.79	27397079.00	3.69E-02	no (dis free p=0.0554)	MSI, race category, TMB, CIMP, CI	Dynein Axonemal Heavy Chain 8	a heavy chain of an axonemal dynein involved in sperm and respiratory cilia motility.
15:54630732-Ins		UNC13C	intron_variant	Other	0.16	18.79	27397079.00	3.69E-02	Overall p=0.00152	Race category, TMB, MSI, CIMP, C	Unc-13 Homolog C	May play a role in vesicle maturation during exocytosis as a target of the diacylglycerol second messenger pathway.
9:38569472-Del	rs112902043				0.16	18.79	27397079.00	3.69E-02				
18:61261763-Del		SERPINB13	intron_variant	Other	0.16	18.79	27397079.00	3.69E-02	no	Race category, TMB, MSI,	Serpin Family B Member 13	inhibits the activity of cathepsin K and is itself transcriptionally repressed by RUNX1.
5:31409066-Ins		DROSHA	intron_variant	Other	0.14	18.77	27397079.00	3.74E-02	no	MSI, race category, TMB, CIMP	Drosha Ribonuclease III	Ribonuclease III double-stranded (ds) RNA-specific endoribonuclease that is involved in the initial step of microRNA (miRNA) biogenesis.
18:2726350-Ins		SMCHD1	intron_variant	Other	0.14	18.77	27397079.00	3.74E-02	no	MSI, race category, TMB, CIMP	Structural Maintenance Of Chromosom	Non-canonical member of the structural maintenance of chromosomes (SMC) protein family that plays a key role in epigenetic silencing by regulating chromatin architecture
14:64449254-Del	rs199597838	SYNE2	intron_variant	Other	0.14	18.77	27397079.00	3.74E-02	no (prog free p=0.0920)	MSI, race category, TMB, CIMP, CI	Spectrin Repeat Containing Nuclear Env	a nuclear outer membrane protein that binds cytoplasmic F-actin. This binding tethers the nucleus to the cytoskeleton and aids in the maintenance of the structural integrity of the nucleus.
8:106815856-SNV	rs6991211	ZFPM2,ZFPM2-AS1	3_prime_UTR_variant	Other	0.33	18.66	23726566.41	4.08E-02				
12:131451185-Ins		ADGRD1	intron_variant	Other	0.11	19.05	33554432.00	4.09E-02	Overall p=0.0460	TMB, MSI, CIMP,	Adhesion G Protein-Coupled Receptor D	Orphan receptor of G-protein family, Has protumorigenic function especially in glioblastoma
9:5068896-Ins		JAK2	intron_variant	Other	0.11	19.05	33554432.00	4.09E-02	no	TMB, race category, MSI, sex, CIM	Janus Kinase 2	Non-receptor tyrosine kinase involved in various processes such as cell growth, development, differentiation or histone modifications.
17:12844580-Ins		ARHGAP44	intron_variant	Other	0.13	19.07	33554432.00	4.18E-02	no	Race category, TMB, MSI, CIMP, m	Rho GTPase Activating Protein 44	GTPase-activating protein (GAP) that stimulates the GTPase activity of Rho-type GTPases. Thereby, controls Rho-type GTPases cycling between their active GTP-bound and inactive GDP-bound states. Acts as a GAP at least for CDC42 and RAC1
15:74327359-Ins		PML	3_prime_UTR_variant	Other	0.17	19.08	33554432.00	4.33E-02	no	MSI, race category, TMB,	PML Nuclear Body Scaffold	localizes to nuclear bodies where it functions as a transcription factor and tumor suppressor
2:31606871-Del		XDH	intron_variant	Other	0.17	19.08	33554432.00	4.33E-02	Dis specific p=0.0286	MSI, race category, TMB, CIMP	Xanthine Dehydrogenase	Key enzyme in purine degradation.Contributes to the generation of reactive oxygen species
5:94749904-Ins	rs11418250	FAM81B	intron_variant	Other	0.14	18.75	27397079.00	4.47E-02	no	TMB, MSI	Family With Sequence Similarity 81 Men	Located in nucleus.
5:146793053-Ins		DPYSL3	intron_variant	Other	0.15	18.72	27397079.00	4.50E-02	no	TMB, MSI, CIMP,	Dihydropyrimidinase Like 3	Necessary for signaling by class 3 semaphorins and subsequent remodeling of the cytoskeleton.
6:52764660-Ins		GSTA3	intron_variant	Other	0.15	18.72	27397079.00	4.50E-02	no	Race category, TMB	Glutathione S-Transferase Alpha 3	involved in cellular defense against toxic, carcinogenic, and pharmacologically active electrophilic compounds.
7:21744896-Ins		DNAH11	intron_variant	Other	0.17	18.73	27397079.00	4.54E-02	Dis free p=0.0290	MSI, TMB, race category, CIMP, CI	Dynein Axonemal Heavy Chain 11	Force generating protein of respiratory cilia. Produces force towards the minus ends of microtubules.
18:55321012-Ins		ATP8B1,LOC100505549	intron_variant	Other	0.22	19.12	33554432.00	4.58E-02				
11:8942745-Ins		C11orf16	intron_variant	Other	0.22	19.12	33554432.00	4.58E-02	Overall p=0.0370	TMB	Chromosome 11 Open Reading Frame 1	Uncharacterized Protein C11orf16
1:34049129-Ins		CSMD2	intron_variant	Other	0.22	19.12	33554432.00	4.58E-02	no (dis free p=0.0756)	MSI, race category, TMB, CMS, CIN	CUB And Sushi Multiple Domains 2	thought to be involved in the control of complement cascade of the immune system, may act as a tumor suppressor for colorectal cancer.
12:51072319-Ins		DIP2B	intron_variant	Other	0.22	19.12	33554432.00	4.58E-02	no	Race category, TMB, MSI, CMS, CI	Disco Interacting Protein 2 Homolog B	Negatively regulates axonal outgrowth and is essential for normal synaptic transmission.
4:44712821-Ins		GNPDA2	intron_variant	Other	0.22	19.12	33554432.00	4.58E-02	no	TMB, MSI	Glucosamine-6-Phosphate Deaminase 2	catalyzes the reversible reaction converting D-glucosamine-6-phosphate into D-fructose-6-phosphate and ammonium. associated with influencing body mass index and susceptibility to obesity.
14:21622974-Ins		OR5AU1	downstream_gene_variant	Unknown	0.22	19.12	33554432.00	4.58E-02	no	Race category, MSI, TMB,	Olfactory Receptor Family 5 Subfamily A	Odorant receptor.
12:57494010-Del		STAT6	intron_variant	Other	0.22	19.12	33554432.00	4.58E-02	Prog free p=0.0143, ove	Race category, TMB, MSI, CMS, CI	Signal Transducer And Activator Of Tran	Carries out a dual function: signal transduction and activation of transcription. Involved in IL4/interleukin-4- and IL3/interleukin-3-mediated signaling.
6:132199865-Ins		ENPP1	intron_variant	Other	0.12	18.84	23726566.41	4.72E-02	no	Race category, TMB	Ectonucleotide Pyrophosphatase/Phosp	Nucleotide pyrophosphatase that generates diphosphate (PPi) and functions in bone mineralization and soft tissue calcification by regulating pyrophosphate levels
6:116431924-Ins		NT5DC1	intron_variant	Other	0.12	19.01	33554432.00	4.72E-02	no	Race category, TMB, MSI,	5'-Nucleotidase Domain Containing 1	exact function is not known, belongs to the 5'(3')-deoxyribonucleotidase family
5:177638835-Ins		PHYKPL	intron_variant	Other	0.12	19.01	33554432.00	4.72E-02	no	Race category, TMB, MSI, CIMP	5-Phosphohydroxy-L-Lysine Phospho-Ly	a mitochondrial enzyme that catalyzes the conversion of 5-phosphonooxy-L-lysine to ammonia, inorganic phosphate, and 2-aminoadipate semialdehyde.
15:101827076-Ins		SNRPA1	intron_variant	Other	0.12	19.01	33554432.00	4.72E-02	no	mut count	Small Nuclear Ribonucleoprotein Polype	Involved in pre-mRNA splicing as component of the spliceosome
17:17249950-Ins		NT5M	intron_variant	Other	0.25	18.77	27397079.00	4.73E-02	Overall p=0.0185	Race category, MSI	5',3'-Nucleotidase, Mitochondrial	Dephosphorylates specifically the 5' and 2'(3')-phosphates of uracil and thymine deoxyribonucleotides, and so protects mitochondrial DNA replication from excess dTTP.
7:21781417-Ins-2		DNAH11	intron_variant	Other	0.25	18.77	27397079.00	4.73E-02	see line 162			
4:77065491-Del	rs77895217	NUP54	intron_variant	Other	0.25	18.77	27397079.00	4.73E-02	no	TMB, MSI,	Nucleoporin 54	Component of the nuclear pore complex, a complex required for the trafficking across the nuclear membrane.
17:19314860-Ins		RNF112	intron_variant	Other	0.25	18.77	27397079.00	4.73E-02	no	MSI, TMB, race category	Ring Finger Protein 112	E3 ubiquitin-protein ligase that plays an important role in neuronal differentiation, including neurogenesis and gliogenesis, during brain development.
2:241514213-Del		RNPEPL1	intron_variant	Other	0.25	18.77	27397079.00	4.73E-02	no	MSI, race category, TMB, CMS, CIN	Arginyl Aminopeptidase Like 1	Broad specificity aminopeptidase which preferentially hydrolyzes an N-terminal methionine, citrulline or glutamine.
2:30410171-Ins		SNORA10B	upstream_gene_variant	Unknown	0.25	18.77	27397079.00	4.73E-02	ND not enough sample	ND not enough sample number	Small Nucleolar RNA, H/ACA Box 10B	Small nucleolar RNA SNORA64/SNORA10 family
16:68729948-Ins		CDH3	intron_variant	Other	0.30	18.80	27397079.00	4.83E-02	no	Race category, MSI, TMB,	Cadherin 3	calcium-dependent cell-cell adhesion protein
1:156531520-Ins-2		IQGAP3	intron_variant	Other	0.30	18.80	27397079.00	4.83E-02	Overall p=0.0433	MSI, race category, TMB, CIMP, CI	IQ Motif Containing GTPase Activating P	Predicted to be involved in regulation of actin cytoskeleton organization.
15:72155046-Ins		MYO9A	intron_variant	Other	0.30	18.80	27397079.00	4.83E-02	no	MSI, race category, TMB, CIMP,	Myosin IXA	An unconventional myosin, serving in intracellular movements. Regulates Rho by stimulating it's GTPase activity in neurons.
7:103767469-Del		ORC5	intron_variant	Other	0.30	18.80	27397079.00	4.83E-02	no	MSI, race category, TMB, CIMP	Origin Recognition Complex Subunit 5	Component of the origin recognition complex (ORC) that binds origins of replication.
12:104131673-Ins		STAB2	intron_variant	Other	0.30	18.80	27397079.00	4.83E-02	Dis free p=0.003116, ov	MSI, race category, TMB, CMS1,	Stabilin 2	a large, transmembrane receptor protein which may function in angiogenesis, lymphocyte homing, cell adhesion, or receptor scavenging.
6:109796433-Ins-2		ZBTB24	intron_variant	Other	0.30	18.80	27397079.00	4.83E-02	Overall p=0.007818	Race category, TMB, MSI	Zinc Finger And BTB Domain Containing	May be involved in BMP2-induced transcription.
11:73669218-Ins		DNAJB13	intron_variant	Other	0.14	19.01	33554432.00	4.99E-02	no	Race category, TMB, MSI,	DnaJ Heat Shock Protein Family (Hsp40)	Functions as part of axonemal radial spoke complexes that play an important part in the motility of sperm and cilia.
2:65481099-Del		ACTR2	intron_variant	Other	0.14	19.01	33554432.00	4.99E-02	no	Race category, TMB	Actin Related Protein 2	a major constituent of the ARP2/3 complex that is essential to cell shape and motility through lamellipodial actin assembly and protrusion
2:30975767-Ins		CAPN13	intron_variant	Other	0.14	19.01	33554432.00	4.99E-02	no	Race category, TMB, MSI,	Calpain 13	a member of the calpain large subunit family. A calcium-activated neutral protease
15:65871598-Ins		INTS14	3_prime_UTR_variant	Other	0.14	19.01	33554432.00	4.99E-02	no	Race category, TMB	Integrator Complex Subunit 14	Probable component of the Integrator (INT) complex, a complex involved in the small nuclear RNAs (snRNA) U1 and U2 transcription and in their 3'-box-dependent processing.
18:77623561-Del	rs1979306039	KCNG2	upstream_gene_variant	Unknown	0.14	19.01	33554432.00	4.99E-02	no (prog free p=0.0865)	TMB, MSI, ICR high	Potassium Voltage-Gated Channel Modi	Potassium channel subunit
1:174987485-Ins		MRPS14	intron_variant	Other	0.14	19.01	33554432.00	4.99E-02	Dis free p=0.0130	mut count	Mitochondrial Ribosomal Protein S14	a 28S subunit protein of mammalian mitochondrial ribosome
14:75563692-Ins		NEK9	intron_variant	Other	0.14	19.01	33554432.00	4.99E-02	no	Race category, TMB, MSI	NIMA Related Kinase 9	Pleiotropic regulator of mitotic progression, participating in the control of spindle dynamics and chromosome separation
5:72157517-Ins		TNPO1	intron_variant	Other	0.14	19.01	33554432.00	4.99E-02	no	Race category, MSI, TMB,	Transportin 1	the beta subunit of the karyopherin receptor complex which interacts with nuclear localization signals to target nuclear proteins to the nucleus.
10:81063685-Ins		ZMIZ1	intron_variant	Other	0.14	19.01	33554432.00	4.99E-02	no (overall p=0.0717)	MSI, TMB	Zinc Finger MIZ-Type Containing 1	regulates the activity of various transcription factors, including the androgen receptor, Smad3/4, and p53
15:22482683-Ins					0.14	18.76	23726566.41	4.99E-02				

Supplementary Table 2a Black/AA

Predictor	Identifier	Gene Names	Sequence Ontology	Effect	MAF	Beta	log2FoldChar	P-Value	survival influence (p<0.05)?	other notables p<0.05	full gene name	protein and function	C publicati	cancer pub	publication#
1:213178828-Ins		ANGEL2	intron_variant	Other	0.18	18.64	0.00131132	1.11E-02	Dis free p=0.0263	Race category, MSI, TMB, CIMP	Angel Homolog 2	Involved in 3'-UTR-mediated mRNA stabilization and negative regulation of mitotic cell cycle	0	1	3
1:92573371-Ins		BTBD8	intron_variant	Other	0.18	19.12	0.17321944	1.22E-02	Overall p=0.0274	Race category, TMB, Micro score	BTB Domain Containing 8	Involved in clathrin-mediated endocytosis at the synapse.	0	1	4
10:88556184-Ins		BMPR1A	intron_variant	Other	0.31	18.75	0.01988386	1.42E-02	Overall p=0.0238	TMB, MSI, BMI	Bone Morphogenetic Protein Receptor Type 1A	On ligand binding, forms a receptor complex consisting of two type II and two type I transn	59	328	901
11:117886314-Ins-2		SMIM35	intron_variant	Other	0.16	19.05	-0.00391149	1.44E-02	Prog free p=0.009	Race category	Small Integral Membrane Protein 35	Predicted to be integral component of membrane	0	0	0
3:57269383-Ins		APPL1	intron_variant	Other	0.22	19.34	0.03505803	1.68E-02	Overall p=0.0396	Race category, TMB, MSI, CMS1 and 3,	Adaptor Protein, Phosphotyrosine Interacting With PH Domain And Leucine Zipper 1	Multifunctional adapter protein that binds to various membrane receptors, nuclear factors ar	1	66	302
17:37579508-Ins		MED1	intron_variant	Other	0.17	18.83	-0.0674786	1.76E-02	Overall p=0.028	TMB, MSI,	Mediator Complex Subunit 1	Component of the Mediator complex, a coactivator involved in the regulated transcription o	19	315	1108
5:71521792-Ins		MRPS27	intron_variant	Other	0.17	19.01	0.07325817	1.76E-02	Prog free p=0.0235	Race category, TMB, MSI	Mitochondrial Ribosomal Protein S27	RNA-binding component of the mitochondrial small ribosomal subunit (mt-SSU) that plays	0	2	17
16:18839297-Ins		SMG1	intron_variant	Other	0.17	19.01	0.04762202	1.76E-02	Overall p=0.0254	Race category, TMB, MSI, CMS1 and 3,	SMG1 Nonsense Mediated MRNA Decay Associated PI3K Related Kinase	Serine/threonine protein kinase involved in both mRNA surveillance and genotoxic stress res	1	73	220
19:37101428-Ins		ZNF382	intron_variant	Other	0.15	19.41	0.06689293	1.78E-02	Prog free p=0.0439	Race category, TMB, MSI,	Zinc Finger Protein 382	Functions as a sequence-specific transcriptional repressor.	1	21	27
1:175129682-Ins		KIAA0040	3_prime_UTR_variant	Other	0.20	19.01	-0.0335749	1.96E-02	Dis free p=0.0022		KIAA0040	Uncharacterized Protein KIAA0040, Predicted to be integral component of membrane.	0	2	14
1:36068784-Ins-2		PSMB2	3_prime_UTR_variant	Other	0.20	19.01	0.15878608	1.96E-02	Overall p=0.0253	MSI, TMB, CMS	Proteasome 20S Subunit Beta 2	Non-catalytic component of the 20S core proteasome complex	1	22	54
17:7574340-Ins		TP53	intron_variant	Other	0.18	18.95	-0.04676849	2.18E-02	Overall p=0.0000015	MSI, race category, CMS, CIMP,	Tumor Protein P53	a tumor suppressor protein containing transcriptional activation, DNA binding, and oligome	1140	26626	30380
20:62378693-Ins	rs1199903552	ZBTB46	intron_variant	Other	0.18	18.95	-0.03888236	2.18E-02	Overall p=0.0276	MSI, CIMP	Zinc Finger And BTB Domain Containing 46	Predicted to be involved in regulation of transcription by RNA polymerase II.	1	42	94
1:9005719-Ins		CA6	upstream_gene_variant	Unknown	0.25	19.46	0.03497811	2.29E-02	Dis free p=0.0478	TMB	Carbonic Anhydrase 6	Reversible hydration of carbon dioxide.found only in salivary glands and saliva.	1	31	246
9:74828655-Ins		GDA	intron_variant	Other	0.25	19.46	0.10055252	2.29E-02	Dis specific p=0.038	Race category, MSI, TMB	Guanine Deaminase	Catalyzes the hydrolytic deamination of guanine, producing xanthine and ammonia.	3	117	739
3:164704708-Ins		SI	intron_variant	Other	0.25	19.46	0.07352369	2.29E-02	Dis free p=0.0365	Race category, TMB, CIMP,	Sucrase-Isomaltase	essential for the digestion of dietary carbohydrates including starch, sucrose and isomaltose	85	131	1199
17:38142763-Ins		PSMD3	intron_variant	Other	0.16	18.92	-0.01398378	2.37E-02	Dis specific p=0.0379	MSI, TMB,	Proteasome 26S Subunit, Non-ATPase 3	Component of the 26S proteasome,	0	22	46
12:122818749-Del		CLIP1	intron_variant	Other	0.21	18.94	0.10104381	2.55E-02	Dis free p=0.008663	Race category, TMB, MSI,	CAP-Gly Domain Containing Linker Protein 1	Binds to the plus end of microtubules and regulates the dynamics of the microtubule cytosk	0	29	56
19:55589357-Ins		EPS8L1	intron_variant	Other	0.21	18.94	0.08528299	2.55E-02	Prog free p=0.0492	Race category, MSI, TMB, CMS, CIMP	EPS8 Signaling Adaptor L1	Stimulates guanine exchange activity of SOS1. May play a role in membrane ruffling and ren	0	2	14
3:4741740-Del		ITPR1	intron_variant	Other	0.21	18.94	-0.0098828	2.55E-02	Dis free p=0.0301	MSI, race catgeory,TMB,	Inositol 1,4,5-Trisphosphate Receptor Type 1	an intracellular receptor for inositol 1,4,5-trisphosphate. Upon stimulation, this receptor med	2	138	912
19:50496360-Del		VRK3	intron_variant	Other	0.21	18.94	0.05629575	2.55E-02	Overall p=0.00992	Race category, MSI, TMB	VRK Serine/Threonine Kinase 3	Inactive kinase that suppresses ERK activity by promoting phosphatase activity of DUSP3 wh	1	6	24
17:3840695-Del	rs34866939	ATP2A3	intron_variant	Other	0.30	1.15	-0.16563941	2.64E-02	Overall p=0.0110	TMB, MSI, CIMP, CMS	ATPase Sarcoplasmic/Endoplasmic Reticulum Ca2+ Transporting 3	catalyzes the hydrolysis of ATP coupled with the translocation of calcium from the cytosol to	12	38	120
9:79319596-Ins		PRUNE2	intron_variant	Other	0.19	18.89	0.02486063	2.75E-02	Dis free p=0.0274	MSI, race category, TMB, CIMP, CMS1, ICR high	Prune Homolog 2 With BCH Domain	May play an important role in regulating differentiation, survival and aggressiveness of the t	0	39	58
16:67318608-Ins		PLEKHG4	splice_region_variant	Other	0.14	18.82	-0.02648747	3.13E-02	Overall p=0.001095	Race category, TMB, MSI, ICR high, micro score	Pleckstrin Homology And RhoGEF Domain Containing G4	can function as a guanine nucleotide exchange factor (GEF) and may play a role in intracellula	0	2	29
1:156087782-Del		LMNA	intron_variant	Other	0.27	18.92	-0.13185368	3.25E-02	Dis free p=0.0236	Race category, MSI, TMB, CIMP, CMS	Lamin A/C	part of the nuclear lamina	14	278	2191
5:58272398-Ins		PDE4D	intron_variant	Other	0.27	18.92	0.07264981	3.25E-02	Overall p=0.0258	TMB	Phosphodiesterase 4D	has 3',5'-cyclic-AMP phosphodiesterase activity and degrades cAMP, which acts as a signal t	1	109	826
1:211923219-Ins		LPGAT1	3_prime_UTR_variant	Other	0.15	19.15	0.1328728	3.53E-02	Dis free p=0.0159	MSI	Lysophosphatidylglycerol Acyltransferase 1	Lysophospholipid acyltransferase involved in fatty acyl chain remodeling of glycerophospho	0	4	27
15:54630732-Ins		UNC13C	intron_variant	Other	0.16	18.79	0.05769984	3.69E-02	Overall p=0.00152	Race category, TMB, MSI, CIMP, CMS	Unc-13 Homolog C	May play a role in vesicle maturation during exocytosis as a target of the diacylglycerol seco	1	9	38
12:131451185-Ins		ADGRD1	intron_variant	Other	0.11	19.05	-0.00968227	4.09E-02	Overall p=0.0460	TMB, MSI, CIMP,	Adhesion G Protein-Coupled Receptor D1	Orphan receptor of G-protein family, Has protumorigenic function especially in glioblastoma	0	10	27
2:31606871-Del		XDH	intron_variant	Other	0.17	19.08	0.00091117	4.33E-02	Dis specific p=0.0286	MSI, race category, TMB, CIMP	Xanthine Dehydrogenase	Key enzyme in purine degradation.Contributes to the generation of reactive oxygen species	3	57	815
7:21744896-Ins		DNAH11	intron_variant	Other	0.17	18.73	0.13863206	4.54E-02	Dis free p=0.0290	MSI, TMB, race category, CIMP, CMS,	Dynein Axonemal Heavy Chain 11	Force generating protein of respiratory cilia. Produces force towards the minus ends of micr	1	17	124
11:8942745-Ins		C11orf16	intron_variant	Other	0.22	19.12	-0.13673763	4.58E-02	Overall p=0.0370	TMB	Chromosome 11 Open Reading Frame 16	Uncharacterized Protein C11orf16	0	0	0
12:57494010-Del		STAT6	intron_variant	Other	0.22	19.12	-0.0708193	4.58E-02	Prog free p=0.0143, overall p=0.0486	Race category, TMB, MSI, CMS, CIMP	Signal Transducer And Activator Of Transcription 6	Carries out a dual function: signal transduction and activation of transcription. Involved in IL	50	1242	4337
17:17249950-Ins		NT5M	intron_variant	Other	0.25	18.77	-0.02467933	4.73E-02	Overall p=0.0185	Race category, MSI	5',3'-Nucleotidase, Mitochondrial	Dephosphorylates specifically the 5' and 2'(3')-phosphates of uracil and thymine deoxyribon	0	0	8
1:156531520-Ins-2		IQGAP3	intron_variant	Other	0.30	18.80	-0.1142018	4.83E-02	Overall p=0.0433	MSI, race category, TMB, CIMP, CMS	IQ Motif Containing GTPase Activating Protein 3	Predicted to be involved in regulation of actin cytoskeleton organization.	3	64	89
12:104131673-Ins		STAB2	intron_variant	Other	0.30	18.80	0.07340242	4.83E-02	Dis free p=0.003116, overall p=0.0215	MSI, race category, TMB, CMS1,	Stabilin 2	a large, transmembrane receptor protein which may function in angiogenesis, lymphocyte h	2	42	155
6:109796433-Ins-2		ZBTB24	intron_variant	Other	0.30	18.80	0.00222211	4.83E-02	Overall p=0.007818	Race category, TMB, MSI	Zinc Finger And BTB Domain Containing 24	May be involved in BMP2-induced transcription.	0	10	48
1:174987485-Ins		MRPS14	intron_variant	Other	0.14	19.01	0.18557195	4.99E-02	Dis free p=0.0130	mut count	Mitochondrial Ribosomal Protein S14	a 28S subunit protein of mammalian mitochondrial ribosome	0	0	3

Supplementary Table 2b Black/AA SCGs

Predictor	Identifier	Gene Names	Sequence Ontology	Effect	MAF	Beta	SE	P-Value	survival influence (p<0.0	other notables p<0.05	full gene name	protein and function
6:74304607-SNV	rs3734517	SLC17A5	3_prime_UTR_variant	Other	0.15	20.21	23726566.41	2.66E-05	no	Race category, TMB, MSI,	Solute Carrier Family 17 Member 5	a membrane transporter that exports free sialic acids
2:108608648-SNV	rs1013940	SLC5A7	missense_variant	Missense	0.15	20.21	23726566.41	2.66E-05	no	Race category, mutation count,	Solute Carrier Family 5 Member 7	a sodium ion- and chloride ion-dependent high-affinity transporter
11:20065673-SNV	rs3802799	NAV2,NAV2-AS2	missense_variant	Missense	0.19	2.22	0.76	3.59E-04	no	MSI, TMB, race category, CMS1	NAV2 Antisense RNA 2	an RNA Gene
19:54782919-SNV	rs386056	LILRB2	missense_variant	Missense	0.26	1.93	0.66	4.56E-04	no	Race category, MSI, TMB	Leukocyte Immunoglobulin Like Rece	control inflammatory responses and cytotoxicity
12:58129303-Ins	rs148173870	AGAP2	intron_variant	Other	0.21	2.14	0.76	6.17E-04	no (overall p=0.0625)	MSI, Race category, TMB, CIMP	ArfGAP With GTPase Domain, Ankyrin	promotes cancer cell invasion
13:95056768-SNV	rs1951797	GPC6	3_prime_UTR_variant	Other	0.18	1.87	0.67	1.19E-03	no	mutation count, MSI, race categ	Glypican 6	a putative cell surface coreceptor for growth factors, extracellular matrix proteins, proteases and anti-proteases
1:11856378-SNV	rs1801133	MTHFR	missense_variant	Missense	0.18	1.87	0.67	1.19E-03	no (overall p=0.0581)	Race category, TMB, MSI,	Methylenetetrahydrofolate Reductas	catalyzes the conversion of 5,10-methylenetetrahydrofolate to 5-methyltetrahydrofolate, a co-substrate for homocysteine remethylation to methionine
5:140870516-SNV	rs2074912	CDHGA11,PCDHGA	missense_variant	Missense	0.18	1.87	0.67	1.19E-03	no	MSI, TMB, race category, CIMP	Protocadherin Gamma-A1	Protocadherin gene cluster
12:9013755-SNV	rs1860967	A2ML1	missense_variant	Missense	0.22	1.84	0.67	1.41E-03	no	Race category, TMB, MSI,	Alpha-2-Macroglobulin Like 1	an N-glycosylated monomeric protein that acts as an inhibitor of several proteases
20:48894756-SNV	rs3088220	PELATON	missense_variant	Missense	0.19	1.84	0.67	1.41E-03	no	Race category, TMB	Plaque Enriched LncRNA In Atheroscl	an RNA Gene
12:51864134-Ins		SLC4A8	intron_variant	Other	0.08	20.21	33554432.00	1.47E-03	no (prog free p=0.0743)	Race category, TMB, MSI, CIMP	Solute Carrier Family 4 Member 8	to transport sodium and bicarbonate ions across the cell membrane
12:40808672-SNV	rs4768252	MUC19	missense_variant	Missense	0.09	19.91	23726566.41	1.77E-03	no	Race category, TMB, MSI, CMS	Mucin 19, Oligomeric	responsible for the gel-like properties of mucus
11:102567038-Ins		MMP27	intron_variant	Other	0.09	19.97	23726566.41	1.96E-03	no	TMB, MSI, Ethnicity category,M	Matrix Metallopeptidase 27	involved in the breakdown of extracellular matrix
11:102567039-Ins		MMP27	intron_variant	Other	0.09	19.97	23726566.41	1.96E-03				
20:21329982-Ins		XRN2	intron_variant	Other	0.10	20.10	33554432.00	2.18E-03	no (overall p=0.0985)	TMB, MSI, Race category	5'-3' Exoribonuclease 2	promotes transcription termination at cotranscriptional cleavage sites
12:31648826-SNV	rs4930979	DENND5B	missense_variant	Missense	0.21	1.76	0.67	2.42E-03	no(dis free p=0.0859)	Race category, TMB, MSI,	DENN Domain Containing 5B	Enables guanyl-nucleotide exchange factor activity
20:3275049-Ins		DNAAF9	intron_variant	Other	0.10	20.08	33554432.00	2.44E-03	no	Race category, TMB, MSI,	Dynein Axonemal Assembly Factor 9	an uncharacterized protein with a C-terminal coiled-coil region
12:992032-Ins		WNK1	intron_variant	Other	0.10	20.08	33554432.00	2.44E-03	no	TMB, race category, MSI, CMS	WNK Lysine Deficient Protein Kinase	serine/threonine protein kinase, regulator of blood pressure by controlling the transport of sodium and chloride ions
5:112047955-Ins	rs72545070	APC	intron_variant	Other	0.32	1.56	0.61	2.86E-03	overall 0.0231	MSI, Race category, TMB, CIMP	Adenomatosis Polyposis Coli	a tumor suppressor protein that acts as an antagonist of the Wnt signaling pathway
2:38296890-SNV	rs2855658	CYP1B1	3_prime_UTR_variant	Other	0.32	1.56	0.61	2.86E-03	no	Race category, TMB, MSI,	Cytochrome P450 Family 1 Subfamily	a member of the cytochrome P450 superfamily of enzymes
10:26987109-Ins		PDSS1	intron_variant	Other	0.32	1.56	0.61	2.86E-03	no	Race category, TMB, MSI,	Decaprenyl Diphosphate Synthase Sul	coenzyme Q biosynthesis
1:235826383-Del	rs36014994	LYST	splice_region_variant	Other	0.32	1.56	0.61	2.86E-03	no	MSI,TMB, Race category, CMS1	Lysosomal Trafficking Regulator	regulates intracellular protein trafficking in endosomes
9:71940190-Del	rs145867264	FAM189A2	intron_variant	Other	0.22	1.73	0.67	2.95E-03	no (overall p=0.09)	Race category, mutation count,	Endosomal Transmembrane Epsin Int	Predicted to be integral component of membrane
11:51516036-SNV	rs11246608	OR4C46	missense_variant	Missense	0.26	3.91	1.32	3.00E-03	dis specific 0.0457	MSI, TMB, Race catgory,	Olfactory Receptor Family 4 Subfamil	Odorant receptor. G-protein coupled receptor
11:94150359-Ins		MRE11	3_prime_UTR_variant	Other	0.11	20.01	33554432.00	3.11E-03	no	MSI, TMB, Race catgory, sex,	MRE11 Homolog, Double Strand Brea	involved in homologous recombination, telomere length maintenance, and DNA double-strand break repair
21:41239282-Ins		PCP4	upstream_gene_variant	Unknown	0.11	20.01	33554432.00	3.11E-03	no	Race category	Purkinje Cell Protein 4	Involved in calmodulin dependent kinase signaling pathway
16:53504181-Del		RBL2	intron_variant	Other	0.11	20.01	33554432.00	3.11E-03	no (overall p=0.0776)	MSI, Race category, TMB, CIMP	RB Transcriptional Corepressor Like 2	Key regulator of entry into cell division. Directly involved in heterochromatin formation
10:112579515-Ins		RBM20	intron_variant	Other	0.11	20.01	33554432.00	3.11E-03	no (dis free p=0.066)	Race category, TMB, MSI, CMS	RNA Binding Motif Protein 20	binds RNA and regulates splicing
20:35827029-Ins	rs146714866	RPN2	intron_variant	Other	0.11	20.01	33554432.00	3.11E-03	no	MSI, TMB	Ribophorin II	Subunit of the oligosaccharyl transferase (OST) complex
4:48492769-SNV	rs74929644	ZAR1	missense_variant	Missense	0.33	1.53	0.61	3.52E-03	no (overall p=0.0511)	Race category, TMB, MSI, CMS:	Zygote Arrest 1	mRNA-binding protein that mediates formation of MARDO (mitochondria-associated ribonucleoprotein domain), a membraneless compartment that stores maternal mRNAs in oocytes
2:207655197-Ins		FASTKD2	intron_variant	Other	0.12	19.98	33554432.00	3.56E-03	no	Race category, TMB	FAST Kinase Domains 2	assembly of the mitochondrial large ribosomal subunit, may play a role in mitochondrial apoptosis
9:130119762-Del		GARNL3	intron_variant	Other	0.12	19.98	33554432.00	3.56E-03	no	Race category, TMB, MSI	GTPase Activating Rap/RanGAP Doma	Predicted to be involved in activation of GTPase activity
4:2934734-Ins		MFSD10	intron_variant	Other	0.12	19.98	33554432.00	3.56E-03	no	TMB, MSI	Major Facilitator Superfamily Domair	Probable organic anion transporter which may serve as a transporter for some non-steroidal anti-inflammatory drugs (NSAIDs) as well as other organic anions
2:55899044-Ins		PNPT1	intron_variant	Other	0.12	19.98	33554432.00	3.56E-03	no	Race category, TMB	Polyribonucleotide Nucleotidyltransfe	RNA-binding protein implicated in numerous RNA metabolic processes
11:51516000-SNV	rs11246607	OR4C46	missense_variant	Missense	0.26	1.62	0.64	3.81E-03	(see line 25)			
1:153926916-Del		CRTC2	intron_variant	Other	0.13	19.94	33554432.00	4.10E-03	Dis free 0.0480	MSI, TMB	CREB Regulated Transcription Coacti	transcriptional coactivator for CREB1
19:50365614-Ins		PNKP	intron_variant	Other	0.13	19.94	33554432.00	4.10E-03	no	Race category, MSI, TMB, CMS:	Polynucleotide Kinase 3'-Phosphatas	repair of DNA damage, functioning as part of both the non-homologous end-joining (NHEJ) and base excision repair (BER) pathways
11:64700102-Ins		PPP2R5B	intron_variant	Other	0.13	19.94	33554432.00	4.10E-03	no (Overall p=0.0603)	Race category, TMB, MSI	Protein Phosphatase 2 Regulatory Sul	phosphatase 2A regulatory subunit B
7:30795331-SNV	rs2302340	NMT,INMT-MINDY	missense_variant	Missense	0.21	1.50	0.57	4.54E-03	no	Race category, MSI	INMT-MINDY4 Readthrough (NMD Ca	read-through transcription between the INMT and FAM188B
6:49398115-SNV	rs9381784	MMUT	3_prime_UTR_variant	Other	0.21	1.50	0.57	4.54E-03	no	Race category, TMB, MSI	Methylmalonyl-CoA Mutase	a vitamin B12-dependent enzyme which catalyzes the isomerization of methylmalonyl-CoA to succinyl-CoA
19:7599060-SNV	rs560849	PNPLA6	5_prime_UTR_variant	Other	0.21	1.50	0.57	4.54E-03	no	Race category, TMB, MSI, CMS:	Patatin Like Phospholipase Domain C	a phospholipase that deacetylates intracellular phosphatidylcholine to produce glycerophosphocholine
17:28811150-Ins		GOSR1	intron_variant	Other	0.13	19.65	23726566.41	4.78E-03	Dis free 0.004, oveall 0.0	Race category, TMB	Golgi SNAP Receptor Complex Memb	an essential component of the Golgi SNAP receptor (SNARE) complex
2:113590554-Del		IL1B	intron_variant	Other	0.13	19.65	23726566.41	4.78E-03	no	Race category, TMB, MSI	Interleukin 1 Beta	pro-inflammatory cytokine
18:42646635-Del-2	rs34125334	SETBP1	3_prime_UTR_variant	Other	0.21	1.87	0.78	5.49E-03	overall 0.0099	TMB, MSI, CMS1, ICR high, race	SET Binding Protein 1	bind the SET nuclear oncogene which is involved in DNA replication
15:75651027-Ins		MAN2C1	intron_variant	Other	0.36	1.42	0.60	6.14E-03	no	MSI, Race category, TMB, CIMP	Mannosidase Alpha Class 2C Member	Predicted to be involved in oligosaccharide catabolic process
15:75651030-Ins		MAN2C1	intron_variant	Other	0.36	1.42	0.60	6.14E-03				
12:56551340-Ins	rs28365930	MYL6B	intron_variant	Other	0.23	1.45	0.57	6.35E-03	no	Race category, MSI, TMB, sex,	Myosin Light Chain 6B	Regulatory light chain of myosin
8:110453139-Del		PKHD1L1	intron_variant	Other	0.08	19.61	33554432.00	7.26E-03	no (Overall p=0.00567)	MSI, TMB, Race category, CIMP	Polycystic Kidney And Hepatic Disea	Predicted to act upstream of or within sensory perception of sound
12:69759552-Del	rs11286051	YEATS4	intron_variant	Other	0.30	1.50	0.64	7.96E-03	no	Race category, TMB, MSI	YEATS Domain Containing 4	Chromatin reader component of the NuA4 histone acetyltransferase (HAT) complex, a complex involved in transcriptional activation of select genes
12:96282136-Ins		CCDC38	intron_variant	Other	0.11	19.63	33554432.00	8.09E-03	no	Race category, TMB, MSI, CIMF	Coiled-Coil Domain Containing 38	Located in centrosome.
12:10205175-Ins		CLEC9A	intron_variant	Other	0.11	19.63	33554432.00	8.09E-03	no	Race category, TMB	C-Type Lectin Domain Containing 9A	an endocytic receptor on a small subset of myeloid cells specialized for the uptake and processing of material from dead cells.
12:58204431-Ins	rs3216741	AVIL	intron_variant	Other	0.24	1.79	0.78	8.15E-03	no	MSI, Race category, TMB	Advillin	a member of the gelsolin/villin family of actin regulatory proteins
10:4877835-Ins		AKR1E2	intron_variant	Other	0.30	1.45	0.62	8.39E-03	no	Race category, MSI, TMB	Aldo-Keto Reductase Family 1 Memb	Catalyzes the NADPH-dependent reduction of 1,5-anhydro-D-fructose (AF) to 1,5-anhydro-D-glucitol
2:51256013-SNV	rs62143026	NRXN1	:_premature_start_codon_	Missense	0.07	19.51	33554432.00	9.26E-03	Dis free 0.0256	MSI, TMB, CIMP, Race category	Neurexin 1	Cell surface protein involved in cell-cell-interactions, exocytosis of secretory granules and regulation of signal transmission.
1:145075923-SNV	rs1553349156	PDE4DIP	5_prime_UTR_variant	Other	0.07	19.51	33554432.00	9.26E-03	no	MSI, Race category, TMB,	Phosphodiesterase 4D Interacting Pro	an anchor sequestering components of the cAMP-dependent pathway to Golgi and/or centrosomes
3:68934434-SNV	rs4855535	TAFA4	5_prime_UTR_variant	Other	0.09	19.53	33554432.00	9.78E-03	Dis free 0.00155	Race category, MSI	TAFA Chemokine Like Family Member	postulated to function as brain-specific chemokines or neurokines, that act as regulators of immune and nervous cells
7:18067480-Del	rs35708479	PRPS1L1	upstream_gene_variant	Unknown	0.31	1.42	0.62	9.96E-03	no	Race category, TMB	Phosphoribosyl Pyrophosphate Synth	Catalyzes the synthesis of phosphoribosylpyrophosphate (PRPP) that is essential for nucleotide synthesis.
4:68621586-Del	rs34993359				0.31	1.42	0.62	9.96E-03				
17:14111361-Del	rs397763766	COX10	3_prime_UTR_variant	Other	0.07	19.49	33554432.00	1.01E-02	no (overall p=0.0544)	Race category, MSI, TMB,	Cytochrome C Oxidase Assembly Fact	catalyzes the electron transfer from reduced cytochrome c to oxygen
9:35079974-Ins	rs16935545	FANCG	5_prime_UTR_variant	Other	0.07	19.49	33554432.00	1.01E-02	no	Race category, MSI, TMB,	FA Complementation Group G	DNA repair protein that may operate in a postreplication repair or a cell cycle checkpoint function. May be implicated in interstrand DNA cross-link repair and in the maintenance of normal chromosome stability. Candidate tumor suppressor gene.
8:134308938-Del	rs36215434	NDRG1	intron_variant	Other	0.25	1.75	0.78	1.01E-02	Dis specific 0.00851	mut count	N-Myc Downstream Regulated 1	involved in stress responses, hormone responses, cell growth, and differentiation
3:33186356-SNV	rs1127898	CRTAP	3_prime_UTR_variant	Other	0.14	1.52	0.65	1.07E-02	no (Overall p=0.056)	Race category, TMB	Cartilage Associated Protein	Necessary for efficient 3-hydroxylation of fibrillar collagen prolyl residues.
6:32943980-Ins	rs2066741	BRD2	intron_variant	Other	0.32	1.39	0.62	1.19E-02	no	Race category, TMB, MSI	Bromodomain Containing 2	a transcriptional regulator that belongs to the BET (bromodomains and extra terminal domain) family of proteins
12:6054250-Ins		ANO2	intron_variant	Other	0.10	19.46	33554432.00	1.23E-02	no	Race category, TMB	Anoctamin 2	Calcium-activated chloride channel (CaCC) which may play a role in olfactory signal transduction.
4:54139976-Del	rs150594118	SCFD2	intron_variant	Other	0.14	1.79	0.80	1.54E-02	Overall p=0.004482	Race category, TMB, MSI	Sec1 Family Domain Containing 2	Predicted to be involved in intracellular protein transport and vesicle docking involved in exocytosis.
2:219501328-SNV	rs1055816	PLCD4,ZNF142	3_prime_UTR_variant	Other	0.24	1.45	0.66	1.58E-02				
5:7851181-SNV	rs161874	C5orf49	5_prime_UTR_variant	Other	0.11	19.39	33554432.00	1.58E-02	no	mut count	CFAP90/Cilia And Flagella Associated	Located in ciliary basal body.
15:55881107-SNV	rs1992237	PYGO1	5_prime_UTR_variant	Other	0.27	1.21	0.53	1.66E-02	Prog free p=0.0334	Race category, MSI, TMB, CIMP	Pygopus Family PHD Finger 1	Involved in signal transduction through the Wnt pathway.
10:97366107-SNV	rs8758	ALDH18A1	3_prime_UTR_variant	Other	0.19	1.35	0.59	1.66E-02	Overall p=0.002169	TMB, race category, MSI, CMS1	Aldehyde Dehydrogenase 18 Family M	a member of the aldehyde dehydrogenase family and encodes a bifunctional ATP- and NADPH-dependent mitochondrial enzyme
1:116235183-Del	rs3841008	VANGL1	3_prime_UTR_variant	Other	0.19	1.35	0.59	1.66E-02	no	MSI, race category, TMB	VANGL Planar Cell Polarity Protein 1	may be involved in mediating intestinal trefoil factor induced wound healing in the intestinal mucosa.
3:124196239-Del	rs3214750	KALRN	intron_variant	Other	0.14	1.76	0.80	1.71E-02	no	MSI, race category, TMB, CIMP,	Kalirin RhoGEF Kinase	interacts with the huntingtin-associated protein 1
22:23603475-Ins		BCR	intron_variant	Other	0.05	19.91	33554432.00	2.06E-02	Overall p=0.0473	MSI, race category, TMB,	BCR Activator Of RhoGEF And GTPase	Although the BCR-ABL fusion protein has been extensively studied, the function of the normal BCR gene product is not clear.
2:227915536-Ins		COL4A4	intron_variant	Other	0.05	19.91	33554432.00	2.06E-02	Prog free p=0.0211	Race category, TMB,	Collagen Type IV Alpha 4 Chain	one of the six subunits of type IV collagen
17:26657398-Ins-2		IFT20	intron_variant	Other	0.05	19.91	33554432.00	2.06E-02	no	Race category	Intraflagellar Transport 20	part of a complex involved in trafficking of proteins from the Golgi body, including recycling of immune signalling components
3:156192516-Ins		KCNAB1	splice_region_variant	Other	0.05	19.91	33554432.00	2.06E-02	no	Race category, TMB, MSI,	Potassium Voltage-Gated Channel Su	Cytoplasmic potassium channel subunit that modulates the characteristics of the channel-forming alpha-subunits
11:9874097-Ins		LOC101928008,SBF	intron_variant	Other	0.05	19.91	33554432.00	2.06E-02				
2:44127040-Del		LRPPRC	intron_variant	Other	0.05	19.91	33554432.00	2.06E-02	Prog free p=0.0149	MSI, TMB, race category, CIMP,	Leucine Rich Pentatricopeptide Repea	May play a role in RNA metabolism in both nuclei and mitochondria
1:72400727-Ins		NEGR1	intron_variant	Other	0.05	19.91	33554432.00	2.06E-02	Overall p=0.00346		Neuronal Growth Regulator 1	May function as a trans-neural growth-promoting factor in regenerative axon sprouting in the mammalian brain
17:35549007-Ins		ACACA	intron_variant	Other	0.05	19.97	33554432.00	2.17E-02	no	Race category, TMB, MSI, ICR h	Acetyl-CoA Carboxylase Alpha	Cytosolic enzyme that catalyzes the carboxylation of acetyl-CoA to malonyl-CoA, the first and rate-limiting step of de novo fatty acid biosynthesis
12:57872774-Ins		ARHGAP9	intron_variant	Other	0.05	19.97	33554432.00	2.17E-02	no	Race category, TMB, MSI, micro	Rho GTPase Activating Protein 9	GTPase activator for the Rho-type GTPases by converting them to an inactive GDP-bound state. Has a role in regulating adhesion of hematopoietic cells to the extracellular matrix.
11:83182643-Ins		DLG2	intron_variant	Other	0.05	19.97	33554432.00	2.17E-02	no (prog free p=0.0835)	TMB,MSI, race category	Discs Large MAGUK Scaffold Protein 2	may interact at postsynaptic sites to form a multimeric scaffold for the clustering of receptors, ion channels, and associated signaling proteins.
6:74344923-Ins		SLC17A5	intron_variant	Other	0.05	19.97	33554432.00	2.17E-02	no	Race category, TMB, MSI,	Solute Carrier Family 17 Member 5	a membrane transporter that exports free sialic acids that have been cleaved off of cell surface lipids and proteins from lysosomes.
16:3644746-Ins		SLX4	intron_variant	Other	0.05	19.97	33554432.00	2.17E-02	no (overall p=0.0570)	MSI, race category, TMB, ICR hi	SLX4 Structure-Specific Endonuclease	required for repair of specific types of DNA lesions and critical for cellular responses to replication fork failure
2:214204856-Ins-2		SPAG16	intron_variant	Other	0.05	19.97	33554432.00	2.17E-02	Dis free p=0.0344	TMB, MSI	Sperm Associated Antigen 16	Necessary for sperm flagellar function. Plays a role in motile ciliogenesis.
3:4417933-Ins		SUMF1	intron_variant	Other	0.05	19.97	33554432.00	2.17E-02	no	MSI, race category, TMB,	Sulfatase Modifying Factor 1	an enzyme that catalyzes the hydrolysis of sulfate esters by oxidizing a cysteine residue in the substrate sulfatase to an active site 3-oxoalanine residue,
1:100356478-Ins		AGL	intron_variant	Other	0.05	20.08	47453132.81	2.44E-02	no	MSI, TMB, race category,	Amylo-Alpha-1, 6-Glucosidase, 4-Alph	the glycogen debrancher enzyme which is involved in glycogen degradation
8:99105387-Del	rs1282798097	ERICH5	intron_variant	Other	0.05	20.08	47453132.81	2.44E-02	Dis specific p=0.00284	mut count	Glutamate Rich 5	function not well known
6:66205610-Ins		EYS	intron_variant	Other	0.05	20.08	47453132.81	2.44E-02	no (dis specific p=0.0707	Race category, TMB, MSI, CMS,	Eyes Shut Homolog	Required to maintain the integrity of photoreceptor cells
5:118867238-Ins		HSD17B4	intron_variant	Other	0.05	20.08	47453132.81	2.44E-02	no (overall p=0.0527)	TMB, MSI,	Hydroxysteroid 17-Beta Dehydrogena	a bifunctional enzyme that is involved in the peroxisomal beta-oxidation pathway for fatty acids
14:56104660-Ins		KTN1	intron_variant	Other	0.05	20.08	47453132.81	2.44E-02	no	Race category, TMB, MSI, CIMP	Kinectin 1	localized to the endoplasmic reticulum membrane. binds kinesin and may be involved in intracellular organelle motility.
16:25189206-Ins-2		LCMT1	intron_variant	Other	0.05	20.08	47453132.81	2.44E-02	no		Leucine Carboxyl Methyltransferase 1	Methylates the carboxyl group of the C-terminal leucine residue of protein phosphatase 2A catalytic subunits to form alpha-leucine ester residues.
7:156476981-Del		LMBR1	intron_variant	Other	0.05	20.08	47453132.81	2.44E-02	no	Race category, TMB, MSI,	Limb Development Membrane Protei	disruption of this genic region can alter sonic hedgehog expression and affect limb patterning
2:170002171-Del		LRP2	intron_variant	Other	0.05	20.08	47453132.81	2.44E-02	no	MSI, race category, TMB, CMS1	LDL Receptor Related Protein 2	critical for the reuptake of numerous ligands, including lipoproteins, sterols, vitamin-binding proteins, and hormones
1:12032827-Ins		PLOD1	intron_variant	Other	0.05	20.08	47453132.81	2.44E-02	no	MSI, TMB, ICR, micro score, CIN	Procollagen-Lysine,2-Oxoglutarate 5-	localized to the cisternae of the endoplasmic reticulum.catalyzes the hydroxylation of lysyl residues in collagen-like peptides.
5:80762770-Ins		SSBP2	intron_variant	Other	0.05	20.08	47453132.81	2.44E-02	no	Race category, TMB, MSI	Single Stranded DNA Binding Protein 2	involved in the DNA damage response and maintenance of genome stability
6:144219898-Del		ZC2HC1B	intron_variant	Other	0.05	20.08	47453132.81	2.44E-02	no	Race category, TMB, MSI	Zinc Finger C2HC-Type Containing 1B	Predicted to enable metal ion binding activity.
7:103234986-Ins	rs35588028	RELN	intron_variant	Other	0.21	1.27	0.59	2.47E-02	no	TMB, race category, MSI, CMS	Reelin	a large secreted extracellular matrix protein thought to control cell-cell interactions critical for cell positioning and neuronal migration during brain development
5:148376982-SNV	rs1347130	SH3TC2	3_prime_UTR_variant	Other	0.29	1.10	0.51	2.53E-02	no	Race category, TMB, MSI, CIMP	SH3 Domain And Tetratricopeptide Re	proposed to be an adapter or docking molecule.
14:75366492-Ins		DLST	intron_variant	Other	0.06	20.01	47453132.81	2.79E-02	no	Race category, TMB,	Dihydrolipoamide S-Succinyltransfera	Dihydrolipoamide succinyltransferase (E2) component of the 2-oxoglutarate dehydrogenase complex.
3:5257384-Ins		EDEM1	intron_variant	Other	0.06	20.01	47453132.81	2.79E-02	Overall p=0.002308	Race category, TMB, MSI, ICR h	ER Degradation Enhancing Alpha-Mar	Extracts misfolded glycoproteins, but not glycoproteins undergoing productive folding, from the calnexin cycle. It is directly involved in endoplasmic reticulum-associated degradation (ERAD) and targets misfolded glycoproteins for degradation
7:106841786-Ins		HBP1	intron_variant	Other	0.06	20.01	47453132.81	2.79E-02	no	TMB, MSI, CIMP	HMG-Box Transcription Factor 1	Transcriptional repressor that binds to the promoter region of target genes. Plays a role in the regulation of the cell cycle and of the Wnt pathway.
6:129464881-Ins		LAMA2	intron_variant	Other	0.06	20.01	47453132.81	2.79E-02	Dis free p=0.0395	MSI, race category, TMB, CIMP	Laminin Subunit Alpha 2	thought to mediate the attachment, migration and organization of cells into tissues during embryonic development by interacting with other extracellular matrix components.
6:162206708-Ins-2		PRKN	intron_variant	Other	0.06	20.01	47453132.81	2.79E-02	Prog free p=0.0037	Race category, TMB, MSI, sex,	Parkin RBR E3 Ubiquitin Protein Ligas	a component of a multiprotein E3 ubiquitin ligase complex that mediates the targeting of substrate proteins for proteasomal degradation.
6:162206706-Ins		PRKN	intron_variant	Other	0.06	20.01	47453132.81	2.79E-02	see line 104			
4:2886471-Del		ADD1	intron_variant	Other	0.06	19.98	47453132.81	3.00E-02	no	Race category, TMB, MSI,	Adducin 1	Membrane-cytoskeleton-associated protein that promotes the assembly of the spectrin-actin network. Binds to calmodulin.
7:100061698-Ins		C7orf61	5_prime_UTR_variant	Other	0.06	19.98	47453132.81	3.00E-02	no	TMB, MSI, CIMP	SPACDR/Sperm Acrosome Developm	May play a role in acrosome formation and nucleus shaping during spermiogenesis.
7:116528060-Ins		CAPZA2	intron_variant	Other	0.06	19.98	47453132.81	3.00E-02	no	Race category, TMB, MSI	Capping Actin Protein Of Muscle Z-Lir	F-actin-capping proteins bind in a Ca(2+)-independent manner to the fast growing ends of actin filaments (barbed end) thereby blocking the exchange of subunits at these ends.
11:68542233-Ins		CPT1A	intron_variant	Other	0.06	19.98	47453132.81	3.00E-02	no	MSI, race category, TMB,	Carnitine Palmitoyltransferase 1A	CPT I is the key enzyme in the carnitine-dependent transport across the mitochondrial inner membrane and its deficiency results in a decreased rate of fatty acid beta-oxidation.
14:89210266-Ins-2		EML5	intron_variant	Other	0.06	19.98	47453132.81	3.00E-02	no	MSI, race category, TMB, CIMP,	EMAP Like 5	May modify the assembly dynamics of microtubules,
11:46914456-Del		LRP4	intron_variant	Other	0.06	19.98	47453132.81	3.00E-02	no	MSI, race category, TMB, CIMP,	LDL Receptor Related Protein 4	may be a regulator of Wnt signaling, Mediates SOST-dependent inhibition of bone formation.
1:156764385-Ins		PRCC	intron_variant	Other	0.06	19.98	47453132.81	3.00E-02	Prog free p=0.0283	Race category, TMB, MSI,	Proline Rich Mitotic Checkpoint Conti	may play a role in pre-mRNA splicing. May regulate cell cycle progression through interaction with MAD2L2.
7:127963422-Ins		RBM28	intron_variant	Other	0.06	19.98	47453132.81	3.00E-02	Dis specific p=0.0128	Race category, MSI, TMB, CIMP	RNA Binding Motif Protein 28	a specific nucleolar component of the spliceosomal small nuclear ribonucleoprotein (snRNP)complexes
15:34105538-Ins		RYR3	intron_variant	Other	0.06	19.98	47453132.81	3.00E-02	no	MSI, TMB, CIMP, race category,	Ryanodine Receptor 3	functions to release calcium from intracellular storage for use in many cellular processes.
6:10728726-Ins		TMEM14C	intron_variant	Other	0.06	19.98	47453132.81	3.00E-02	no	TMB, MSI	Transmembrane Protein 14C	Predicted to be involved in mitochondrial transport. Predicted to act upstream of or within erythrocyte differentiation and regulation of heme biosynthetic process.
8:21852904-Ins		XPO7	intron_variant	Other	0.06	19.98	47453132.81	3.00E-02	no	MSI, TMB,	Exportin 7	Mediates the nuclear export of proteins (cargos) with broad substrate specificity.
18:14104801-Ins		ZNF519	3_prime_UTR_variant	Other	0.06	19.98	47453132.81	3.00E-02	no	Race category, TMB, MSI	Zinc Finger Protein 519	May be involved in transcriptional regulation.
7:87149830-Ins		ABCB1	intron_variant	Other	0.07	19.94	47453132.81	3.24E-02	Overal p=0.0007919	Race category, TMB, MSI	ATP Binding Cassette Subfamily B Me	an ATP-binding cassette (ABC) transporter, involved in multidrug resistance
4:36152465-Ins		ARAP2	intron_variant	Other	0.07	19.94	47453132.81	3.24E-02	no	Race category, TMB, MSI,	ArfGAP With RhoGAP Domain, Ankyri	associates with focal adhesions and functions downstream of RhoA to regulate focal adhesion dynamics.
18:31226127-Ins		ASXL3	intron_variant	Other	0.07	19.94	47453132.81	3.24E-02	no (overall p=0.0750)	MSI, race category, TMB, CMS,	ASXL Transcriptional Regulator 3	negatively regulate lipogenesis by binding to and inhibiting the transcriptional activity of two nuclear hormone receptors, oxysterols receptor LXR-alpha (LXRalpha) and thyroid hormone receptor beta (TRbeta).
1:65142452-Ins		CACHD1	intron_variant	Other	0.07	19.94	47453132.81	3.24E-02	no (overall p=0.091)	TMB, race category, MSI,	Cache Domain Containing 1	May regulate voltage-dependent calcium channels.
11:72141200-Ins		CLPB	intron_variant	Other	0.07	19.94	47453132.81	3.24E-02	Overall p=0.0245	Race category, TMB, MSI,	ClpB Family Mitochondrial Disaggreg	cooperates with Hsp70 in the disaggregation of protein aggregates. Functions as a regulatory ATPase and participates in secretion/protein trafficking process.
9:128094072-Ins		GAPVD1	intron_variant	Other	0.07	19.94	47453132.81	3.24E-02	no	TMB, race category, MSI, CIMP,	GTPase Activating Protein And VPS9 D	Acts both as a GTPase-activating protein (GAP) and a guanine nucleotide exchange factor (GEF), and participates in various processes such as endocytosis, insulin receptor internalization or LC2A4/GLUT4 trafficking.
5:154299427-Ins-2		GEMIN5	intron_variant	Other	0.07	19.94	47453132.81	3.24E-02	no	MSI, race category, TMB, CIMP,	Gem Nuclear Organelle Associated Pr	a component of the survival of motor neurons (SMN) complex, plays a critical role in mRNA splicing through the assembly of spliceosomal small nuclear ribonucleoproteins (snRNPs)
5:143586158-Ins-2		KCTD16	5_prime_UTR_variant	Other	0.07	19.94	47453132.81	3.24E-02	no	TMB, CMS1 and 3, ICR high	Potassium Channel Tetramerization D	Auxiliary subunit of GABA-B receptors that determine the pharmacology and kinetics of the receptor response.
18:7034437-Ins		LAMA1	intron_variant	Other	0.07	19.94	47453132.81	3.24E-02	Overall p=0.0246	MSI, TMB, CMS, race category,	Laminin Subunit Alpha 1	one of the alpha 1 subunits of laminin, a major component of the basement membrane
6:160888506-Ins		LPAL2	non_coding_exon_variant	Other	0.07	19.94	47453132.81	3.24E-02	Overall p=0.0005864		Lipoprotein(A) Like 2 (Pseudogene)	a pseudogene similar to lipoprotein
3:37154289-Ins		LRRFIP2	intron_variant	Other	0.07	19.94	47453132.81	3.24E-02	no (prog free p=0.0545)	Race category, TMB, MSI,	LRR Binding FLII Interacting Protein 2	binds to the cytosolic tail of toll-like receptor 4 (TLR4), which results in activation of nuclear factor kappa B signaling, May function as activator of the canonical Wnt signaling pathway,
16:15138086-Ins		NTAN1,PDXDC1	intron_variant	Other	0.07	19.94	47453132.81	3.24E-02				
22:44286810-Ins		PNPLA5	intron_variant	Other	0.07	19.94	47453132.81	3.24E-02	Dis free p=0.0446	Race category, TMB, MSI,	Patatin Like Phospholipase Domain C	inhibit transacylation, Has abundant triacylglycerol lipase activity.
2:55883168-Del		PNPT1	intron_variant	Other	0.07	19.94	47453132.81	3.24E-02	no	Race category, TMB, MSI,	Polyribonucleotide Nucleotidyltransfe	predominantly localized in the mitochondrial intermembrane space and is involved in import of RNA to mitochondria.
10:38120454-Ins		ZNF248	3_prime_UTR_variant	Other	0.07	19.94	47453132.81	3.24E-02	Overall p=0.0186	TMB, race category, MSI,	Zinc Finger Protein 248	May be involved in transcriptional regulation.
1:71544080-Del	rs10574763	ZRANB2	intron_variant	Other	0.07	19.94	47453132.81	3.24E-02	no	MSI, race category, TMB, CIMP,	Zinc Finger RANBP2-Type Containing	Splice factor required for alternative splicing of TRA2B/SFRS10 transcripts.
19:13915508-Ins		ZSWIM4	intron_variant	Other	0.07	19.94	47453132.81	3.24E-02	no (dis free p=0.0527)	MSI, race category, TMB, CIMP,	Zinc Finger SWIM-Type Containing 4	Predicted to be part of Cul2-RING ubiquitin ligase complex
13:19744432-Ins					0.07	19.94	47453132.81	3.24E-02				
1:94884290-Del		ABCD3	intron_variant	Other	0.07	19.65	33554432.00	3.53E-02	no	Race category, MSI, TMB,	ATP Binding Cassette Subfamily D Me	involved in peroxisomal import of fatty acids and/or fatty acyl-CoAs in the organelle
3:182537947-Ins		ATP11B	intron_variant	Other	0.07	19.65	33554432.00	3.53E-02	no	Race category, TMB, MSI, CIMP	ATPase Phospholipid Transporting 11	Catalytic component of a P4-ATPase flippase complex which catalyzes the hydrolysis of ATP coupled to the transport of aminophospholipids, phosphatidylserines (PS) and phosphatidylethanolamines (PE), from the outer to the inner leaflet of intracellular membranes
6:53363506-Ins		GCLC	3_prime_UTR_variant	Other	0.07	19.65	33554432.00	3.53E-02	no (dis specific p=0.0853	Race category, TMB,	Glutamate-Cysteine Ligase Catalytic S	the first rate-limiting enzyme of glutathione synthesis
22:26086072-Ins		GRK3	intron_variant	Other	0.07	19.65	33554432.00	3.53E-02	no	Race category, TMB, MSI,	G Protein-Coupled Receptor Kinase 3	Specifically phosphorylates the agonist-occupied form of the beta-adrenergic and closely related receptors.
11:27400091-Ins		LGR4	intron_variant	Other	0.07	19.65	33554432.00	3.53E-02	no	Race category, TMB, MSI, CIMF	Leucine Rich Repeat Containing G Pro	a G-protein coupled receptor that binds R-spondins and activates the Wnt signaling pathway
16:15839438-Del	rs1421049064	MYH11	intron_variant	Other	0.07	19.65	33554432.00	3.53E-02	Overall p=0.0482	MSI, race category, TMB, CIMP,	Myosin Heavy Chain 11	a major contractile protein, converting chemical energy into mechanical energy through the hydrolysis of ATP.
10:82185916-Ins		PRXL2A	intron_variant	Other	0.07	19.65	33554432.00	3.53E-02	no	MSI	Peroxiredoxin Like 2A	Involved in redox regulation of the cell, Acts as a negative regulator of macrophage-mediated inflammation by inhibiting macrophage production of inflammatory cytokines, probably through suppression of the MAPK signaling pathway
6:72993631-Ins		RIMS1	intron_variant	Other	0.07	19.65	33554432.00	3.53E-02	Overall p=0.0000948	MSI, race category, TMB, CMS,	Regulating Synaptic Membrane Exocy	a RAS gene superfamily member that regulates synaptic vesicle exocytosis.
21:11021164-Ins	rs145115496	BAGE2	3_prime_UTR_variant	Other	0.17	1.45	0.72	3.53E-02	Dis free p=0.0491	Race category, sex, MSI	BAGE Family Member 2 (Pseudogene)	Unknown. Candidate gene encoding tumor antigens.
9:134391433-Ins	rs796366369	POMT1	intron_variant	Other	0.22	1.13	0.55	3.70E-02	no	MSI, race category, TMB, CIMP	Protein O-Mannosyltransferase 1	Transfers mannosyl residues to the hydroxyl group of serine or threonine residues. found in the membrane of the endoplasmic reticulum
10:106970849-Del	rs3214966	SORCS3	intron_variant	Other	0.18	1.42	0.72	3.91E-02	no	Race category, TMB, MSI, CIMF	Sortilin Related VPS10 Domain Conta	a member of the vacuolar protein sorting 10 receptor family
18:6890408-Ins		ARHGAP28	intron_variant	Other	0.05	19.76	47453132.81	3.99E-02	no	Race category, TMB, MSI,	Rho GTPase Activating Protein 28	GTPase activator for the Rho-type GTPases by converting them to an inactive GDP-bound state.
10:60047272-Ins		CISD1	intron_variant	Other	0.05	19.76	47453132.81	3.99E-02	no		CDGSH Iron Sulfur Domain 1	localized to the outer membrane of mitochondria and is thought to play a role in regulation of oxidation.
11:64825268-Ins		NAALADL1	intron_variant	Other	0.05	19.76	47453132.81	3.99E-02	no (prog free p=0.0931)	MSI, race category, TMB, CIMP,	N-Acetylated Alpha-Linked Acidic Dip	Aminopeptidase with broad substrate specificity.
17:47780450-Del		SLC35B1	intron_variant	Other	0.05	19.76	47453132.81	3.99E-02	Overall p=0.0476	Race category, TMB	Solute Carrier Family 35 Member B1	Imports ATP from the cytosol to the ER lumen and exports ADP in the opposite direction, Regulates ER energy metabolism and protein biogenesis.
16:66545807-Ins		TK2	3_prime_UTR_variant	Other	0.05	19.76	47453132.81	3.99E-02	no (overall p=0.0533)	Race category, TMB	Thymidine Kinase 2	a deoxyribonucleoside kinase that specifically phosphorylates thymidine, deoxycytidine, and deoxyuridine. The encoded enzyme localizes to the mitochondria and is required for mitochondrial DNA synthesis.
7:150666559-Ins		KCNH2	intron_variant	Other	0.11	1.32	0.69	4.63E-02	no	MSI, race category, TMB, CIMP,	Potassium Voltage-Gated Channel Su	a component of a voltage-activated potassium channel found in cardiac muscle, nerve cells, and microglia.
7:150666556-Ins		KCNH2	intron_variant	Other	0.11	1.32	0.69	4.63E-02	see line 153			
4:25391986-Ins		ANAPC4	intron_variant	Other	0.09	19.76	47453132.81	4.74E-02	no	Race category, TMB,	Anaphase Promoting Complex Subuni	Component of the anaphase promoting complex/cyclosome (APC/C), a cell cycle-regulated E3 ubiquitin ligase that controls progression through mitosis and the G1 phase of the cell cycle.
16:24941975-Ins		ARHGAP17	intron_variant	Other	0.09	19.76	47453132.81	4.74E-02	Dis free p=0.006566	Race category, TMB, MSI, CMS,	Rho GTPase Activating Protein 17	Rho GTPase-activating protein involved in the maintenance of tight junction by regulating the activity of CDC42, thereby playing a central role in apical polarity of epithelial cells.
2:127841378-Del		BIN1	5_prime_UTR_variant	Other	0.09	19.76	47453132.81	4.74E-02	Prog free p=0.004467	Race category, TMB, MSI	Bridging Integrator 1	a key player in the control of plasma membrane curvature, membrane shaping and membrane remodeling.
3:53778970-Ins		CACNA1D	intron_variant	Other	0.09	19.76	47453132.81	4.74E-02	no	MSI, race category, TMB, CIMP,	Calcium Voltage-Gated Channel Subu	Voltage-sensitive calcium channels (VSCC) mediate the entry of calcium ions into excitable cells and are also involved in a variety of calcium-dependent processes, including muscle contraction, hormone or neurotransmitter release, gene expression, cell motility, cell division ar
11:104896844-SNV	rs1977989	CASP1	3_prime_UTR_variant	Other	0.09	19.76	47453132.81	4.74E-02	no	Race category, TMB, MSI	Caspase 1	Thiol protease involved in a variety of inflammatory processes by proteolytically cleaving other proteins, such as the precursors of the inflammatory cytokines interleukin-1 beta (IL1B) and interleukin 18 (IL18) as well as the pyroptosis inducer Gasdermin-D (GSDMD), into active
4:77246888-Ins		CCDC158	intron_variant	Other	0.09	19.76	47453132.81	4.74E-02	no	Race category, TMB, MSI, CIMF	Coiled-Coil Domain Containing 158	An important paralog of this gene is MYH8 (Myosin Heavy Chain 8)
16:17232129-Ins		LOC102723692,XYL	intron_variant	Other	0.09	19.76	47453132.81	4.74E-02				
12:113407275-Ins		OAS3	intron_variant	Other	0.09	19.76	47453132.81	4.74E-02	no	Race category, TMB, CMS1, MS	2'-5'-Oligoadenylate Synthetase 3	plays a significant role in the inhibition of cellular protein synthesis and viral infection resistance.
5:133535919-Ins-3		PPP2CA	intron_variant	Other	0.09	19.76	47453132.81	4.74E-02	no	TMB, MSI	Protein Phosphatase 2 Catalytic Subu	the major phosphatase for microtubule-associated proteins (MAPs)
12:1021159-SNV	rs10849584	RAD52	3_prime_UTR_variant	Other	0.09	19.76	47453132.81	4.74E-02	Overall p=0.0457	Race category, TMB	RAD52 Homolog, DNA Repair Protein	Involved in double-stranded break repair.
19:51216938-Ins		SHANK1	intron_variant	Other	0.09	19.76	47453132.81	4.74E-02	no	MSI, race category, TMB, CMS	SH3 And Multiple Ankyrin Repeat Don	Plays a role in the structural and functional organization of the dendritic spine and synaptic junction.
19:44634929-Ins		ZNF225	intron_variant	Other	0.09	19.76	47453132.81	4.74E-02	no	Race category, TMB	Zinc Finger Protein 225	May be involved in transcriptional regulation.
19:10370542-SNV	rs11115	MRPL4	3_prime_UTR_variant	Other	0.24	1.07	0.55	4.78E-02	no	MSI, TMB	Mitochondrial Ribosomal Protein L4	encodes a 39S subunit protein of mitochondrial ribosome

Supplementary Table 3a American Indian

Predictor	Identifier	Gene Names	Sequence Ontology	Effect	MAF	Beta	log2FoldChang	P-Value	survival influence (p<0.05)?	other notables p<0.05	full gene name	protein and function	CRC publication#	Cancer pub #	All publication#
5:112047955-Ins	rs72545070	APC	intron_variant	Other	0.32	1.56	-0.668943896	2.86E-03	overall 0.0231	MSI, Race category, TMB, CIMP, CMS	Adenomatosis Polyposis Coli	a tumor suppressor protein that acts as an antagonist of the Wnt signaling pathway	1580	3942	5072
11:51516036-SNV	rs11246608	OR4C46	missense_variant	Missense	0.26	3.91	-0.52560157	3.00E-03	dis specific 0.0457	MSI, TMB, Race catgory,	Olfactory Receptor Family 4 Subfamily C Member 46	Odorant receptor. G-protein coupled receptor	0	0	0
1:153926916-Del		CRTC2	intron_variant	Other	0.13	19.94	0.313932567	4.10E-03	Dis free 0.0480	MSI, TMB	CREB Regulated Transcription Coactivator 2	transcriptional coactivator for CREB1	5	170	482
17:28811150-Ins		GOSR1	intron_variant	Other	0.13	19.65	-0.25347783	4.78E-03	Dis free 0.004, oveall 0.0147	Race category, TMB	Golgi SNAP Receptor Complex Member 1	an essential component of the Golgi SNAP receptor (SNARE) complex	0	9	33
18:42646635-Del-2	rs34125334	SETBP1	3_prime_UTR_variant	Other	0.21	1.87	-0.092481398	5.49E-03	overall 0.0099	TMB, MSI, CMS1, ICR high, race category, CIMP	SET Binding Protein 1	bind the SET nuclear oncogene which is involved in DNA replication	1	208	319
2:51256013-SNV	rs62143026	NRXN1	_prime_UTR_premature_start_codon_gain_varia	Missense	0.07	19.51	-0.355708296	9.26E-03	Dis free 0.0256	MSI, TMB, CIMP, Race cetegory	Neurexin 1	Cell surface protein involved in cell-cell-interactions, exocytosis of secretory granules and regulation o	1	40	479
3:68934434-SNV	rs4855535	TAFA4	5_prime_UTR_variant	Other	0.09	19.53	-0.481900206	9.78E-03	Dis free 0.00155	Race category, MSI	TAFA Chemokine Like Family Member 4	postulated to function as brain-specific chemokines or neurokines, that act as regulators of immune a	0	22	38
8:134308938-Del	rs36215434	NDRG1	intron_variant	Other	0.25	1.75	0.270232705	1.01E-02	Dis specific 0.00851	mut count	N-Myc Downstream Regulated 1	involved in stress responses, hormone responses, cell growth, and differentiation	27	530	757
4:54139976-Del	rs150594118	SCFD2	intron_variant	Other	0.14	1.79	-0.338236487	1.54E-02	Overall p=0.004482	Race category, TMB, MSI	Sec1 Family Domain Containing 2	Predicted to be involved in intracellular protein transport and vesicle docking involved in exocytosis.	0	8	11
15:55881107-SNV	rs1992237	PYGO1	5_prime_UTR_variant	Other	0.27	1.21	-0.300536862	1.66E-02	Prog free p=0.0334	Race category, MSI, TMB, CIMP, sex	Pygopus Family PHD Finger 1	Involved in signal transduction through the Wnt pathway.	3	16	33
10:97366107-SNV	rs8758	ALDH18A1	3_prime_UTR_variant	Other	0.19	1.35	-0.538300089	1.66E-02	Overall p=0.002169	TMB, race category, MSI, CMS1	Aldehyde Dehydrogenase 18 Family Member A1	a member of the aldehyde dehydrogenase family and encodes a bifunctional ATP- and NADPH-depe	0	31	93
22:23603475-Ins		BCR	intron_variant	Other	0.05	19.91	0.223184195	2.06E-02	Overall p=0.0473	MSI, race category, TMB,	BCR Activator Of RhoGEF And GTPase	Although the BCR-ABL fusion protein has been extensively studied, the function of the normal BCR ge	0	37	98
2:227915536-Ins		COL4A4	intron_variant	Other	0.05	19.91	-0.335637299	2.06E-02	Prog free p=0.0211	Race category, TMB,	Collagen Type IV Alpha 4 Chain	one of the six subunits of type IV collagen	0	29	435
2:44127040-Del		LRPPRC	intron_variant	Other	0.05	19.91	-0.616442781	2.06E-02	Prog free p=0.0149	MSI, TMB, race category, CIMP, CMS,	Leucine Rich Pentatricopeptide Repeat Containing	May play a role in RNA metabolism in both nuclei and mitochondria	2	92	240
1:72400727-Ins		NEGR1	intron_variant	Other	0.05	19.91	-0.309822414	2.06E-02	Overall p=0.00346		Neuronal Growth Regulator 1	May function as a trans-neural growth-promoting factor in regenerative axon sprouting in the mamm	2	37	194
2:214204856-Ins-2		SPAG16	intron_variant	Other	0.05	19.97	-0.676057243	2.17E-02	Dis free p=0.0344	TMB, MSI	Sperm Associated Antigen 16	Necessary for sperm flagellar function. Plays a role in motile ciliogenesis.	1	8	47
8:99105387-Del	rs1282798097	ERICH5	intron_variant	Other	0.05	20.08	-0.138482655	2.44E-02	Dis specific p=0.00284	mut count	Glutamate Rich 5	function not well known	0	0	1
3:5257384-Ins		EDEM1	intron_variant	Other	0.06	20.01	-0.139015765	2.79E-02	Overall p=0.002308	Race category, TMB, MSI, ICR high	ER Degradation Enhancing Alpha-Mannosidase Like Protein 1	Extracts misfolded glycoproteins, but not glycoproteins undergoing productive folding, from the caln	1	21	153
6:129464881-Ins		LAMA2	intron_variant	Other	0.06	20.01	-0.587306702	2.79E-02	Dis free p=0.0395	MSI, race category, TMB, CIMP	Laminin Subunit Alpha 2	thought to mediate the attachment, migration and organization of cells into tissues during embryonic	3	63	430
6:162206708-Ins-2		PRKN	intron_variant	Other	0.06	20.01	-0.106401704	2.79E-02	Prog free p=0.0037	Race category, TMB, MSI, sex,	Parkin RBR E3 Ubiquitin Protein Ligase	a component of a multiprotein E3 ubiquitin ligase complex that mediates the targeting of substrate p	1	69	476
1:156764385-Ins		PRCC	intron_variant	Other	0.06	19.98	-0.173340203	3.00E-02	Prog free p=0.0283	Race category, TMB, MSI,	Proline Rich Mitotic Checkpoint Control Factor	may play a role in pre-mRNA splicing. May regulate cell cycle progression through interaction with MA	4	705	851
7:127963422-Ins		RBM28	intron_variant	Other	0.06	19.98	-0.00917593	3.00E-02	Dis specific p=0.0128	Race category, MSI, TMB, CIMP	RNA Binding Motif Protein 28	a specific nucleolar component of the spliceosomal small nuclear ribonucleoprotein (snRNP)complexe	0	6	17
7:87149830-Ins		ABCB1	intron_variant	Other	0.07	19.94	-0.300762282	3.24E-02	Overal p=0.0007919	Race category, TMB, MSI,	ATP Binding Cassette Subfamily B Member 1	an ATP-binding cassette (ABC) transporter, involved in multidrug resistance	200	3979	7448
11:72141200-Ins		CLPB	intron_variant	Other	0.07	19.94	-0.11102142	3.24E-02	Overall p=0.0245	Race category, TMB, MSI,	ClpB Family Mitochondrial Disaggregase	cooperates with Hsp70 in the disaggregation of protein aggregates. Functions as a regulatory ATPase	0	35	692
18:7034437-Ins		LAMA1	intron_variant	Other	0.07	19.94	-0.403624104	3.24E-02	Overall p=0.0246	MSI, TMB, CMS, race category, CIMP, ICR high, micro score, sex,	Laminin Subunit Alpha 1	one of the alpha 1 subunits of laminin, a major component of the basement membrane	3	41	170
6:160888506-Ins		LPAL2	non_coding_exon_variant	Other	0.07	19.94	-0.574029135	3.24E-02	Overall p=0.0005864		Lipoprotein(A) Like 2 (Pseudogene)	a pseudogene similar to lipoprotein	0	4	17
22:44286810-Ins		PNPLA5	intron_variant	Other	0.07	19.94	0.360257292	3.24E-02	Dis free p=0.0446	Race category, TMB, MSI,	Patatin Like Phospholipase Domain Containing 5	inhibit transacylation, Has abundant triacylglycerol lipase activity.	0	4	14
10:38120454-Ins		ZNF248	3_prime_UTR_variant	Other	0.07	19.94	-0.499141587	3.24E-02	Overall p=0.0186	TMB, race category, MSI,	Zinc Finger Protein 248	May be involved in transcriptional regulation.	0	0	4
16:15839438-Del	rs1421049064	MYH11	intron_variant	Other	0.07	19.65	-0.178896795	3.53E-02	Overall p=0.0482	MSI, race category, TMB, CIMP, CMS	Myosin Heavy Chain 11	a major contractile protein, converting chemical energy into mechanical energy through the hydrolysis	6	590	973
6:72993631-Ins		RIMS1	intron_variant	Other	0.07	19.65	-0.237452932	3.53E-02	Overall p=0.0000948	MSI, race category, TMB, CMS,	Regulating Synaptic Membrane Exocytosis 1	a RAS gene superfamily member that regulates synaptic vesicle exocytosis.	0	14	148
21:11021164-Ins	rs145115496	BAGE2	3_prime_UTR_variant	Other	0.17	1.45	-0.529436866	3.53E-02	Dis free p=0.0491	Race category, sex, MSI	BAGE Family Member 2 (Pseudogene)	Unknown. Candidate gene encoding tumor antigens.	1	4	8
17:47780450-Del		SLC35B1	intron_variant	Other	0.05	19.76	0.044222516	3.99E-02	Overall p=0.0476	Race category, TMB	Solute Carrier Family 35 Member B1	Imports ATP from the cytosol to the ER lumen and exports ADP in the opposite direction, Regulates E	0	1	12
16:24941975-Ins		ARHGAP17	intron_variant	Other	0.09	19.76	0.025098443	4.74E-02	Dis free p=0.006566	Race category, TMB, MSI, CMS, CIMP	Rho GTPase Activating Protein 17	Rho GTPase-activating protein involved in the maintenance of tight junction by regulating the activity	2	11	31
2:127841378-Del		BIN1	5_prime_UTR_variant	Other	0.09	19.76	0.762904004	4.74E-02	Prog free p=0.004467	Race category, TMB, MSI	Bridging Integrator 1	a key player in the control of plasma membrane curvature, membrane shaping and membrane remod	12	511	1661
12:1021159-SNV	rs10849584	RAD52	3_prime_UTR_variant	Other	0.09	19.76	-0.124188195	4.74E-02	Overall p=0.0457	Race category, TMB	RAD52 Homolog, DNA Repair Protein	Involved in double-stranded break repair.			

Supplementary Table 3b American Indian SCGs

Whites

Predictor	Identifier	Gene Names	Sequence Ontology	Effect	Minor Allele	Major Allele	MAF	Beta	SE	P-Value	FDR	TF	GnomAD	CADD	ClinVar	miRNA
1:17420276-Del		PADI2	intron_variant	Other	-	T	0.30	18.80	27397079.00	4.83E-02	4.90E-02					
2:121555236-Ins	rs58254611	GLI2	intron_variant	Other	CCA	-	0.30	18.80	27397079.00	4.83E-02	4.90E-02					
3:9825555-Ins		TADA3	3_prime_UTR_variant	Other	T	-	0.30	18.80	27397079.00	4.83E-02	4.90E-02					
4:4270106-Ins		LYAR	intron_variant	Other	TG	-	0.30	18.80	27397079.00	4.83E-02	4.90E-02					
4:71628083-Ins		RUFY3	intron_variant	Other	GT	-	0.30	18.80	27397079.00	4.83E-02	4.90E-02					
5:37026201-Ins		NIPBL	intron_variant	Other	CT	-	0.30	18.80	27397079.00	4.83E-02	4.90E-02					
6:149982749-Ins		LATS1	3_prime_UTR_variant	Other	T	-	0.40	18.66	23726566.41	4.83E-02	4.90E-02					
6:152711928-Ins		SYNE1	intron_variant	Other	TG	-	0.30	18.80	27397079.00	4.83E-02	4.90E-02					
7:87146048-Del	rs546527484	ABCB1	intron_variant	Other	-	TTTTT	0.40	18.66	23726566.41	4.83E-02	4.90E-02	NA	A	4.24		
11:61071219-Ins		DDB1	intron_variant	Other	T	-	0.30	18.80	27397079.00	4.83E-02	4.90E-02					
11:67160413-Ins		RAD9A	intron_variant	Other	A	-	0.30	18.80	27397079.00	4.83E-02	4.90E-02					
4:71634492-Del		RUFY3	intron_variant	Other	-	G	0.14	19.01	33554432.00	4.99E-02	4.99E-02					
5:70939332-Del		MCCC2	intron_variant	Other	-	G	0.14	19.01	33554432.00	4.99E-02	4.99E-02					
10:73565338-Ins		CDH23	intron_variant	Other	GT	-	0.14	19.01	33554432.00	4.99E-02	4.99E-02					
3:33076098-Ins	rs952726547	GLB1	intron_variant	Other	T	-	0.25	18.77	27397079.00	4.73E-02	5.08E-02	NA	A	6.44		
4:10559856-Ins		CLNK	intron_variant	Other	T	-	0.25	18.77	27397079.00	4.73E-02	5.08E-02					
5:93987396-Ins		SLF1	intron_variant	Other	CT	-	0.25	18.77	27397079.00	4.73E-02	5.08E-02					
7:138189190-Del	rs763671174	TRIM24	intron_variant	Other	-	CTCTTTT	0.25	18.77	27397079.00	4.73E-02	5.08E-02					
8:33229978-Ins		FUT10	3_prime_UTR_variant	Other	CT	-	0.25	18.77	27397079.00	4.73E-02	5.08E-02					
8:97154319-Ins		GDF6	downstream_gene_variant	Unknown	GT	-	0.25	18.77	27397079.00	4.73E-02	5.08E-02					
10:226163-Del		ZMYND11	intron_variant	Other	-	T	0.25	18.77	27397079.00	4.73E-02	5.08E-02					
11:10514803-Ins		AMPD3	intron_variant	Other	T	-	0.25	18.77	27397079.00	4.73E-02	5.08E-02					
22:18910285-Del	rs5844366	PRODH	intron_variant	Other	C	-	0.43	19.63	19372660.35	2.54E-04	5.09E-02					
12:66638665-Ins		IRAK3	intron_variant	Other	GT	-	0.21	18.75		4.65E-02	5.28E-02					
11:26529951-Del		ANO3	intron_variant	Other	-	C	0.25	18.77	27397079.00	4.73E-02	5.31E-02					
15:76222522-Del		FBXO22	3_prime_UTR_variant	Other	-	T	0.25	18.77	27397079.00	4.73E-02	5.31E-02					
1:118634155-Ins		SPAG17	intron_variant	Other	GT	-	0.21	18.75	27397079.00	4.65E-02	5.31E-02					
14:60475025-Ins		LRRC9	intron_variant	Other	A	-	0.21	18.75	27397079.00	4.65E-02	5.31E-02					
19:19004466-Del		CERS1,GDF1	intron_variant	Other	-	G	0.21	18.75	27397079.00	4.65E-02	5.31E-02					
1:225541605-Ins		DNAH14	intron_variant	Other	A	-	0.21	18.75		4.65E-02	5.41E-02					
10:99139583-Del		RRP12	intron_variant	Other	-	G	0.21	18.75	27397079.00	4.65E-02	5.41E-02					
16:67899265-Del		NUTF2	intron_variant	Other	-	G	0.21	18.75	27397079.00	4.65E-02	5.41E-02					
12:76442907-SNV	rs11180815	NAP1L1	3_prime_UTR_variant	Other	C	T	0.20	18.53	23726566.41	4.64E-02	5.49E-02	A	A	5		A
20:31292299-Del	rs59379723	COMMD7	splice_region_variant	Other	A	-	0.20	18.53	23726566.41	4.64E-02	5.49E-02					
2:85510481-Ins		TCF7L1	intron_variant	Other	GT	-	0.19	18.74	27397079.00	4.59E-02	5.50E-02					
6:38810040-Ins		DNAH8	intron_variant	Other	CT	-	0.19	18.49	19372660.35	4.59E-02	5.50E-02					
8:38172087-Del	rs1212277088	NSD3	intron_variant	Other	-	CCTA	0.19	18.74	27397079.00	4.59E-02	5.50E-02	NA	A	5.13		
9:103309971-Ins		MSANTD3-TMEFF1,TM	intron_variant	Other	GT	-	0.19	18.49	19372660.35	4.59E-02	5.50E-02					
14:95081463-Del		SERPINA3	intron_variant	Other	-	GG	0.14	18.77	27397079.00	3.74E-02	5.58E-02					
15:73852260-Ins		REC114	3_prime_UTR_variant	Other	A	-	0.14	18.77	27397079.00	3.74E-02	5.58E-02					
1:17387860-Ins					T	-	0.16	18.79	27397079.00	3.69E-02	5.63E-02					
2:26477129-Ins		HADHB	inframe_insertion	Missense	-	ACA	0.16	18.79	27397079.00	3.69E-02	5.63E-02					
5:41805572-Ins		OXCT1	intron_variant	Other	CT	-	0.16	18.79	27397079.00	3.69E-02	5.63E-02					
5:138171074-Ins-2		CTNNA1	intron_variant	Other	T	-	0.16	18.79	27397079.00	3.69E-02	5.63E-02					
12:27180189-Ins		MED21	intron_variant	Other	T	-	0.16	18.79	27397079.00	3.69E-02	5.63E-02					
12:120221620-Del		CIT	intron_variant	Other	-	G	0.16	18.79	27397079.00	3.69E-02	5.63E-02					
15:48427021-Ins		MYEF2,SLC24A5	3_prime_UTR_variant	Other	T	-	0.16	18.79	27397079.00	3.69E-02	5.63E-02					
19:36585274-Ins	rs1972826993	WDR62	intron_variant	Other	A	-	0.16	18.79	27397079.00	3.69E-02	5.63E-02	NA	NA			
3:132295651-Ins		ACAD11,NPHP3-ACAI	intron_variant	Other	GT	-	0.19	18.77	27397079.00	4.59E-02	5.63E-02					
21:45500092-Ins		TRAPPC10	intron_variant	Other	AC	-	0.14	18.77	27397079.00	3.74E-02	5.66E-02					
10:85984006-Ins		LRIT2	intron_variant	Other	T	-	0.19	18.74	27397079.00	4.59E-02	5.67E-02					
12:32963562-Ins		PKP2	intron_variant	Other	A	-	0.19	18.49	19372660.35	4.59E-02	5.67E-02					
13:32892973-Ins		BRCA2	intron_variant	Other	T	-	0.19	18.49	19372660.35	4.59E-02	5.67E-02					
1:92944337-Ins		GFI1	intron_variant	Other	AGAGAC	-	0.33	1.15	0.63	3.88E-02	5.70E-02					
15:76577888-Ins		ETFA	intron_variant	Other	T	-	0.21	18.59	23726566.41	3.86E-02	5.71E-02					
1:63299891-Del		ATG4C	intron_variant	Other	-	T	0.22	19.12	33554432.00	4.58E-02	5.76E-02					
3:27431393-Ins		SLC4A7	intron_variant	Other	GT	-	0.22	19.12	33554432.00	4.58E-02	5.76E-02					
5:78324211-Ins		DMGDH	intron_variant	Other	T	-	0.22	19.12	33554432.00	4.58E-02	5.76E-02					
7:95221694-Ins		PDK4	intron_variant	Other	GT	-	0.22	19.12	33554432.00	4.58E-02	5.76E-02					
13:79940644-Ins-3		RBM26	intron_variant	Other	T	-	0.22	19.12	33554432.00	4.58E-02	5.76E-02					
14:51096508-Ins		ATL1	intron_variant	Other	T	-	0.22	19.12	33554432.00	4.58E-02	5.76E-02					
22:50927790-Ins		MIOX	intron_variant	Other	GT	-	0.22	19.12	33554432.00	4.58E-02	5.76E-02					
5:140048707-SNV	rs35983033	WDR55	missense_variant	Missense	G	A	0.17	18.56	23726566.41	3.97E-02	5.80E-02					
20:61524385-Del	rs11362069	DIDO1	intron_variant	Other	T	-	0.29	18.66	23726566.41	3.62E-02	5.89E-02	NA	A	0.65		
7:99013991-Del		BUD31	intron_variant	Other	-	C	0.33	18.66	23726566.41	4.08E-02	5.91E-02					
1:161476597-Ins	rs1412635201	FCGR2A	intron_variant	Other	A	-	0.15	18.72	27397079.00	4.50E-02	5.93E-02	NA	A	10		
12:999542-Ins		WNK1	intron_variant	Other	TGT	-	0.15	18.72	27397079.00	4.50E-02	5.93E-02					
17:29576977-Ins		NF1	intron_variant	Other	T	-	0.15	18.72	27397079.00	4.50E-02	5.93E-02					
6:6318922-Ins	rs398000295	F13A1	splice_region_variant	Other	-	A	0.23	-1.13	0.62	4.12E-02	5.93E-02	NA	A	2.1	Benign	
2:216295342-Ins		FN1	intron_variant	Other	T	-	0.18	18.80	27397079.00	3.62E-02	5.94E-02					
12:101012177-Ins		GAS2L3	intron_variant	Other	GT	-	0.18	18.80	27397079.00	3.62E-02	5.94E-02					
14:21990772-Ins	rs1018987788	SALL2	3_prime_UTR_variant	Other	T	-	0.18	18.80	27397079.00	3.62E-02	5.94E-02	NA	A	1.75		A
15:40949712-Del		KNL1	intron_variant	Other	-	T	0.18	18.80	27397079.00	3.62E-02	5.94E-02					
15:41028609-Ins		RMDN3	3_prime_UTR_variant	Other	GT	-	0.18	18.80	27397079.00	3.62E-02	5.94E-02					
17:40647286-Ins		ATP6V0A1	intron_variant	Other	AC	-	0.18	18.80	27397079.00	3.62E-02	5.94E-02					
13:32977677-Ins		N4BP2L1	intron_variant	Other	T	-	0.17	19.08	33554432.00	4.33E-02	5.97E-02					
3:71050020-Ins-3		FOXP1	intron_variant	Other	CTTT	-	0.17	19.08	33554432.00	4.33E-02	6.01E-02					
12:88477495-Ins		CEP290	intron_variant	Other	T	-	0.17	19.08	33554432.00	4.33E-02	6.01E-02					
16:28840590-Ins		ATXN2L	intron_variant	Other	T	-	0.17	19.08	33554432.00	4.33E-02	6.01E-02					

(whites_continued1)

11:61165732-Ins	rs11382548	TMEM216	frameshift_variant	LoF	-	A	0.32	1.03	0.59	4.25E-02	6.03E-02					
15:89753220-SNV	rs2710	RLBP1	3_prime_UTR_variant	Other	T	C	0.32	1.03	0.59	4.25E-02	6.03E-02	A	A	0.265	Benign	A
6:73820971-Ins		KCNQ5	intron_variant	Other	T	-	0.15	18.72	27397079.00	4.50E-02	6.04E-02					
16:47697715-Ins		PHKB	intron_variant	Other	A	-	0.15	18.72	27397079.00	4.50E-02	6.04E-02					
6:16306507-Ins		ATXN1	3_prime_UTR_variant	Other	T	-	0.14	18.75	27397079.00	4.47E-02	6.08E-02					
7:16130380-Ins		CRPPA	3_prime_UTR_variant	Other	T	-	0.14	18.75	27397079.00	4.47E-02	6.08E-02					
2:211521227-Del		CPS1	intron_variant	Other	-	CTT	0.18	18.80	27397079.00	3.62E-02	6.24E-02					
3:130687973-Ins		ATP2C1	intron_variant	Other	T	-	0.18	18.80	27397079.00	3.62E-02	6.24E-02					
5:147513280-Del		SPINK5	intron_variant	Other	-	C	0.18	18.80	27397079.00	3.62E-02	6.24E-02					
4:68357747-Ins		CENPC	intron_variant	Other	T	-	0.20	18.58	19372660.35	3.54E-02	6.26E-02					
4:69188417-Ins		YTHDC1	intron_variant	Other	T	-	0.20	18.58	19372660.35	3.54E-02	6.26E-02					
12:9220663-Ins		A2M	intron_variant	Other	GT	-	0.20	18.58	19372660.35	3.54E-02	6.26E-02					
16:81705792-Del		CMIP	intron_variant	Other	-	GGC	0.20	18.65	19372660.35	3.54E-02	6.26E-02					
18:12093931-Ins		ANKRD62	5_prime_UTR_variant	Other	T	-	0.20	18.83	27397079.00	3.54E-02	6.26E-02					
22:31980957-Ins		SFI1	intron_variant	Other	T	-	0.20	18.58	19372660.35	3.54E-02	6.26E-02					
6:152737500-Ins		SYNE1	intron_variant	Other	T	-	0.20	18.58	19372660.35	3.54E-02	6.61E-02					
5:121776565-Ins		MGC32805,SNCAIF	intron_variant	Other	A	-	0.15	19.15	33554432.00	3.53E-02	6.65E-02					
16:71571834-Del		CHST4	3_prime_UTR_variant	Other	-	T	0.15	19.15	33554432.00	3.53E-02	6.65E-02					
16:71571835-Ins		CHST4	3_prime_UTR_variant	Other	AC	-	0.15	19.15	33554432.00	3.53E-02	6.65E-02					
15:45445690-Del	rs758869498	DUOX1	intron_variant	Other	-	GAATGTGTGTGTG	0.44	1.18	0.62	3.26E-02	6.78E-02	A	A	0.004		NA
2:75881800-Ins-2		MRPL19	intron_variant	Other	T	-	0.27	18.92	27397079.00	3.25E-02	6.84E-02					
2:158406592-Ins-2		ACVR1C	intron_variant	Other	T	-	0.27	18.92	27397079.00	3.25E-02	6.84E-02					
2:234054660-Del		INPP5D	intron_variant	Other	-	C	0.27	18.92	27397079.00	3.25E-02	6.84E-02					
3:112324705-Del		CCDC80	intron_variant	Other	-	G	0.27	18.92	27397079.00	3.25E-02	6.84E-02					
4:26361434-Ins-2		RBPJ	intron_variant	Other	T	-	0.27	18.92	27397079.00	3.25E-02	6.84E-02					
4:100232929-Ins		ADH1B	intron_variant	Other	A	-	0.27	18.92	27397079.00	3.25E-02	6.84E-02					
5:133887961-Del		JADE2	intron_variant	Other	-	C	0.27	18.92	27397079.00	3.25E-02	6.84E-02					
10:69918731-Ins		MYPN	intron_variant	Other	CA	-	0.27	18.92	27397079.00	3.25E-02	6.84E-02					
12:81674982-Ins		PPFIA2,PPFIA2-AS	intron_variant	Other	T	-	0.27	18.92	27397079.00	3.25E-02	6.84E-02					
15:76032249-Ins-2		DNM1P35	splice_region_variant	Other	T	-	0.27	18.92	27397079.00	3.25E-02	6.84E-02					
1:117484227-Ins		PTGFRN	intron_variant	Other	TG	-	0.23	18.86	27397079.00	3.42E-02	6.84E-02					
4:140275066-Ins		NAA15	intron_variant	Other	T	-	0.23	18.86	27397079.00	3.42E-02	6.84E-02					
15:40565970-Ins		BUB1B-PAK6,PAK6	intron_variant	Other	A	-	0.23	18.86	27397079.00	3.42E-02	6.84E-02					
8:141756992-Del-2	rs369382658	PTK2	intron_variant	Other	-	TTT	0.15	18.90	23726566.41	3.53E-02	6.85E-02					
9:114147817-Del		ECPAS	intron_variant	Other	-	A	0.15	19.15	33554432.00	3.53E-02	6.85E-02					
19:17450545-Del-2		GTPBP3	intron_variant	Other	-	TTCT	0.15	19.15	33554432.00	3.53E-02	6.85E-02					
1:183083528-Ins		LAMC1	intron_variant	Other	GT	-	0.25	18.66	23726566.41	3.32E-02	6.85E-02					
12:1902766-SNV	rs2058111	CACNA2D4	3_prime_UTR_variant	Other	G	T	0.32	18.84	16777216.00	1.12E-03	7.48E-02	A	A	3.85	Benign	A
17:76547634-SNV	rs16971526	DNAH17	missense_variant	Missense	C	T	0.16	18.61	23726566.41	3.22E-02	7.58E-02	A	A	13.2	Benign	
12:96063948-Del	rs3833792	NTN4,PGAM1P5	intron_variant	Other	-	GTTA	0.33	1.10	0.59	3.12E-02	7.61E-02					
1:94998138-Del		F3	intron_variant	Other	-	T	0.33	19.01	27397079.00	2.97E-02	7.61E-02					
4:48549561-Ins		FRYL	intron_variant	Other	GT	-	0.33	19.01	27397079.00	2.97E-02	7.61E-02					
16:68380892-Ins		PRMT7	intron_variant	Other	TCC	-	0.33	19.01	27397079.00	2.97E-02	7.61E-02					
18:44683394-Ins		IER3IP1	intron_variant	Other	T	-	0.33	19.01	27397079.00	2.97E-02	7.61E-02					
12:53679654-Ins		ESPL1	intron_variant	Other	GT	-	0.22	18.66	23726566.41	3.11E-02	7.67E-02					
19:33467676-SNV	rs55853151	FAAP24	3_prime_UTR_variant	Other	A	G	0.22	18.66	23726566.41	3.11E-02	7.67E-02	A	A	0.627		A
3:130311398-SNV	rs73868925	COL6A6	missense_variant	Missense	T	C	0.16	18.61	23726566.41	3.22E-02	7.67E-02					
5:169810796-SNV	rs11739136	KCNIP1,KCNMB1	missense_variant	Missense	T	C	0.16	18.61	23726566.41	3.22E-02	7.67E-02					
3:48420835-Del	rs1226683388	FBXW12	intron_variant	Other	-	A	0.15	18.84	27397079.00	3.03E-02	7.68E-02	NA	A	0.15		
6:107097124-Del	rs3040665	QRSL1	intron_variant	Other	-	AC	0.25	18.59	21221686.14	2.30E-02	7.92E-02					
3:121258271-Ins		POLQ	intron_variant	Other	T	-	0.21	18.94	27397079.00	2.55E-02	7.98E-02					
7:122774381-Ins-2		SLC13A1	intron_variant	Other	T	-	0.21	18.94	27397079.00	2.55E-02	7.98E-02					
13:39453239-Del		FREM2	intron_variant	Other	-	A	0.21	18.94	27397079.00	2.55E-02	7.98E-02					
17:45376531-Ins		ITGB3	intron_variant	Other	T	-	0.21	18.94	27397079.00	2.55E-02	7.98E-02					
2:20179533-Del	rs767542195	WDR35	intron_variant	Other	-	TA	0.24	18.49	16777216.00	2.41E-02	8.02E-02	NA	NA			
3:155218297-Ins		PLCH1	intron_variant	Other	T	-	0.33	19.01	27397079.00	2.97E-02	8.02E-02					
5:149385920-Ins		HMGXB3	intron_variant	Other	GT	-	0.33	19.01	27397079.00	2.97E-02	8.02E-02					
19:9037996-Ins	rs1406767128	MUC16	intron_variant	Other	T	-	0.33	18.76	19372660.35	2.97E-02	8.02E-02	NA	A	2.32		
2:54849365-Ins-2		SPTBN1	intron_variant	Other	T	-	0.25	19.01	27397079.00	2.29E-02	8.02E-02					
4:134111466-Del		PCDH10	3_prime_UTR_variant	Other	-	G	0.16	18.92	27397079.00	2.37E-02	8.03E-02					
13:47279376-Ins	rs11483731	LRCH1	intron_variant	Other	-	T	0.32	19.01	17935598.34	8.09E-04	8.09E-02					
12:69995273-SNV	rs7200	CCT2	3_prime_UTR_variant	Other	C	T	0.19	18.89	27397079.00	2.75E-02	8.10E-02	A	A	12.4		A
16:86575216-Ins	rs933218369	MTHFSD	intron_variant	Other	T	-	0.19	18.89	27397079.00	2.75E-02	8.10E-02	NA	A	1.14		
2:56102253-Del		EFEMP1	intron_variant	Other	-	T	0.25	19.01	27397079.00	2.29E-02	8.17E-02					
8:95448623-Ins		FSBP,RAD54B	intron_variant	Other	TG	-	0.25	19.01	27397079.00	2.29E-02	8.17E-02					
11:117391736-Del	rs7936795	DSCAML1	intron_variant	Other	-	A	0.25	19.01	27397079.00	2.29E-02	8.17E-02	A	A	1.57		NA
19:53627440-Ins		ZNF415	intron_variant	Other	T	-	0.25	19.01	27397079.00	2.29E-02	8.17E-02					
1:103348627-Del	rs1397134786	COL11A1	intron_variant	Other	-	T	0.18	18.66	23726566.41	2.82E-02	8.18E-02	NA	A	5.51		
1:94487286-Del	rs55860151	ABCA4	intron_variant	Other	-	AC	0.17	18.66	23726566.41	2.72E-02	8.26E-02					
10:129904474-SNV	rs61738284	MKI67	missense_variant	Missense	A	G	0.17	18.66	23726566.41	2.72E-02	8.26E-02	A	A	1.17		
2:186604030-Ins-2		FSIP2,FSIP2-AS2	intron_variant	Other	T	-	0.20	18.66	23726566.41	2.95E-02	8.30E-02					
17:6554921-SNV	rs74504320	MED31	5_prime_UTR_variant	Other	G	A	0.20	18.66	23726566.41	2.95E-02	8.30E-02					
11:60774304-Del		CD6	intron_variant	Other	-	GAG	0.25	19.01	27397079.00	2.29E-02	8.80E-02					
12:57642314-Ins		STAC3	intron_variant	Other	T	-	0.25	19.01	27397079.00	2.29E-02	8.80E-02					
10:135339843-Del					-	T	0.25	19.46	33554432.00	2.29E-02	9.15E-02					
15:20862860-Ins					T	-	0.25	19.46	33554432.00	2.29E-02	9.15E-02					
1:44587669-Del		KLF17	intron_variant	Other	-	TA	0.25	19.46	33554432.00	2.29E-02	9.53E-02					
2:31557091-Ins	rs1279885680	XDH	downstream_gene_variant	Unknown	G	-	0.25	19.46	33554432.00	2.29E-02	9.53E-02	NA	NA			
2:192246057-Ins	rs768180538	MYO1B	intron_variant	Other	GT	-	0.25	19.46	33554432.00	2.29E-02	9.53E-02	A	A	8.23		NA
9:116080657-Ins		WDR31	intron_variant	Other	T	-	0.25	19.46	33554432.00	2.29E-02	9.53E-02					

(whites_continued2)

18:59174447-Ins		CDH20	intron_variant	Other	AGT	-	0.25	19.46	33554432.00	2.29E-02	9.53E-02					
3:121507372-Del	rs141690316	IQCB1	intron_variant	Other	-	TTTTTA	0.17	18.55	16777216.00	2.25E-02	1.05E-01					
10:102673063-SNV	rs3802725	SLF2	3_prime_UTR_variant	Other	T	G	0.36	1.17	0.59	1.97E-02	1.06E-01	A	A	20.2		NA
7:92977998-Ins		VPS50	intron_variant	Other	T	-	0.18	18.95	27397079.00	2.18E-02	1.07E-01					
2:25497685-Ins	rs367544298	DNMT3A	intron_variant	Other	C	-	0.35	-1.30	0.64	2.25E-02	1.07E-01	NA	A	4.29	Benign	
2:56606764-Ins		CCDC85A	intron_variant	Other	CT	-	0.20	19.01	27397079.00	1.96E-02	1.09E-01					
5:34888746-Del		TTC23L	intron_variant	Other	-	T	0.20	18.79	19372660.35	1.96E-02	1.09E-01					
6:166743628-Ins		SFT2D1	intron_variant	Other	CT	-	0.20	19.01	27397079.00	1.96E-02	1.09E-01					
3:171426433-Ins	rs1716887724	PLD1	intron_variant	Other	TC	-	0.18	18.95	27397079.00	2.18E-02	1.09E-01	NA	NA			
7:150813978-Ins	rs76473497	AGAP3	intron_variant	Other	G	-	0.23	18.60	21221686.14	2.09E-02	1.10E-01					
2:219146868-SNV	rs2292554	PNKD,TMBIM1	5_prime_UTR_variant	Other	C	T	0.35	1.18	0.60	2.17E-02	1.11E-01					
18:67727624-Del		RTTN	intron_variant	Other	-	GG	0.20	19.01	27397079.00	1.96E-02	1.19E-01					
1:192544816-Del	rs1017115045	RGS1	upstream_gene_variant	Unknown	-	G	0.30	19.12	27397079.00	1.92E-02	1.20E-01	NA	A	19.4		
3:183059200-Del		MCF2L2	intron_variant	Other	-	A	0.30	19.12	27397079.00	1.92E-02	1.20E-01					
4:85748220-Del		WDFY3	intron_variant	Other	-	C	0.30	19.12	27397079.00	1.92E-02	1.20E-01					
5:140254821-Ins	rs2055366681	4,PCDHA5,PCDH	splice_region_variant	Other	T	-	0.30	19.12	27397079.00	1.92E-02	1.20E-01	NA	A	15.6		
6:170844209-Del		PSMB1	3_prime_UTR_variant	Other	-	A	0.30	19.12	27397079.00	1.92E-02	1.20E-01					
7:103767477-Del		ORC5	intron_variant	Other	-	G	0.30	19.12	27397079.00	1.92E-02	1.20E-01					
16:57247659-Ins		RSPRY1	intron_variant	Other	T	-	0.30	19.12	27397079.00	1.92E-02	1.20E-01					
18:59959003-Del		RELCH	intron_variant	Other	-	T	0.30	19.12	27397079.00	1.92E-02	1.20E-01					
19:51412775-Del		KLK4	intron_variant	Other	-	G	0.30	19.56	27397079.00	5.47E-03	1.37E-01					
6:25652569-Del	rs3215471	SCGN	5_prime_UTR_variant	Other	-	G	0.40	-1.84	0.76	3.54E-03	1.42E-01	NA	A	4.15		
6:31829596-Ins		NEU1	intron_variant	Other	T	-	0.25	18.86	21221686.14	6.76E-03	1.50E-01					
16:75301624-Ins		BCAR1	intron_variant	Other	T	-	0.30	19.56	27397079.00	5.47E-03	1.56E-01					
7:107013040-Ins		COG5	frameshift_variant	LoF	T	-	0.20	18.79	23726566.41	1.80E-02	1.57E-01					
1:213069563-SNV	rs12567713	FLVCR1	3_prime_UTR_variant	Other	T	C	0.20	18.66	21221686.14	1.49E-02	1.57E-01	A	A	5.6	Benign	A
11:126147697-SNV	rs667627	FOXRED1	3_prime_UTR_variant	Other	A	G	0.20	18.66	21221686.14	1.49E-02	1.57E-01	A	A	1.17	Benign	A
17:7999589-SNV	rs3809882	ALOXE3	3_prime_UTR_variant	Other	G	T	0.48	1.04	0.48	1.89E-02	1.57E-01					
1:146767320-SNV	rs6537	CHD1L	3_prime_UTR_variant	Other	A	G	0.40	19.01	23726566.41	1.58E-02	1.58E-01	A	A	0.2		A
2:219509618-SNV	rs2230115	ZNF142	missense_variant	Missense	A	C	0.44	1.45	0.59	3.18E-03	1.59E-01					
1:204931531-Del	rs1317902600	NFASC	intron_variant	Other	-	C	0.22	19.56	33554432.00	1.68E-02	1.60E-01	NA	A	13		
1:235746949-Ins	rs1349130659	GNG4	intron_variant	Other	T	-	0.22	18.95	23726566.41	9.62E-03	1.60E-01	NA	A	8.21		
3:48929310-Ins		SLC25A20	intron_variant	Other	T	-	0.20	19.63	33554432.00	1.29E-02	1.61E-01					
12:55354821-Del		TESPA1	intron_variant	Other	-	C	0.20	19.63	33554432.00	1.29E-02	1.61E-01					
19:51465201-Ins	rs112376245	KLK6	intron_variant	Other	C	-	0.21	-1.49	0.69	1.77E-02	1.61E-01					
15:23258265-Del	rs199827219	GOLGA8IP	intron_variant	Other	TCTC	-	0.27	-1.35	0.62	1.16E-02	1.66E-01					
10:90534075-Ins	rs17112748	LIPN	intron_variant	Other	GT	-	0.31	19.01	23726566.41	1.09E-02	1.67E-01	A	A	4.88		NA
19:3543479-Del	rs34196068	C19orf71	intron_variant	Other	-	C	0.29	18.59		1.43E-02	1.68E-01					
16:71956594-Ins	rs3038732	IST1	intron_variant	Other	GTGTGG	-	0.24	18.79	21221686.14	9.36E-03	1.70E-01					
9:130457373-Ins					GAA	-	0.27	18.79	19372660.35	5.12E-03	1.71E-01					
1:205312767-SNV	rs7526603	KLHDC8A	5_prime_UTR_variant	Other	T	G	0.25	18.66	19372660.35	9.11E-03	1.82E-01					

AAs

Predictor	Identifier	Gene Names	Sequence Ontology	Effect	Minor Allele	Major Allele	MAF	Beta	SE	P-Value	FDR	TF	GnomAD	CADD	miRNA	
5:31409066-Ins		DROSHA	intron_variant	Other	T	-	0.14	18.77	27397079.00	3.74E-02	4.80E-02					
14:64449254-Del	rs199597838	SYNE2	intron_variant	Other	-	AAG	0.14	18.77	27397079.00	3.74E-02	4.80E-02					
18:2726350-Ins		SMCHD1	intron_variant	Other	T	-	0.14	18.77	27397079.00	3.74E-02	4.80E-02					
18:61261763-Del		SERPINB13	intron_variant	Other	-	T	0.16	18.79	27397079.00	3.69E-02	4.83E-02					
6:38749240-Del		DNAH8	intron_variant	Other	-	T	0.16	18.79	27397079.00	3.69E-02	4.86E-02					
9:38569472-Del	rs112902043				-	AG	0.16	18.79	27397079.00	3.69E-02	4.86E-02					
15:54630732-Ins		UNC13C	intron_variant	Other	AG	-	0.16	18.79	27397079.00	3.69E-02	4.86E-02					
12:41421525-Ins		CNTN1	intron_variant	Other	T	-	0.18	18.80	27397079.00	3.62E-02	4.91E-02					
22:25150663-Ins		PIWIL3	intron_variant	Other	T	-	0.18	18.80	27397079.00	3.62E-02	4.91E-02					
8:70539623-Del		SULF1	intron_variant	Other	-	C	0.13	19.12	33554432.00	3.56E-02	4.93E-02					
3:15498335-Del	rs141471889	COLQ	intron_variant	Other	-	TTCTTTTG	0.26	1.11	0.60	3.68E-02	4.96E-02					
3:8775923-Ins		CAV3	intron_variant	Other	A	-	0.13	19.12	33554432.00	3.56E-02	4.96E-02					
1:24137067-Ins-3		HMGCL	intron_variant	Other	T	-	0.20	18.83	27397079.00	3.54E-02	4.97E-02					
22:38042705-Ins		PDXP-DT,SH3BP1	intron_variant	Other	T	-	0.20	18.58	19372660.35	3.54E-02	4.97E-02					
1:156095195-Del	rs111616133	LMNA	intron_variant	Other	-	G	0.18	18.80	27397079.00	3.62E-02	4.98E-02					
1:174987485-Ins		MRPS14	intron_variant	Other	GT	-	0.14	19.01	33554432.00	4.99E-02	4.99E-02					
2:65481099-Del		ACTR2	intron_variant	Other	-	C	0.14	19.01	33554432.00	4.99E-02	4.99E-02					
10:81063685-Ins		ZMIZ1	intron_variant	Other	T	-	0.14	19.01	33554432.00	4.99E-02	4.99E-02					
14:75563692-Ins		NEK9	intron_variant	Other	GT	-	0.14	19.01	33554432.00	4.99E-02	4.99E-02					
18:77623561-Del	rs1979306039	KCNG2	upstream_gene_variant	Unknown	-	C	0.14	19.01	33554432.00	4.99E-02	4.99E-02					
1:47842518-Del		CMPK1	3_prime_UTR_variant	Other	-	T	0.19	18.89	27397079.00	2.75E-02	4.99E-02					
2:47602201-Ins		EPCAM	intron_variant	Other	T	-	0.19	18.89	27397079.00	2.75E-02	4.99E-02					
4:100336567-Ins		ADH7	intron_variant	Other	T	-	0.19	18.89	27397079.00	2.75E-02	4.99E-02					
5:41199711-Ins		C6	intron_variant	Other	GT	-	0.19	18.89	27397079.00	2.75E-02	4.99E-02					
5:127612084-Del		FBN2	intron_variant	Other	-	G	0.19	18.89	27397079.00	2.75E-02	4.99E-02					
9:79319596-Ins		PRUNE2	intron_variant	Other	GT	-	0.19	18.89	27397079.00	2.75E-02	4.99E-02					
3:64641615-Ins		ADAMTS9	intron_variant	Other	A	-	0.20	18.58	19372660.35	3.54E-02	5.05E-02					
18:72167381-Del		CNDP2	intron_variant	Other	-	T	0.15	19.15	33554432.00	3.53E-02	5.07E-02					
1:18704098-Del		IGSF21	intron_variant	Other	-	T	0.27	18.92	27397079.00	3.25E-02	5.08E-02					
1:156087782-Del		LMNA	intron_variant	Other	-	TTTTG	0.27	18.92	27397079.00	3.25E-02	5.08E-02					
5:58272398-Ins	rs1743851161	PDE4D	intron_variant	Other	A	-	0.27	18.92	27397079.00	3.25E-02	5.08E-02	NA	NA			
11:117969558-Ins		TMPRSS4	intron_variant	Other	T	-	0.27	18.92	27397079.00	3.25E-02	5.08E-02					
11:129772066-Ins		PRDM10	3_prime_UTR_variant	Other	GT	-	0.27	18.92	27397079.00	3.25E-02	5.08E-02					
6:31378229-Ins	rs56728307	MICA	intron_variant	Other	CCT	-	0.38	1.23	0.65	3.28E-02	5.09E-02					
1:156531520-Ins-2		IQGAP3	intron_variant	Other	T	-	0.30	18.80	27397079.00	4.83E-02	5.09E-02					
6:109796433-Ins-2	rs891382179	ZBTB24	intron_variant	Other	T	-	0.30	18.80	27397079.00	4.83E-02	5.09E-02	NA	A	8.62		
7:103767469-Del		ORC5	intron_variant	Other	-	C	0.30	18.80	27397079.00	4.83E-02	5.09E-02					
12:104131673-Ins	rs562780017	STAB2	intron_variant	Other	A	-	0.30	18.80	27397079.00	4.83E-02	5.09E-02	A	A	8.42		NA
15:72155046-Ins		MYO9A	intron_variant	Other	CA	-	0.30	18.80	27397079.00	4.83E-02	5.09E-02					
16:68729948-Ins		CDH3	intron_variant	Other	A	-	0.30	18.80	27397079.00	4.83E-02	5.09E-02					
1:40776148-Ins		COL9A2	intron_variant	Other	CA	-	0.15	19.15	33554432.00	3.53E-02	5.11E-02					
1:211923219-Ins	rs1657102044	LPGAT1	3_prime_UTR_variant	Other	T	-	0.15	19.15	33554432.00	3.53E-02	5.11E-02	NA	A	11.1		
5:170341297-Ins		RANBP17	intron_variant	Other	GA	-	0.15	19.15	33554432.00	3.53E-02	5.11E-02					
11:108186360-Ins		ATM,C11orf65	intron_variant	Other	CT	-	0.15	18.94	23726566.41	3.53E-02	5.11E-02					
12:2904179-Ins		FKBP4,ITFG2-AS1	5_prime_UTR_variant	Other	T	-	0.15	19.15	33554432.00	3.53E-02	5.11E-02					
12:123343235-Ins		HIP1R	intron_variant	Other	GT	-	0.15	18.90	23726566.41	3.53E-02	5.11E-02					
21:42817284-Ins		MX1	intron_variant	Other	T	-	0.15	19.15	33554432.00	3.53E-02	5.11E-02					
22:42271255-Ins		SREBF2	intron_variant	Other	T	-	0.15	19.15	33554432.00	3.53E-02	5.11E-02					
19:48184643-Del	rs58014830	BICRA	intron_variant	Other	G	-	0.29	-1.24	0.65	3.32E-02	5.11E-02					
2:30975767-Ins		CAPN13	intron_variant	Other	GT	-	0.14	19.01	33554432.00	4.99E-02	5.12E-02					
5:72157517-Ins		TNPO1	intron_variant	Other	T	-	0.14	19.01	33554432.00	4.99E-02	5.12E-02					
15:22482683-Ins					CT	-	0.14	18.76	23726566.41	4.99E-02	5.12E-02					
15:65871598-Ins		INTS14	3_prime_UTR_variant	Other	T	-	0.14	19.01	33554432.00	4.99E-02	5.12E-02					
1:29018312-Del		GMEB1	intron_variant	Other	-	T	0.33	19.01	27397079.00	2.97E-02	5.14E-02					
1:65332389-Del		JAK1	intron_variant	Other	-	G	0.33	19.01	27397079.00	2.97E-02	5.14E-02					
7:150693421-Ins-2		NOS3	intron_variant	Other	T	-	0.33	19.01	27397079.00	2.97E-02	5.14E-02					
13:78211387-Del-2	rs755741313	SCEL	intron_variant	Other	-	ATAT	0.33	19.01	27397079.00	2.97E-02	5.14E-02					
16:70995731-Ins		HYDIN	intron_variant	Other	T	-	0.33	19.01	27397079.00	2.97E-02	5.14E-02					
9:5068896-Ins		JAK2	intron_variant	Other	T	-	0.11	19.05	33554432.00	4.09E-02	5.15E-02					
12:131451185-Ins		ADGRD1	intron_variant	Other	AG	-	0.11	19.05	33554432.00	4.09E-02	5.15E-02					
1:12304296-Ins		VPS13D	splice_region_variant	Other	T	-	0.14	18.82	27397079.00	3.13E-02	5.15E-02					
7:137092746-Del	rs3217275	DGKI	splice_region_variant	Other	A	-	0.14	18.82	27397079.00	3.13E-02	5.15E-02					
16:67318608-Ins		PLEKHG4	splice_region_variant	Other	T	-	0.14	18.82	27397079.00	3.13E-02	5.15E-02					
2:30410171-Ins		SNORA10B	upstream_gene_variant	Unknown	CT	-	0.25	18.77	27397079.00	4.73E-02	5.15E-02					
2:241514213-Del		RNPEPL1	intron_variant	Other	-	C	0.25	18.77	27397079.00	4.73E-02	5.15E-02					
4:77065491-Del	rs77895217	NUP54	intron_variant	Other	-	AT	0.25	18.77	27397079.00	4.73E-02	5.15E-02					
7:21781417-Ins-2		DNAH11	intron_variant	Other	T	-	0.25	18.77	27397079.00	4.73E-02	5.15E-02					
17:19314860-Ins		RNF112	intron_variant	Other	T	-	0.25	18.77	27397079.00	4.73E-02	5.15E-02					
1:170508176-Ins		GORAB	intron_variant	Other	T	-	0.21	18.94	27397079.00	2.55E-02	5.16E-02					
1:184854065-Ins		NIBAN1	intron_variant	Other	A	-	0.21	18.94	27397079.00	2.55E-02	5.16E-02					
2:28069803-Ins-2		RBKS	intron_variant	Other	T	-	0.21	18.94	27397079.00	2.55E-02	5.16E-02					
2:165755375-Del		SLC38A11	intron_variant	Other	-	G	0.21	18.94	27397079.00	2.55E-02	5.16E-02					
2:197738542-Ins		PGAP1	intron_variant	Other	A	-	0.21	18.94	27397079.00	2.55E-02	5.16E-02					
3:4741740-Del	rs1559725145	ITPR1	intron_variant	Other	-	A	0.21	18.94	27397079.00	2.55E-02	5.16E-02	NA	A	3.58		
10:75863319-Ins-3		VCL	intron_variant	Other	T	-	0.21	18.94	27397079.00	2.55E-02	5.16E-02					
12:122818749-Del		CLIP1	intron_variant	Other	-	CTCA	0.21	18.94	27397079.00	2.55E-02	5.16E-02					
13:73321320-Del		BORA	intron_variant	Other	-	T	0.21	18.94	27397079.00	2.55E-02	5.16E-02					
18:67614751-Ins		CD226	5_prime_UTR_variant	Other	A	-	0.21	18.94	27397079.00	2.55E-02	5.16E-02					
19:50496360-Del		VRK3	intron_variant	Other	-	G	0.21	18.94	27397079.00	2.55E-02	5.16E-02					

(AAs_continued1)

19:55589357-Ins	rs965383698	EPS8L1	intron_variant	Other	T	-	0.21	18.94	27397079.00	2.55E-02	5.16E-02	NA	A	5.92		
8:106815856-SNV	rs6991211	ZFPM2,ZFPM2-AS1	3_prime_UTR_variant	Other	G	C	0.33	18.66	23726566.41	4.08E-02	5.20E-02	A	A	14.9		A
19:8495843-Ins		MARCHF2	intron_variant	Other	A	-	0.15	18.84	27397079.00	3.03E-02	5.21E-02					
11:73669218-Ins		DNAJB13	intron_variant	Other	GT	-	0.14	19.01	33554432.00	4.99E-02	5.23E-02					
17:12844580-Ins		ARHGAP44	intron_variant	Other	CA	-	0.13	19.07	33554432.00	4.18E-02	5.24E-02					
17:74684401-SNV	rs2286587	MXRA7	missense_variant	Missense	C	T	0.13	18.80	27397079.00	3.22E-02	5.25E-02					
18:43469684-Ins		EPG5	intron_variant	Other	T	-	0.13	18.81	27397079.00	2.65E-02	5.25E-02					
7:73483689-SNV	rs8326	ELN	3_prime_UTR_variant	Other	C	G	0.38	-0.97	0.49	3.12E-02	5.27E-02	A	A	0.662	Benign	A
17:3840695-Del	rs34866939	ATP2A3	intron_variant	Other	-	A	0.30	1.15	0.60	2.64E-02	5.28E-02	NA	A	1.37		
16:3594163-Del	rs60164526	NLRC3	intron_variant	Other	-	A	0.41	-1.15	0.58	3.10E-02	5.28E-02					
1:34049129-Ins		CSMD2	intron_variant	Other	T	-	0.22	19.12	33554432.00	4.58E-02	5.29E-02					
4:44712821-Ins		GNPDA2	intron_variant	Other	T	-	0.22	19.12	33554432.00	4.58E-02	5.29E-02					
11:8942745-Ins		C11orf16	intron_variant	Other	T	-	0.22	19.12	33554432.00	4.58E-02	5.29E-02					
12:51072319-Ins		DIP2B	intron_variant	Other	T	-	0.22	19.12	33554432.00	4.58E-02	5.29E-02					
12:57494010-Del		STAT6	intron_variant	Other	-	T	0.22	19.12	33554432.00	4.58E-02	5.29E-02					
14:21622974-Ins		OR5AU1	downstream_gene_variant	Unknown	CT	-	0.22	19.12	33554432.00	4.58E-02	5.29E-02					
18:55321012-Ins		ATP8B1,LOC1005055	intron_variant	Other	T	-	0.22	19.12	33554432.00	4.58E-02	5.29E-02					
6:79657194-Ins		PHIP	intron_variant	Other	T	-	0.19	18.89	27397079.00	2.75E-02	5.29E-02					
10:75855988-Del		VCL	intron_variant	Other	-	T	0.19	18.89	27397079.00	2.75E-02	5.29E-02					
22:42310078-Ins-2		SHISA8	intron_variant	Other	GT	-	0.19	18.89	27397079.00	2.75E-02	5.29E-02					
17:17249950-Ins	rs1243468245	NT5M	intron_variant	Other	T	-	0.25	18.77	27397079.00	4.73E-02	5.30E-02	NA	A	0.05		
5:177638835-Ins		PHYKPL	intron_variant	Other	T	-	0.12	19.01	33554432.00	4.72E-02	5.32E-02					
6:116431924-Ins		NT5DC1	intron_variant	Other	GT	-	0.12	19.01	33554432.00	4.72E-02	5.32E-02					
6:132199865-Ins		ENPP1	intron_variant	Other	A	-	0.12	18.84	23726566.41	4.72E-02	5.32E-02					
15:101827076-Ins		SNRPA1	intron_variant	Other	T	-	0.12	19.01	33554432.00	4.72E-02	5.32E-02					
2:31606871-Del	rs1216449429	XDH	intron_variant	Other	-	T	0.17	19.08	33554432.00	4.33E-02	5.35E-02	NA	A	0.22		
15:74327359-Ins		PML	3_prime_UTR_variant	Other	T	-	0.17	19.08	33554432.00	4.33E-02	5.35E-02					
5:146793053-Ins		DPYSL3	intron_variant	Other	T	-	0.15	18.72	27397079.00	4.50E-02	5.46E-02					
6:52764660-Ins		GSTA3	intron_variant	Other	GT	-	0.15	18.72	27397079.00	4.50E-02	5.46E-02					
7:21744896-Ins	rs375593250	DNAH11	intron_variant	Other	T	-	0.17	18.73	27397079.00	4.54E-02	5.47E-02	A	NA			NA
5:94749904-Ins	rs11418250	FAM81B	intron_variant	Other	T	-	0.14	18.75	27397079.00	4.47E-02	5.49E-02					
6:37281477-Del		TBC1D22B	intron_variant	Other	-	G	0.16	18.92	27397079.00	2.37E-02	5.75E-02					
7:74120881-Ins		GTF2I,LOC10192694	intron_variant	Other	AC	-	0.16	18.92	27397079.00	2.37E-02	5.75E-02					
11:95570806-Ins		MTMR2	intron_variant	Other	T	-	0.16	18.92	27397079.00	2.37E-02	5.75E-02					
17:38142763-Ins		PSMD3	intron_variant	Other	T	-	0.16	18.92	27397079.00	2.37E-02	5.75E-02					
20:39976084-Ins		LPIN3	intron_variant	Other	T	-	0.16	18.92	27397079.00	2.37E-02	5.75E-02					
20:40084337-Ins		CHD6	intron_variant	Other	GT	-	0.16	18.92	27397079.00	2.37E-02	5.75E-02					
1:179503108-Ins		AXDND1	intron_variant	Other	CA	-	0.14	18.64	19372660.35	2.52E-02	5.83E-02					
4:101386749-Ins		EMCN	intron_variant	Other	A	-	0.14	18.64	19372660.35	2.52E-02	5.90E-02					
4:106534506-Ins		ARHGEF38	intron_variant	Other	T	-	0.14	18.64	19372660.35	2.52E-02	5.90E-02					
4:144532776-Ins		FREM3	intron_variant	Other	A	-	0.14	18.89	27397079.00	2.52E-02	5.90E-02					
6:41566439-Del		FOXP4	intron_variant	Other	-	C	0.25	19.01	27397079.00	2.29E-02	6.00E-02					
10:105163119-Ins		PDCD11	intron_variant	Other	A	-	0.25	19.01	27397079.00	2.29E-02	6.08E-02					
17:61271290-Ins-2		TANC2	intron_variant	Other	T	-	0.25	19.01	27397079.00	2.29E-02	6.08E-02					
5:41201902-Del		C6	intron_variant	Other	-	T	0.25	19.01	27397079.00	2.29E-02	6.25E-02					
9:4575869-Ins		SLC1A1	intron_variant	Other	CT	-	0.25	19.46	33554432.00	2.29E-02	6.25E-02					
12:81015823-Del	rs149289370	PTPRQ	intron_variant	Other	-	C	0.25	19.46	33554432.00	2.29E-02	6.25E-02					
20:38774764-Ins	rs61140196				CTC	-	0.25	19.46	33554432.00	2.29E-02	6.25E-02					
1:9005719-Ins	rs569155900	CA6	upstream_gene_variant	Unknown	T	-	0.25	19.46	33554432.00	2.29E-02	6.62E-02	A	A	0.209		NA
1:77092934-Ins		ST6GALNAC3	intron_variant	Other	T	-	0.25	19.46	33554432.00	2.29E-02	6.62E-02					
2:43919600-Ins		PLEKHH2	intron_variant	Other	CT	-	0.25	19.46	33554432.00	2.29E-02	6.62E-02					
3:164704708-Ins	rs1717465572	SI	intron_variant	Other	CT	-	0.25	19.46	33554432.00	2.29E-02	6.62E-02	NA	A	2.56		
7:152550448-Del		ACTR3B	intron_variant	Other	-	G	0.25	19.46	33554432.00	2.29E-02	6.62E-02					
9:14398786-Ins		NFIB	5_prime_UTR_variant	Other	A	-	0.25	19.46	33554432.00	2.29E-02	6.62E-02					
9:74828655-Ins	rs1190818855	GDA	intron_variant	Other	T	-	0.25	19.46	33554432.00	2.29E-02	6.62E-02	NA	A	3.71		
11:121482058-Ins		SORL1	intron_variant	Other	ACA	-	0.25	19.46	33554432.00	2.29E-02	6.62E-02					
12:75693580-Ins		CAPS2	intron_variant	Other	T	-	0.25	19.46	33554432.00	2.29E-02	6.62E-02					
13:32923395-Ins-2		BRCA2	intron_variant	Other	T	-	0.25	19.46	33554432.00	2.29E-02	6.62E-02					
3:128864449-Ins		ISY1,ISY1-RAB43	intron_variant	Other	CT	-	0.25	19.46	33554432.00	2.29E-02	7.78E-02					
9:116764088-Ins		ZNF618	intron_variant	Other	T	-	0.25	19.46	33554432.00	2.29E-02	7.78E-02					
12:25185510-Ins		LOC645177	intron_variant	Other	A	-	0.25	19.46	33554432.00	2.29E-02	7.78E-02					
4:6066508-Ins		JAKMIP1	intron_variant	Other	T	-	0.15	18.96	27397079.00	1.96E-02	7.90E-02					
15:44951105-Ins		SPG11	intron_variant	Other	T	-	0.18	18.95	27397079.00	2.18E-02	7.99E-02					
17:7574340-Ins	rs1567542667	TP53	intron_variant	Other	T	-	0.18	18.95	27397079.00	2.18E-02	7.99E-02	NA	NA			
20:62378693-Ins	rs1199903552	ZBTB46	intron_variant	Other	A	-	0.18	18.95	27397079.00	2.18E-02	7.99E-02	NA	A	0.621		
6:31378226-Ins	rs200894869	MICA	intron_variant	Other	CCT	-	0.35	1.30	0.64	2.25E-02	8.07E-02					
1:175129682-Ins		KIAA0040	3_prime_UTR_variant	Other	T	-	0.20	19.01	27397079.00	1.96E-02	8.07E-02					
3:53708042-Ins		CACNA1D	intron_variant	Other	AC	-	0.20	19.01	27397079.00	1.96E-02	8.07E-02					
11:118015028-Del		SCN4B	intron_variant	Other	-	C	0.20	19.01	27397079.00	1.96E-02	8.07E-02					
16:5517383-Del					-	C	0.18	18.95	27397079.00	2.18E-02	8.47E-02					
1:20443106-Ins		PLA2G2D	intron_variant	Other	AC	-	0.17	19.10	23726566.41	2.17E-02	8.60E-02					
11:5291209-Ins		HBE1	upstream_gene_variant	Unknown	AC	-	0.20	19.01	27397079.00	1.96E-02	8.62E-02					
12:104406462-Ins		GLT8D2	intron_variant	Other	CT	-	0.20	19.01	27397079.00	1.96E-02	8.62E-02					
1:36068784-Ins-2		PSMB2	3_prime_UTR_variant	Other	T	-	0.20	19.01	27397079.00	1.96E-02	9.03E-02					
4:140624778-Del		MGST2	intron_variant	Other	-	G	0.20	19.01	27397079.00	1.96E-02	9.03E-02					
12:56962661-Ins		RBMS2	intron_variant	Other	TC	-	0.20	19.01	27397079.00	1.96E-02	9.03E-02					
19:21948412-Ins		ZNF100	intron_variant	Other	C	-	0.20	19.01	27397079.00	1.96E-02	9.03E-02					
2:26507566-Ins		HADHB	intron_variant	Other	T	-	0.15	19.41	33554432.00	1.78E-02	9.08E-02					
3:53783636-Del		CACNA1D	intron_variant	Other	-	C	0.15	19.41	33554432.00	1.78E-02	9.08E-02					
19:37101428-Ins		ZNF382	intron_variant	Other	T	-	0.15	19.41	33554432.00	1.78E-02	9.08E-02					

(AAs_continued2)

4:184626015-Ins		TRAPPC11	intron_variant	Other	GT	-	0.15	19.41	33554432.00	1.78E-02	9.86E-02					
5:71521792-Ins	rs1747883227	MRPS27	intron_variant	Other	GT	-	0.17	19.01	27397079.00	1.76E-02	1.00E-01	NA	NA			
16:18839297-Ins	rs2141190062	SMG1	intron_variant	Other	GT	-	0.17	19.01	27397079.00	1.76E-02	1.00E-01	NA	A	0.467		
17:37579508-Ins		MED1	intron_variant	Other	T	-	0.17	18.83	19372660.35	1.76E-02	1.07E-01					
2:237076795-SNV	rs140196177	GBX2	5_prime_UTR_variant	Other	A	G	0.29	18.61	16777216.00	1.73E-02	1.09E-01					
3:57269383-Ins	rs1579380468	APPL1	intron_variant	Other	T	-	0.22	19.34	23726566.41	1.68E-02	1.09E-01	NA	A	10		
7:27139792-Ins		HOTAIRM1	non_coding_exon_variant	Other	GT	-	0.22	19.56	33554432.00	1.68E-02	1.09E-01					
10:93600943-Del		TNKS2	intron_variant	Other	-	TT	0.22	19.56	33554432.00	1.68E-02	1.09E-01					
15:77271278-Ins					T	-	0.22	19.56	33554432.00	1.68E-02	1.09E-01					
20:32661159-Ins		RALY	intron_variant	Other	T	-	0.22	19.56	33554432.00	1.68E-02	1.09E-01					
2:231305222-Ins		SP100	5_prime_UTR_variant	Other	TG	-	0.30	19.56	27397079.00	5.47E-03	1.18E-01					
10:91479123-Ins-2		KIF20B	intron_variant	Other	T	-	0.21	19.41	27397079.00	5.17E-03	1.25E-01					
11:117886314-Ins-2		SMIM35	intron_variant	Other	T	-	0.16	19.05	27397079.00	1.44E-02	1.27E-01					
19:57988667-Ins	rs34678661	ZNF772	inframe_insertion	Missense	GCC	-	0.33	1.37	0.60	6.64E-03	1.29E-01					
3:39116114-Ins		WDR48	intron_variant	Other	T	-	0.22	19.56	33554432.00	1.68E-02	1.30E-01					
1:144917841-SNV	rs1698681	PDE4DIP	missense_variant	Missense	C	T	0.21	18.93	21221686.14	4.05E-03	1.31E-01					
11:48510654-SNV	rs7103557	OR4A47	missense_variant	Missense	C	A	0.21	18.93	21221686.14	4.05E-03	1.31E-01					
10:93593551-Ins		TNKS2	intron_variant	Other	T	-	0.22	19.12	23726566.41	4.73E-03	1.31E-01					
10:88556184-Ins	rs1589720265	BMPR1A	intron_variant	Other	AC	-	0.31	18.75		1.42E-02	1.31E-01	NA	NA			
12:120580211-Ins		GCN1	intron_variant	Other	GT	-	0.40	19.01	23726566.41	1.58E-02	1.33E-01					
9:97365939-Del		FBP1	intron_variant	Other	-	G	0.19	19.21	27397079.00	7.60E-03	1.34E-01					
3:16253968-Ins-2		GALNT15	intron_variant	Other	T	-	0.23	19.08	27397079.00	1.67E-02	1.35E-01					
1:150780608-Ins		CTSK	intron_variant	Other	T	-	0.19	18.84	23726566.41	1.41E-02	1.36E-01					
1:32689478-Ins		EIF3I	intron_variant	Other	GT	-	0.20	19.63	33554432.00	1.29E-02	1.39E-01					
14:91773656-Del		CCDC88C	intron_variant	Other	-	C	0.18	19.12	27397079.00	1.22E-02	1.39E-01					
12:23915548-Ins	rs71059921	SOX5	intron_variant	Other	-	A	0.38	19.35	27397079.00	1.36E-02	1.39E-01					
5:850774-Del	rs67067158	ZDHHC11	5_prime_UTR_variant	Other	-	G	0.18	18.64	16777216.00	1.11E-02	1.44E-01					
10:70099332-Del	rs148334349	HNRNPH3	intron_variant	Other	-	TA	0.18	18.64	16777216.00	1.11E-02	1.44E-01					
17:62130397-Ins		ERN1	intron_variant	Other	-	CT	0.46	-1.70	0.77	9.13E-03	1.48E-01					
1:92573371-Ins		BTBD8	intron_variant	Other	T	-	0.18	19.12	27397079.00	1.22E-02	1.48E-01					
1:213178828-Ins	rs1361391315	ANGEL2	intron_variant	Other	AC	-	0.18	18.64	16777216.00	1.11E-02	1.66E-01	NA	A	5.16		
1:19972671-Del	rs142481240	MICOS10-NBL1,NBL	intron_variant	Other	-	A	0.27	19.63	27397079.00	3.77E-03	1.83E-01					
1:155707000-Del		DAP3	intron_variant	Other	-	T	0.27	19.63	27397079.00	3.77E-03	1.83E-01					
3:15498334-Del	rs1297523120	COLQ	intron_variant	Other	-	TTTCTTTTG	0.23	18.96	21221686.14	3.70E-03	3.59E-01					
11:129772172-SNV	rs1540408	PRDM10	3_prime_UTR_variant	Other	C	T	0.22	18.87	21221686.14	2.81E-03	5.45E-01	A	A	3.53		A

Als

Gene Names	Sequence Ontology	Effect	Minor Allele	Major Allele	MAF	Beta	SE	P-Value	FDR	TF	GnomAD	CADD	ClinVar	miRNA
SLC5A7	missense_variant	Missense	G	A	0.15	20.21	23726566.41	2.66E-05	2.18E-03					
SLC17A5	3_prime_UTR_variant	Other	C	T	0.15	20.21	23726566.41	2.66E-05	2.18E-03	A	A	5.64	Benign	A
PNPT1	intron_variant	Other	CT	-	0.12	19.98	33554432.00	3.56E-03	1.77E-02					
FASTKD2	intron_variant	Other	GT	-	0.12	19.98	33554432.00	3.56E-03	1.77E-02					
MFSD10	intron_variant	Other	T	-	0.12	19.98	33554432.00	3.56E-03	1.77E-02					
GARNL3	intron_variant	Other	-	T	0.12	19.98	33554432.00	3.56E-03	1.77E-02					
CRTC2	intron_variant	Other	-	G	0.13	19.94	33554432.00	4.10E-03	1.82E-02					
PPP2R5B	intron_variant	Other	GT	-	0.13	19.94	33554432.00	4.10E-03	1.82E-02					
PNKP	intron_variant	Other	T	-	0.13	19.94	33554432.00	4.10E-03	1.82E-02					
RBM20	intron_variant	Other	T	-	0.11	20.01	33554432.00	3.11E-03	1.82E-02					
MRE11	3_prime_UTR_variant	Other	T	-	0.11	20.01	33554432.00	3.11E-03	1.82E-02					
RBL2	intron_variant	Other	-	G	0.11	20.01	33554432.00	3.11E-03	1.82E-02					
RPN2	intron_variant	Other	-	TT	0.11	20.01	33554432.00	3.11E-03	1.82E-02	NA	A	0.9		NA
PCP4	upstream_gene_variant	Unknown	GT	-	0.11	20.01	33554432.00	3.11E-03	1.82E-02					
OR4C46	missense_variant	Missense	T	C	0.26	1.62	0.64	3.81E-03	1.84E-02					
MMUT	3_prime_UTR_variant	Other	T	C	0.21	1.50	0.57	4.54E-03	1.86E-02	A	A	1.31	Benign	A
INMT,INMT-MINDY4	missense_variant	Missense	G	A	0.21	1.50	0.57	4.54E-03	1.86E-02					
PNPLA6	5_prime_UTR_variant	Other	C	T	0.21	1.50	0.57	4.54E-03	1.86E-02					
IL1B	intron_variant	Other	-	GT	0.13	19.65	23726566.41	4.78E-03	1.87E-02					
GOSR1	intron_variant	Other	T	-	0.13	19.65	23726566.41	4.78E-03	1.87E-02					
LILRB2	missense_variant	Missense	T	C	0.26	1.93	0.66	4.56E-04	1.87E-02					
NAV2,NAV2-AS2	missense_variant	Missense	T	G	0.19	2.22	0.76	3.59E-04	1.96E-02					
ZAR1	missense_variant	Missense	T	A	0.33	1.53	0.61	3.52E-03	1.99E-02					
AGAP2	intron_variant	Other	C	-	0.21	2.14	0.76	6.17E-04	2.02E-02	NA	A	3.83		NA
SETBP1	3_prime_UTR_variant	Other	-	TT	0.21	1.87	0.78	5.49E-03	2.09E-02	NA	A	0.783		A
FAM189A2	intron_variant	Other	CCGGCCGCAC	-	0.22	1.73	0.67	2.95E-03	2.10E-02	NA	A	6.71		NA
LYST	splice_region_variant	Other	A	-	0.32	1.56	0.61	2.86E-03	2.13E-02	NA	A	1.92	Benign/Likely benign	NA
SLC4A8	intron_variant	Other	CT	-	0.08	20.21	33554432.00	1.47E-03	2.19E-02					
WNK1	intron_variant	Other	T	-	0.10	20.08	33554432.00	2.44E-03	2.22E-02					
DNAAF9	intron_variant	Other	CT	-	0.10	20.08	33554432.00	2.44E-03	2.22E-02					
CYP1B1	3_prime_UTR_variant	Other	C	T	0.32	1.56	0.61	2.86E-03	2.23E-02	A	A	4.52	Benign	A
PDSS1	intron_variant	Other	ATT	-	0.32	1.56	0.61	2.86E-03	2.23E-02					
MAN2C1	intron_variant	Other	GCCCAGCCCAT/	-	0.36	1.42	0.60	6.14E-03	2.24E-02					
MAN2C1	intron_variant	Other	CCAGCCCATACC	-	0.36	1.42	0.60	6.14E-03	2.24E-02					
MYL6B	intron_variant	Other	AAAA	-	0.23	1.45	0.57	6.35E-03	2.26E-02	NA	A	7.17		NA
MMP27	intron_variant	Other	C	-	0.09	19.97	23726566.41	1.96E-03	2.30E-02					
MMP27	intron_variant	Other	T	-	0.09	19.97	23726566.41	1.96E-03	2.30E-02					
A2ML1	missense_variant	Missense	T	C	0.22	1.84	0.67	1.41E-03	2.31E-02					
PELATON	missense_variant	Missense	T	C	0.19	1.84	0.67	1.41E-03	2.31E-02					
XRN2	intron_variant	Other	T	-	0.10	20.10	33554432.00	2.18E-03	2.39E-02					
MUC19	missense_variant	Missense	T	C	0.09	19.91	23726566.41	1.77E-03	2.42E-02					
MTHFR	missense_variant	Missense	A	G	0.18	1.87	0.67	1.19E-03	2.44E-02					
GPC6	3_prime_UTR_variant	Other	T	C	0.18	1.87	0.67	1.19E-03	2.44E-02	A	A	14.1	Benign	A
APC	intron_variant	Other	T	-	0.32	1.56	0.61	2.86E-03	2.47E-02	NA	A	1.12		NA
DENND5B	missense_variant	Missense	T	C	0.21	1.76	0.67	2.42E-03	2.48E-02					
PKHD1L1	intron_variant	Other	-	T	0.08	19.61	33554432.00	7.26E-03	2.53E-02					
AVIL	intron_variant	Other	A	-	0.24	1.79	0.78	8.15E-03	2.62E-02	NA	A	2.53	Benign	NA
AKR1E2	intron_variant	Other	TTTTT	-	0.30	1.45	0.62	8.39E-03	2.64E-02					
CLEC9A	intron_variant	Other	CT	-	0.11	19.63	33554432.00	8.09E-03	2.65E-02					
CCDC38	intron_variant	Other	GT	-	0.11	19.63	33554432.00	8.09E-03	2.65E-02					
YEATS4	intron_variant	Other	T	-	0.30	1.50	0.64	7.96E-03	2.72E-02	NA	A	0.73		NA
NDRG1	intron_variant	Other	ACACACAC	-	0.25	1.75	0.78	1.01E-02	2.77E-02	NA	A	3.77		NA
FANCG	5_prime_UTR_variant	Other	A	-	0.07	19.49	33554432.00	1.01E-02	2.80E-02					
COX10	3_prime_UTR_variant	Other	-	CT	0.07	19.49	33554432.00	1.01E-02	2.80E-02	NA	A	0.04	Benign	A
PDE4DIP	5_prime_UTR_variant	Other	T	C	0.07	19.51	33554432.00	9.26E-03	2.81E-02					
NRXN1	UTR_premature_start_codon_ga	Missense	T	C	0.07	19.51	33554432.00	9.26E-03	2.81E-02	A	A	5.18	Benign	
			-	A	0.31	1.42	0.62	9.96E-03	2.87E-02					
CRTAP	3_prime_UTR_variant	Other	C	T	0.14	1.52	0.65	1.07E-02	2.88E-02	A	A	5.88	Benign	A
PRPS1L1	upstream_gene_variant	Unknown	-	CT	0.31	1.42	0.62	9.96E-03	2.92E-02					
TAFA4	5_prime_UTR_variant	Other	G	T	0.09	19.53	33554432.00	9.78E-03	2.92E-02	A	A	7.09		NA
BRD2	intron_variant	Other	-	T	0.32	1.39	0.62	1.19E-02	3.15E-02					
ANO2	intron_variant	Other	T	-	0.10	19.46	33554432.00	1.23E-02	3.19E-02					
CDHGA11,PCDHGA1:	missense_variant	Missense	G	A	0.18	1.87	0.67	1.19E-03	3.26E-02					
C5orf49	5_prime_UTR_variant	Other	A	C	0.11	19.39	33554432.00	1.58E-02	3.92E-02	A	A	0.99		NA
VANGL1	3_prime_UTR_variant	Other	-	A	0.19	1.35	0.59	1.66E-02	3.94E-02	NA	A	0.59	Likely benign	A
ALDH18A1	3_prime_UTR_variant	Other	G	A	0.19	1.35	0.59	1.66E-02	3.94E-02	A	A	4.73	Benign	A
SCFD2	intron_variant	Other	-	T	0.14	1.79	0.80	1.54E-02	3.94E-02	NA	A	0.114		NA
PLCD4,ZNF142	3_prime_UTR_variant	Other	A	G	0.24	1.45	0.66	1.58E-02	3.97E-02	A	A	0.379		A
CACHD1	intron_variant	Other	T	-	0.07	19.94	47453132.81	3.24E-02	4.00E-02					
ZRANB2	intron_variant	Other	-	AAG	0.07	19.94	47453132.81	3.24E-02	4.00E-02					
PNPT1	intron_variant	Other	-	G	0.07	19.94	47453132.81	3.24E-02	4.00E-02					
LRRFIP2	intron_variant	Other	T	-	0.07	19.94	47453132.81	3.24E-02	4.00E-02					
ARAP2	intron_variant	Other	T	-	0.07	19.94	47453132.81	3.24E-02	4.00E-02					
KCTD16	5_prime_UTR_variant	Other	T	-	0.07	19.94	47453132.81	3.24E-02	4.00E-02					
GEMIN5	intron_variant	Other	T	-	0.07	19.94	47453132.81	3.24E-02	4.00E-02					
LPAL2	non_coding_exon_variant	Other	GT	-	0.07	19.94	47453132.81	3.24E-02	4.00E-02	A	A	0.076		NA
ABCB1	intron_variant	Other	TC	-	0.07	19.94	47453132.81	3.24E-02	4.00E-02					
GAPVD1	intron_variant	Other	GT	-	0.07	19.94	47453132.81	3.24E-02	4.00E-02					
ZNF248	3_prime_UTR_variant	Other	T	-	0.07	19.94	47453132.81	3.24E-02	4.00E-02					

(AIs_continued1)

CLPB	intron_variant	Other	GT	-	0.07	19.94	47453132.81	3.24E-02	4.00E-02					
			GT	-	0.07	19.94	47453132.81	3.24E-02	4.00E-02					
NTAN1,PDXDC1	intron_variant	Other	T	-	0.07	19.94	47453132.81	3.24E-02	4.00E-02					
LAMA1	intron_variant	Other	GT	-	0.07	19.94	47453132.81	3.24E-02	4.00E-02	NA	A	0.514		NA
ASXL3	intron_variant	Other	TG	-	0.07	19.94	47453132.81	3.24E-02	4.00E-02					
ZSWIM4	intron_variant	Other	GT	-	0.07	19.94	47453132.81	3.24E-02	4.00E-02					
PNPLA5	intron_variant	Other	CT	-	0.07	19.94	47453132.81	3.24E-02	4.00E-02					
KALRN	intron_variant	Other	-	T	0.14	1.76	0.80	1.71E-02	4.02E-02					
PYGO1	5_prime_UTR_variant	Other	T	C	0.27	1.21	0.53	1.66E-02	4.05E-02	A	A	14.2		NA
BAGE2	3_prime_UTR_variant	Other	A	-	0.17	1.45	0.72	3.53E-02	4.08E-02	NA	A	19.3		NA
ABCD3	intron_variant	Other	-	G	0.07	19.65	33554432.00	3.53E-02	4.10E-02					
ATP11B	intron_variant	Other	GT	-	0.07	19.65	33554432.00	3.53E-02	4.10E-02					
GCLC	3_prime_UTR_variant	Other	T	-	0.07	19.65	33554432.00	3.53E-02	4.10E-02					
RIMS1	intron_variant	Other	CT	-	0.07	19.65	33554432.00	3.53E-02	4.10E-02					
PRXL2A	intron_variant	Other	A	-	0.07	19.65	33554432.00	3.53E-02	4.10E-02					
LGR4	intron_variant	Other	TG	-	0.07	19.65	33554432.00	3.53E-02	4.10E-02					
MYH11	intron_variant	Other	-	TC	0.07	19.65	33554432.00	3.53E-02	4.10E-02	NA	A	0.514		NA
GRK3	intron_variant	Other	T	-	0.07	19.65	33554432.00	3.53E-02	4.10E-02					
RELN	intron_variant	Other	T	-	0.21	1.27	0.59	2.47E-02	4.22E-02					
PLOD1	intron_variant	Other	T	-	0.05	20.08	47453132.81	2.44E-02	4.22E-02					
AGL	intron_variant	Other	T	-	0.05	20.08	47453132.81	2.44E-02	4.22E-02					
LRP2	intron_variant	Other	-	A	0.05	20.08	47453132.81	2.44E-02	4.22E-02					
SSBP2	intron_variant	Other	GT	-	0.05	20.08	47453132.81	2.44E-02	4.22E-02					
HSD17B4	intron_variant	Other	AT	-	0.05	20.08	47453132.81	2.44E-02	4.22E-02					
EYS	intron_variant	Other	GT	-	0.05	20.08	47453132.81	2.44E-02	4.22E-02					
ZC2HC1B	intron_variant	Other	-	T	0.05	20.08	47453132.81	2.44E-02	4.22E-02					
LMBR1	intron_variant	Other	-	C	0.05	20.08	47453132.81	2.44E-02	4.22E-02					
ERICH5	intron_variant	Other	-	G	0.05	20.08	47453132.81	2.44E-02	4.22E-02	NA	A	5.38		NA
KTN1	intron_variant	Other	A	-	0.05	20.08	47453132.81	2.44E-02	4.22E-02					
LCMT1	intron_variant	Other	T	-	0.05	20.08	47453132.81	2.44E-02	4.22E-02					
SPAG16	intron_variant	Other	GT	-	0.05	19.97	33554432.00	2.17E-02	4.24E-02	NA	NA			
SUMF1	intron_variant	Other	T	-	0.05	19.97	33554432.00	2.17E-02	4.24E-02					
SLC17A5	intron_variant	Other	CT	-	0.05	19.97	33554432.00	2.17E-02	4.24E-02					
DLG2	intron_variant	Other	T	-	0.05	19.97	33554432.00	2.17E-02	4.24E-02					
ARHGAP9	intron_variant	Other	T	-	0.05	19.97	33554432.00	2.17E-02	4.24E-02					
SLX4	intron_variant	Other	C	-	0.05	19.97	33554432.00	2.17E-02	4.24E-02					
ACACA	intron_variant	Other	T	-	0.05	19.97	33554432.00	2.17E-02	4.24E-02					
POMT1	intron_variant	Other	-	CACTTCCTCTAAC	0.22	1.13	0.55	3.70E-02	4.25E-02					
SH3TC2	3_prime_UTR_variant	Other	A	G	0.29	1.10	0.51	2.53E-02	4.28E-02	A	A	0.314	Benign	NA
PRCC	intron_variant	Other	T	-	0.06	19.98	47453132.81	3.00E-02	4.28E-02	NA	NA			
ADD1	intron_variant	Other	-	A	0.06	19.98	47453132.81	3.00E-02	4.28E-02					
TMEM14C	intron_variant	Other	GT	-	0.06	19.98	47453132.81	3.00E-02	4.28E-02					
C7orf61	5_prime_UTR_variant	Other	AC	-	0.06	19.98	47453132.81	3.00E-02	4.28E-02					
CAPZA2	intron_variant	Other	TG	-	0.06	19.98	47453132.81	3.00E-02	4.28E-02					
RBM28	intron_variant	Other	CT	-	0.06	19.98	47453132.81	3.00E-02	4.28E-02					
XPO7	intron_variant	Other	TG	-	0.06	19.98	47453132.81	3.00E-02	4.28E-02					
LRP4	intron_variant	Other	-	A	0.06	19.98	47453132.81	3.00E-02	4.28E-02					
CPT1A	intron_variant	Other	GT	-	0.06	19.98	47453132.81	3.00E-02	4.28E-02					
EML5	intron_variant	Other	T	-	0.06	19.98	47453132.81	3.00E-02	4.28E-02					
RYR3	intron_variant	Other	T	-	0.06	19.98	47453132.81	3.00E-02	4.28E-02					
ZNF519	3_prime_UTR_variant	Other	TG	-	0.06	19.98	47453132.81	3.00E-02	4.28E-02					
NEGR1	intron_variant	Other	GT	-	0.05	19.91	33554432.00	2.06E-02	4.38E-02					
LRPPRC	intron_variant	Other	-	G	0.05	19.91	33554432.00	2.06E-02	4.38E-02					
COL4A4	intron_variant	Other	TC	-	0.05	19.91	33554432.00	2.06E-02	4.38E-02					
KCNAB1	splice_region_variant	Other	T	-	0.05	19.91	33554432.00	2.06E-02	4.38E-02					
LOC101928008,SBF2	intron_variant	Other	T	-	0.05	19.91	33554432.00	2.06E-02	4.38E-02					
IFT20	intron_variant	Other	T	-	0.05	19.91	33554432.00	2.06E-02	4.38E-02					
BCR	intron_variant	Other	T	-	0.05	19.91	33554432.00	2.06E-02	4.38E-02	NA	NA			
CISD1	intron_variant	Other	T	-	0.05	19.76	47453132.81	3.99E-02	4.39E-02					
NAALADL1	intron_variant	Other	TC	-	0.05	19.76	47453132.81	3.99E-02	4.39E-02					
TK2	3_prime_UTR_variant	Other	CT	-	0.05	19.76	47453132.81	3.99E-02	4.39E-02					
SLC35B1	intron_variant	Other	-	G	0.05	19.76	47453132.81	3.99E-02	4.39E-02	NA	A	4.21		
ARHGAP28	intron_variant	Other	T	-	0.05	19.76	47453132.81	3.99E-02	4.39E-02					
EDEM1	intron_variant	Other	GT	-	0.06	20.01	47453132.81	2.79E-02	4.44E-02					
LAMA2	intron_variant	Other	T	-	0.06	20.01	47453132.81	2.79E-02	4.44E-02	NA	A	3.74		
PRKN	intron_variant	Other	T	-	0.06	20.01	47453132.81	2.79E-02	4.44E-02					
PRKN	intron_variant	Other	T	-	0.06	20.01	47453132.81	2.79E-02	4.44E-02					
HBP1	intron_variant	Other	TC	-	0.06	20.01	47453132.81	2.79E-02	4.44E-02					
DLST	intron_variant	Other	GT	-	0.06	20.01	47453132.81	2.79E-02	4.44E-02					
SORCS3	intron_variant	Other	-	G	0.18	1.42	0.72	3.91E-02	4.45E-02					
BIN1	5_prime_UTR_variant	Other	-	C	0.09	19.76	47453132.81	4.74E-02	4.77E-02					
CACNA1D	intron_variant	Other	A	-	0.09	19.76	47453132.81	4.74E-02	4.77E-02					
ANAPC4	intron_variant	Other	AC	-	0.09	19.76	47453132.81	4.74E-02	4.77E-02					
CCDC158	intron_variant	Other	T	-	0.09	19.76	47453132.81	4.74E-02	4.77E-02					
PPP2CA	intron_variant	Other	T	-	0.09	19.76	47453132.81	4.74E-02	4.77E-02					
CASP1	3_prime_UTR_variant	Other	G	A	0.09	19.76	47453132.81	4.74E-02	4.77E-02	A	A	1.12		NA
RAD52	3_prime_UTR_variant	Other	T	C	0.09	19.76	47453132.81	4.74E-02	4.77E-02	A	A	3.32		NA
OAS3	intron_variant	Other	T	-	0.09	19.76	47453132.81	4.74E-02	4.77E-02					
LOC102723692,XYLT1	intron_variant	Other	CT	-	0.09	19.76	47453132.81	4.74E-02	4.77E-02					
ARHGAP17	intron_variant	Other	GT	-	0.09	19.76	47453132.81	4.74E-02	4.77E-02					
ZNF225	intron_variant	Other	C	-	0.09	19.76	47453132.81	4.74E-02	4.77E-02					
SHANK1	intron_variant	Other	T	-	0.09	19.76	47453132.81	4.74E-02	4.77E-02					
MRPL4	3_prime_UTR_variant	Other	T	C	0.24	1.07	0.55	4.78E-02	4.78E-02	A	A	1.53		A
KCNH2	intron_variant	Other	-	AAC	0.11	1.32	0.69	4.63E-02	5.03E-02					
KCNH2	intron_variant	Other	-	AAT	0.11	1.32	0.69	4.63E-02	5.03E-02					
OR4C46	missense_variant	Missense	A	G	0.26	3.91	0.78	3.00E-03	5.19E-02	A	A	22.9		

key
A = available
NA = not available

Supplementary Table 4. The top 200 significant variants

The table shows the top 200 significant variants (3’ or 5’ UTRs, missense, or intronic variants), whose frequencies differed significantly between tumor vs. benign tissues. The functionality was assessed with GnomAD, CADD and ClinVar. However, for White and African Americans only a few remained after the Bonferroni correction, whereas ~68 variants in Native Americans showed FDR p values between 0.039 and 0.0022.

Red highlighted text indicates the mutation (SNP) creates the binding sites for both MiRNA and transcription factor while the yellow highlighted text is the binding site for either transcription factor or miRNA.

MiRNA and TFs_Whites

Predictor	Identifier	Gene Names	Sequence Ontology	Effect	Minor Allele	Major Allele	miRNA	Function	Score	P-value	TF	Function	Score	P-value SNP impact
12:76442907-SNV	rs11180815	NAP1L1	3_prime_UTR_variant	Other	C	T	hsa-miR-649	Gain	25.19	0.02	SOX18	Gain	1.935	2.35E-05
12:76442907-SNV	rs11180815	NAP1L1	3_prime_UTR_variant	Other	C	T	hsa-miR-490-3p	Gain	22.69	0.02	RAD21	Gain	3.524	1.14E-04
12:76442907-SNV	rs11180815	NAP1L1	3_prime_UTR_variant	Other	C	T	hsa-miR-494-3p	Gain	21.1	0.02	SPDEF	Gain	3.496	2.74E-04
12:76442907-SNV	rs11180815	NAP1L1	3_prime_UTR_variant	Other	C	T					ELK4	Gain	2.992	3.84E-04
12:76442907-SNV	rs11180815	NAP1L1	3_prime_UTR_variant	Other	C	T					GATA	Gain	2.336	5.07E-04
12:76442907-SNV	rs11180815	NAP1L1	3_prime_UTR_variant	Other	C	T					SREBF1	Gain	4.053	8.90E-04
12:76442907-SNV	rs11180815	NAP1L1	3_prime_UTR_variant	Other	C	T					NKX2-1	loss	-3.449	9.74E-04
14:21990772-Ins	rs1018987788	SALL2	3_prime_UTR_variant	Other	T	-	hsa-miR-9986	Gain	22.47	0.01				
14:21990772-Ins	rs1018987788	SALL2	3_prime_UTR_variant	Other	T	-	hsa-miR-1294	Gain	23.97	0.01				
14:21990772-Ins	rs1018987788	SALL2	3_prime_UTR_variant	Other	T	-	hsa-miR-4677-3p	Gain	23	0.01				
14:21990772-Ins	rs1018987788	SALL2	3_prime_UTR_variant	Other	T	-	hsa-miR-4316	Gain	23.01	0.02				
14:21990772-Ins	rs1018987788	SALL2	3_prime_UTR_variant	Other	T	-	hsa-miR-4679	Gain	23.53	0.04				
14:21990772-Ins	rs1018987788	SALL2	3_prime_UTR_variant	Other	T	-	hsa-miR-3689a-3p	loss	23.1	0.01				
14:21990772-Ins	rs1018987788	SALL2	3_prime_UTR_variant	Other	T	-	hsa-miR-1273h-5p	loss	23.1	0.01				
14:21990772-Ins	rs1018987788	SALL2	3_prime_UTR_variant	Other	T	-	hsa-miR-30c-1-3p	loss	25.08	0.03				
14:21990772-Ins	rs1018987788	SALL2	3_prime_UTR_variant	Other	T	-	hsa-miR-3192-5p	loss	23	0.01				
14:21990772-Ins	rs1018987788	SALL2	3_prime_UTR_variant	Other	T	-	hsa-miR-4646-5p	loss	23.53	0.05				
14:21990772-Ins	rs1018987788	SALL2	3_prime_UTR_variant	Other	T	-	hsa-miR-6851-5p	loss	20.56	0.02				
14:21990772-Ins	rs1018987788	SALL2	3_prime_UTR_variant	Other	T	-	hsa-miR-7106-5p	loss	22.01	0.04				
14:21990772-Ins	rs1018987788	SALL2	3_prime_UTR_variant	Other	T	-	hsa-miR-30c-2-3p	loss	24.58	0.03				
14:21990772-Ins	rs1018987788	SALL2	3_prime_UTR_variant	Other	T	-	hsa-miR-6788-5p	loss	25.58	0.03				
14:21990772-Ins	rs1018987788	SALL2	3_prime_UTR_variant	Other	T	-	hsa-miR-326	loss	22.65	0.05				
14:21990772-Ins	rs1018987788	SALL2	3_prime_UTR_variant	Other	T	-	hsa-miR-619-5p	loss	23.03	0.05				
14:21990772-Ins	rs1018987788	SALL2	3_prime_UTR_variant	Other	T	-	hsa-miR-3689d	loss	20.56	0.02				
15:45445690-Del	rs758869498	DUOX1	intron_variant	Other	-	GAATGTGTGTGTGT					SOX1	loss	-2.018	4.00E-04
15:45445690-Del	rs758869498	DUOX1	intron_variant	Other	-	GAATGTGTGTGTGT					FOXO1	Gain	3.857	4.06E-04
15:45445690-Del	rs758869498	DUOX1	intron_variant	Other	-	GAATGTGTGTGTGT					POU3F3	Gain	3.483	6.84E-04
15:45445690-Del	rs758869498	DUOX1	intron_variant	Other	-	GAATGTGTGTGTGT					E2F4	loss	-2.897	9.07E-04
15:45445690-Del	rs758869498	DUOX1	intron_variant	Other	-	GAATGTGTGTGTGT					HDAC2	Gain	3.076	9.71E-04
12:1902766-SNV	rs2058111	CACNA2D4	3_prime_UTR_variant	Other	G	T	hsa-miR-2113	Gain	26.43	0.02	ZNF410	Gain	4.743	1.58E-04
12:1902766-SNV	rs2058111	CACNA2D4	3_prime_UTR_variant	Other	G	T	hsa-miR-1233-5p	Gain	21.45	0.05	RUNX	Gain	3.318	8.47E-04
12:1902766-SNV	rs2058111	CACNA2D4	3_prime_UTR_variant	Other	G	T	hsa-miR-6778-5p	Gain	22.45	0.05	NR5A1	Gain	2.933	9.11E-04
12:1902766-SNV	rs2058111	CACNA2D4	3_prime_UTR_variant	Other	G	T	hsa-miR-4769-5p	Gain	23.4	0.02	IKZF1	loss	-4.736	1.04E-03
12:1902766-SNV	rs2058111	CACNA2D4	3_prime_UTR_variant	Other	G	T	hsa-miR-5010-3p	Gain	21.93	0.02	PBX	Gain	3.401	1.57E-03
12:1902766-SNV	rs2058111	CACNA2D4	3_prime_UTR_variant	Other	G	T	hsa-miR-4654	Gain	21.4	0.02				
12:1902766-SNV	rs2058111	CACNA2D4	3_prime_UTR_variant	Other	G	T	hsa-miR-4648	Gain	21.95	0.05				
12:1902766-SNV	rs2058111	CACNA2D4	3_prime_UTR_variant	Other	G	T	hsa-miR-129-5p	loss	23.43	0.03				
12:1902766-SNV	rs2058111	CACNA2D4	3_prime_UTR_variant	Other	G	T	hsa-miR-6513-5p	loss	20.9	0.03				
12:1902766-SNV	rs2058111	CACNA2D4	3_prime_UTR_variant	Other	G	T	hsa-miR-9983-3p	loss	22.43	0.03				
12:1902766-SNV	rs2058111	CACNA2D4	3_prime_UTR_variant	Other	G	T	hsa-miR-5003-3p	loss	20.36	0.01				
19:33467676-SNV	rs55853151	FAAP24	3_prime_UTR_variant	Other	A	G	hsa-miR-221-5p	Gain	22.01	0.02	TCF4	Gain	10.101	1.13E-06
19:33467676-SNV	rs55853151	FAAP24	3_prime_UTR_variant	Other	A	G	hsa-miR-3622b-3p	Gain	22.42	0.01	EBOX	Gain	10.202	1.55E-06
19:33467676-SNV	rs55853151	FAAP24	3_prime_UTR_variant	Other	A	G	hsa-miR-8073	Gain	21.51	0.02	MTF1	loss	-7.915	2.14E-06
19:33467676-SNV	rs55853151	FAAP24	3_prime_UTR_variant	Other	A	G	hsa-miR-3190-5p	Gain	21.56	0.02	ATF3	loss	-5.717	1.12E-05
19:33467676-SNV	rs55853151	FAAP24	3_prime_UTR_variant	Other	A	G	hsa-miR-619-3p	Gain	23.91	0.01	ELF1	loss	-2.703	4.10E-05
19:33467676-SNV	rs55853151	FAAP24	3_prime_UTR_variant	Other	A	G	hsa-miR-3622a-3p	Gain	24.42	0.01				
19:33467676-SNV	rs55853151	FAAP24	3_prime_UTR_variant	Other	A	G	hsa-miR-328-3p	Gain	20.99	0.04				
19:33467676-SNV	rs55853151	FAAP24	3_prime_UTR_variant	Other	A	G	hsa-miR-7851-3p	Gain	23.41	0.01				
19:33467676-SNV	rs55853151	FAAP24	3_prime_UTR_variant	Other	A	G	hsa-miR-3922-3p	Gain	23.06	0.02				
19:33467676-SNV	rs55853151	FAAP24	3_prime_UTR_variant	Other	A	G	hsa-miR-3176	Gain	21.56	0.02				
19:33467676-SNV	rs55853151	FAAP24	3_prime_UTR_variant	Other	A	G	hsa-miR-6075	loss	21.54	0.01				
19:33467676-SNV	rs55853151	FAAP24	3_prime_UTR_variant	Other	A	G	hsa-miR-6850-3p	loss	19.56	0.02				
19:33467676-SNV	rs55853151	FAAP24	3_prime_UTR_variant	Other	A	G	hsa-miR-10392-3p	loss	20.49	0.02				
12:69995273-SNV	rs7200	CCT2	3_prime_UTR_variant	Other	C	T	hsa-miR-5197-5p	Gain	22.03	0.03	YY1	Gain	3.083	2.19E-04
12:69995273-SNV	rs7200	CCT2	3_prime_UTR_variant	Other	C	T	hsa-miR-183-5p	Gain	24.59	0.01	IRF9	loss	-4.326	2.23E-04
12:69995273-SNV	rs7200	CCT2	3_prime_UTR_variant	Other	C	T	hsa-miR-513b-3p	Gain	21.03	0.02	CREB3L1	loss	-5.985	3.81E-04
12:69995273-SNV	rs7200	CCT2	3_prime_UTR_variant	Other	C	T	hsa-miR-425-5p	loss	23.09	0.02	SIX4	loss	-3.512	5.45E-04
12:69995273-SNV	rs7200	CCT2	3_prime_UTR_variant	Other	C	T	hsa-miR-602	loss	19.78	0.01	SP1	Gain	3.219	5.47E-04
12:69995273-SNV	rs7200	CCT2	3_prime_UTR_variant	Other	C	T					IRF8	loss	-2.583	5.52E-04
12:69995273-SNV	rs7200	CCT2	3_prime_UTR_variant	Other	C	T					E2F1	Gain	3.555	6.20E-04
11:117391736-Del	rs7936795	DSCAML1	intron_variant	Other	-	A					RUNX	Gain	7.267	8.85E-06
11:117391736-Del	rs7936795	DSCAML1	intron_variant	Other	-	A					YY1	Gain	8.691	4.07E-06
11:117391736-Del	rs7936795	DSCAML1	intron_variant	Other	-	A					TFCP2	Gain	11.073	1.09E-06
11:117391736-Del	rs7936795	DSCAML1	intron_variant	Other	-	A					CEBP	Gain	13.235	1.23E-05
11:117391736-Del	rs7936795	DSCAML1	intron_variant	Other	-	A					FOXC1	loss	-3.894	1.18E-03
2:192246057-Ins	rs768180538	MYO1B	intron_variant	Other	GT	-					MYOD1	Gain	12.687	1.80E-07
2:192246057-Ins	rs768180538	MYO1B	intron_variant	Other	GT	-					EP300	loss	-7.863	1.48E-05
2:192246057-Ins	rs768180538	MYO1B	intron_variant	Other	GT	-					ESRRA	loss	-5.582	7.29E-05
2:192246057-Ins	rs768180538	MYO1B	intron_variant	Other	GT	-					CREB3L2	loss	-3.417	9.52E-05
10:102673063-SNV	rs3802725	SLF2	3_prime_UTR_variant	Other	T	G					NFE2	loss	-8.821	6.00E-07
10:102673063-SNV	rs3802725	SLF2	3_prime_UTR_variant	Other	T	G					TEAD2	loss	-3.54	1.21E-04
10:102673063-SNV	rs3802725	SLF2	3_prime_UTR_variant	Other	T	G					CTCF	loss	-4.569	3.65E-04
1:213069563-SNV	rs12567713	FLVCR1	3_prime_UTR_variant	Other	T	C	hsa-miR-4789-5p	Gain	23.21	0.02	FOXA1	Gain	2.868	1.09E-04
1:213069563-SNV	rs12567713	FLVCR1	3_prime_UTR_variant	Other	T	C	hsa-let-7b-3p	Gain	21.22	0.01	SRY	Gain	4.086	4.76E-04
1:213069563-SNV	rs12567713	FLVCR1	3_prime_UTR_variant	Other	T	C	hsa-let-7a-3p	Gain	21.22	0.01	ZNF410	loss	-3.444	2.17E-03
1:213069563-SNV	rs12567713	FLVCR1	3_prime_UTR_variant	Other	T	C	hsa-miR-8066	Gain	20.75	0.02				
1:213069563-SNV	rs12567713	FLVCR1	3_prime_UTR_variant	Other	T	C	hsa-let-7f-2-3p	Gain	21.21	0.02				
1:213069563-SNV	rs12567713	FLVCR1	3_prime_UTR_variant	Other	T	C	hsa-let-7f-1-3p	Gain	21.22	0.01				
1:213069563-SNV	rs12567713	FLVCR1	3_prime_UTR_variant	Other	T	C	hsa-miR-1185-1-3p	Gain	21.71	0.02				
1:213069563-SNV	rs12567713	FLVCR1	3_prime_UTR_variant	Other	T	C	hsa-miR-1185-2-3p	Gain	21.71	0.02				
1:213069563-SNV	rs12567713	FLVCR1	3_prime_UTR_variant	Other	T	C	hsa-miR-4666a-3p	Gain	26.11	0.03				
1:213069563-SNV	rs12567713	FLVCR1	3_prime_UTR_variant	Other	T	C	hsa-miR-98-3p	Gain	22.72	0.01				
11:126147697-SNV	rs667627	FOXRED1	3_prime_UTR_variant	Other	A	G	hsa-miR-4261	Gain	23.32	0.04	NR3C1	loss	-6.541	9.36E-05
11:126147697-SNV	rs667627	FOXRED1	3_prime_UTR_variant	Other	A	G	hsa-miR-548g-3p	Gain	19.27	0.05	BDP1	loss	-5.849	5.15E-05
11:126147697-SNV	rs667627	FOXRED1	3_prime_UTR_variant	Other	A	G	hsa-miR-147b-5p	Gain	22.32	0.04	MYF	Gain	1.399	1.65E-04
11:126147697-SNV	rs667627	FOXRED1	3_prime_UTR_variant	Other	A	G	hsa-miR-1185-5p	Gain	20.32	0.02				
11:126147697-SNV	rs667627	FOXRED1	3_prime_UTR_variant	Other	A	G	hsa-miR-4496	Gain	22.38	0.01				
11:126147697-SNV	rs667627	FOXRED1	3_prime_UTR_variant	Other	A	G	hsa-miR-4738-3p	Gain	22.27	0.03				
11:126147697-SNV	rs667627	FOXRED1	3_prime_UTR_variant	Other	A	G	hsa-miR-3679-5p	Gain	23.32	0.02				
11:126147697-SNV	rs667627	FOXRED1	3_prime_UTR_variant	Other	A	G	hsa-miR-1197	loss	21.88	0.01				
11:126147697-SNV	rs667627	FOXRED1	3_prime_UTR_variant	Other	A	G	hsa-miR-5004-5p	loss	20.82	0.03				
11:126147697-SNV	rs667627	FOXRED1	3_prime_UTR_variant	Other	A	G	hsa-miR-3117-5p	loss	22.81	0.03				
11:126147697-SNV	rs667627	FOXRED1	3_prime_UTR_variant	Other	A	G	hsa-miR-6757-3p	loss	19.25	0.02				
1:146767320-SNV	rs6537	CHD1L	3_prime_UTR_variant	Other	A	G	hsa-miR-510-3p	Gain	23.19	0.01	FOXQ1	loss	-3.237	4.26E-04
1:146767320-SNV	rs6537	CHD1L	3_prime_UTR_variant	Other	A	G	hsa-miR-548g-3p	loss	24.05	0.04	SOX10	loss	-2.769	5.85E-04
1:146767320-SNV	rs6537	CHD1L	3_prime_UTR_variant	Other	A	G					MEF2A	Gain	2.151	1.02E-03
1:146767320-SNV	rs6537	CHD1L	3_prime_UTR_variant	Other	A	G					VSX2	Gain	2.497	1.27E-03
10:90534075-Ins	rs17112748	LIPN	intron_variant	Other	GT	-					HNF4A	Gain	4.943	8.16E-05
10:90534075-Ins	rs17112748	LIPN	intron_variant	Other	GT	-					RARA	Gain	6.589	4.50E-07
10:90534075-Ins	rs17112748	LIPN	intron_variant	Other	GT	-					NR2F6	Gain	7.658	5.09E-06
10:90534075-Ins	rs17112748	LIPN	intron_variant	Other	GT	-					GMEB2	loss	-4.741	3.10E-04

MiRNA and TFs_AAs

Predictor	Identifier	Gene Names	Sequence Ontology	Effect	Minor Allele	Major Allele	miRNA	Function	Score	P-value	TF	Function	Score	lue SNP im
12:104131673-Ins	rs562780017	STAB2	intron_variant	Other	A	-					ESRRA	loss	-5.863	6.73E-05
12:104131673-Ins	rs562780017	STAB2	intron_variant	Other	A	-					ARID5B	Gain	2.842	1.56E-03
12:104131673-Ins	rs562780017	STAB2	intron_variant	Other	A	-					ETS	Gain	2.238	1.84E-03
12:104131673-Ins	rs562780017	STAB2	intron_variant	Other	A	-					GLI	Gain	2.677	1.98E-03
12:104131673-Ins	rs562780017	STAB2	intron_variant	Other	A	-					IRF	Gain	3.127	2.76E-03
12:104131673-Ins	rs562780017	STAB2	intron_variant	Other	A	-					SPIC	Gain	3.129	3.73E-03
12:104131673-Ins	rs562780017	STAB2	intron_variant	Other	A	-					SOX8	Gain	2.404	4.26E-03
12:104131673-Ins	rs562780017	STAB2	intron_variant	Other	A	-					BCL	loss	-2.639	4.47E-03
12:104131673-Ins	rs562780017	STAB2	intron_variant	Other	A	-					HINFP	loss	-3.811	5.61E-03
8:106815856-SNV	rs6991211	ZFPM2,ZFPM2-AS	3_prime_UTR_variant	Other	G	C	hsa-miR-4717-3p	Gain	21.62	0.02	AP1	Gain	8.204	9.06E-06
8:106815856-SNV	rs6991211	ZFPM2,ZFPM2-AS	3_prime_UTR_variant	Other	G	C	hsa-miR-576-3p	loss	22.07	0.02	RUNX	Gain	3.367	1.83E-05
8:106815856-SNV	rs6991211	ZFPM2,ZFPM2-AS	3_prime_UTR_variant	Other	G	C	hsa-miR-1245b-3p	loss	23.12	0.02	FOXO1	Gain	6.671	4.38E-05
8:106815856-SNV	rs6991211	ZFPM2,ZFPM2-AS	3_prime_UTR_variant	Other	G	C	hsa-miR-3171	loss	20.12	0.01	ZNF75A	Gain	5.533	7.76E-05
8:106815856-SNV	rs6991211	ZFPM2,ZFPM2-AS	3_prime_UTR_variant	Other	G	C	hsa-miR-488-5p	loss	23.67	0.03	EGR1	Gain	3.395	1.74E-04
8:106815856-SNV	rs6991211	ZFPM2,ZFPM2-AS	3_prime_UTR_variant	Other	G	C					TP53	Gain	4.619	1.76E-04
8:106815856-SNV	rs6991211	ZFPM2,ZFPM2-AS	3_prime_UTR_variant	Other	G	C					GLI2	Gain	3.394	3.77E-04
7:73483689-SNV	rs8326	ELN	3_prime_UTR_variant	Other	C	G	hsa-miR-105-3p	Gain	21.99	0.04	ETS1	Gain	4.347	3.32E-03
7:73483689-SNV	rs8326	ELN	3_prime_UTR_variant	Other	C	G	hsa-miR-29c-5p	loss	23.84	0.05	E2F8	Gain	2.311	6.53E-03
7:73483689-SNV	rs8326	ELN	3_prime_UTR_variant	Other	C	G					PAX5	Gain	1.914	6.63E-03
7:73483689-SNV	rs8326	ELN	3_prime_UTR_variant	Other	C	G					BHLHE23	Gain	3.268	8.11E-03
7:73483689-SNV	rs8326	ELN	3_prime_UTR_variant	Other	C	G					OLIG2	Gain	2.937	8.25E-03
7:73483689-SNV	rs8326	ELN	3_prime_UTR_variant	Other	C	G					FOXK1	Gain	2.054	8.55E-03
7:73483689-SNV	rs8326	ELN	3_prime_UTR_variant	Other	C	G					PBX1	Gain	2.946	9.09E-03
7:21744896-Ins	rs375593250	DNAH11	intron_variant	Other	T	-					EGR1	loss	-2.785	9.74E-04
7:21744896-Ins	rs375593250	DNAH11	intron_variant	Other	T	-					MYC	loss	-3.696	9.81E-04
7:21744896-Ins	rs375593250	DNAH11	intron_variant	Other	T	-					TAL1	Gain	2.771	1.17E-03
7:21744896-Ins	rs375593250	DNAH11	intron_variant	Other	T	-					MAX	loss	-2.475	1.30E-03
7:21744896-Ins	rs375593250	DNAH11	intron_variant	Other	T	-					CTCF	Gain	2.65	1.93E-03
7:21744896-Ins	rs375593250	DNAH11	intron_variant	Other	T	-					GATA3	Gain	3.912	3.74E-03
1:9005719-Ins	rs569155900	CA6	upstream_gene_variant	Unknown	T	-					IRF7	loss	-12.78	9.00E-08
1:9005719-Ins	rs569155900	CA6	upstream_gene_variant	Unknown	T	-					ELK4	loss	-10.386	3.35E-06
1:9005719-Ins	rs569155900	CA6	upstream_gene_variant	Unknown	T	-					FLI1	loss	-8.79	5.33E-06
1:9005719-Ins	rs569155900	CA6	upstream_gene_variant	Unknown	T	-					ERF	loss	-8.2	8.80E-06
1:9005719-Ins	rs569155900	CA6	upstream_gene_variant	Unknown	T	-					TATA	loss	-6.936	2.23E-05
1:9005719-Ins	rs569155900	CA6	upstream_gene_variant	Unknown	T	-					ETV2	loss	-6.527	5.06E-05
11:129772172-SNV	rs1540408	PRDM10	3_prime_UTR_variant	Other	C	T	hsa-miR-4462	Gain	23.48	0.02	MYC	Gain	7.283	1.39E-04
11:129772172-SNV	rs1540408	PRDM10	3_prime_UTR_variant	Other	C	T					ESRRA	Gain	5.155	1.67E-04
11:129772172-SNV	rs1540408	PRDM10	3_prime_UTR_variant	Other	C	T					SMC3	Gain	5.357	2.09E-04
11:129772172-SNV	rs1540408	PRDM10	3_prime_UTR_variant	Other	C	T					USF	Gain	3.823	2.84E-04
11:129772172-SNV	rs1540408	PRDM10	3_prime_UTR_variant	Other	C	T					HES1	Gain	3.351	5.65E-04
11:129772172-SNV	rs1540408	PRDM10	3_prime_UTR_variant	Other	C	T					AR	loss	-3.395	1.12E-03
11:129772172-SNV	rs1540408	PRDM10	3_prime_UTR_variant	Other	C	T					BRCA1	Gain	3.603	1.17E-03
11:129772172-SNV	rs1540408	PRDM10	3_prime_UTR_variant	Other	C	T					CTCF	Gain	3.059	1.73E-03
11:129772172-SNV	rs1540408	PRDM10	3_prime_UTR_variant	Other	C	T					CREB1	loss	-3.12	1.73E-03

MiRNA and TFs_AIs

Predictor	Identifier	Gene Names	Sequence Ontology	Effect	Minor Allele	Major Allele	miRNA	Function	Score	P-value	TF	Function	Score	P-value SNP impact
6:74304607-SNV	rs3734517	SLC17A5	3_prime_UTR_variant	Other	C	T	hsa-miR-4643	Gain	25.71	0.07	SOX17	loss	-4.218	2.13E-05
6:74304607-SNV	rs3734517	SLC17A5	3_prime_UTR_variant	Other	C	T	hsa-miR-466	Gain	22.7	0.02	TEAD3	loss	-2.521	6.94E-04
6:74304607-SNV	rs3734517	SLC17A5	3_prime_UTR_variant	Other	C	T	hsa-miR-4528	Gain	21.66	0.04	TBX21	Gain	3.267	7.39E-04
6:74304607-SNV	rs3734517	SLC17A5	3_prime_UTR_variant	Other	C	T	hsa-miR-323a-3p	Gain	21.67	0.07	EGR	Gain	2.449	9.28E-04
6:74304607-SNV	rs3734517	SLC17A5	3_prime_UTR_variant	Other	C	T	hsa-miR-4789-3p	Gain	23.21	0.07	TBR1	Gain	3.504	1.50E-03
6:74304607-SNV	rs3734517	SLC17A5	3_prime_UTR_variant	Other	C	T	hsa-miR-5580-3p	loss	22.71	0.05	ZSCAN4	Gain	3.435	1.57E-03
6:74304607-SNV	rs3734517	SLC17A5	3_prime_UTR_variant	Other	C	T	hsa-miR-656-3p	loss	22.69	0.02	CREB3	Gain	4.993	1.78E-03
6:74304607-SNV	rs3734517	SLC17A5	3_prime_UTR_variant	Other	C	T	hsa-miR-1279	loss	23.76	0.05				
6:74304607-SNV	rs3734517	SLC17A5	3_prime_UTR_variant	Other	C	T	hsa-miR-4795-3p	loss	21.63	0.02				
6:49398115-SNV	rs9381784	MMUT	3_prime_UTR_variant	Other	T	C	hsa-miR-7157-5p	Gain	21	0.08	NANOG	Gain	7.684	4.15E-06
6:49398115-SNV	rs9381784	MMUT	3_prime_UTR_variant	Other	T	C	hsa-miR-4310	Gain	19.5	0.08	FOXN1	loss	-4.503	1.09E-05
6:49398115-SNV	rs9381784	MMUT	3_prime_UTR_variant	Other	T	C					EGR1	loss	-4.042	1.21E-04
6:49398115-SNV	rs9381784	MMUT	3_prime_UTR_variant	Other	T	C					HEY1	loss	-5.16	2.25E-04
6:49398115-SNV	rs9381784	MMUT	3_prime_UTR_variant	Other	T	C					ZBTB33	loss	-4.45	3.86E-04
6:49398115-SNV	rs9381784	MMUT	3_prime_UTR_variant	Other	T	C					TFAP2	loss	-4.195	4.37E-04
6:49398115-SNV	rs9381784	MMUT	3_prime_UTR_variant	Other	T	C					RREB1	loss	-1.982	4.85E-04
6:49398115-SNV	rs9381784	MMUT	3_prime_UTR_variant	Other	T	C					BCL	loss	-2.886	5.92E-04
18:42646635-Del-2	rs34125334	SETBP1	3_prime_UTR_variant	Other	-	TT	hsa-miR-877-3p	Gain	1.8	0.01				
2:38296890-SNV	rs2855658	CYP1B1	3_prime_UTR_variant	Other	C	T	hsa-miR-4801	Gain	22.18	0.01	CEBPB	Gain	12.483	1.67E-06
2:38296890-SNV	rs2855658	CYP1B1	3_prime_UTR_variant	Other	C	T	hsa-miR-4457	Gain	21.28	0.01	SP2	Gain	6.161	1.14E-04
2:38296890-SNV	rs2855658	CYP1B1	3_prime_UTR_variant	Other	C	T	hsa-miR-125b-2-3p	Gain	21.33	0.02	HES1	Gain	3.923	2.04E-04
2:38296890-SNV	rs2855658	CYP1B1	3_prime_UTR_variant	Other	C	T	hsa-miR-4731-3p	Gain	21.68	0.01	SRY	loss	-4.498	4.07E-04
2:38296890-SNV	rs2855658	CYP1B1	3_prime_UTR_variant	Other	C	T	hsa-let-7a-3p	loss	23.13	0.03	AP1	Gain	2.824	7.67E-04
2:38296890-SNV	rs2855658	CYP1B1	3_prime_UTR_variant	Other	C	T	hsa-miR-4666a-3p	loss	23.25	0.07	TCF12	Gain	3.183	9.50E-04
2:38296890-SNV	rs2855658	CYP1B1	3_prime_UTR_variant	Other	C	T	hsa-let-7b-3p	loss	23.13	0.03	NR4A2	Gain	2.872	1.15E-03
2:38296890-SNV	rs2855658	CYP1B1	3_prime_UTR_variant	Other	C	T	hsa-miR-4795-3p	loss	20.21	0.04				
2:38296890-SNV	rs2855658	CYP1B1	3_prime_UTR_variant	Other	C	T	hsa-let-7f-1-3p	loss	23.63	0.03				
2:38296890-SNV	rs2855658	CYP1B1	3_prime_UTR_variant	Other	C	T	hsa-miR-381-3p	loss	22.18	0.01				
2:38296890-SNV	rs2855658	CYP1B1	3_prime_UTR_variant	Other	C	T	hsa-miR-300	loss	22.18	0.01				
2:38296890-SNV	rs2855658	CYP1B1	3_prime_UTR_variant	Other	C	T	hsa-miR-98-3p	loss	23.63	0.03				
13:95056768-SNV	rs1951797	GPC6	3_prime_UTR_variant	Other	T	C	hsa-miR-6802-3p	Gain	22.5	0.03	MZF1	Gain	7.221	9.37E-05
13:95056768-SNV	rs1951797	GPC6	3_prime_UTR_variant	Other	T	C	hsa-miR-34b-3p	Gain	20.4	0.01	HNF1B	Gain	2.233	1.89E-04
13:95056768-SNV	rs1951797	GPC6	3_prime_UTR_variant	Other	T	C	hsa-miR-500b-3p	Gain	24.95	0.01	NFY	Gain	5.201	3.31E-04
13:95056768-SNV	rs1951797	GPC6	3_prime_UTR_variant	Other	T	C	hsa-miR-6884-3p	Gain	21.55	0.01	PBX3	Gain	4.182	5.91E-04
13:95056768-SNV	rs1951797	GPC6	3_prime_UTR_variant	Other	T	C	hsa-miR-197-3p	Gain	21	0.02	GFI1	Gain	3.23	8.19E-04
13:95056768-SNV	rs1951797	GPC6	3_prime_UTR_variant	Other	T	C	hsa-miR-660-5p	Gain	23.52	0.01	GLIS1	loss	-3.022	9.54E-04
17:14111361-Del	rs397763766	COX10	3_prime_UTR_variant	Other	-	CT	hsa-miR-4690-5p	Gain	21.36	0.02				
17:14111361-Del	rs397763766	COX10	3_prime_UTR_variant	Other	-	CT	hsa-miR-6165	Gain	20.84	0.01				
17:14111361-Del	rs397763766	COX10	3_prime_UTR_variant	Other	-	CT	hsa-miR-6510-5p	Gain	21.36	0.02				
17:14111361-Del	rs397763766	COX10	3_prime_UTR_variant	Other	-	CT	hsa-miR-1286	Gain	21.51	0.01				
17:14111361-Del	rs397763766	COX10	3_prime_UTR_variant	Other	-	CT	hsa-miR-4433a-3p	Gain	21.84	0.01				
17:14111361-Del	rs397763766	COX10	3_prime_UTR_variant	Other	-	CT	hsa-miR-4768-3p	Gain	22.39	0.02				
17:14111361-Del	rs397763766	COX10	3_prime_UTR_variant	Other	-	CT	hsa-miR-4722-5p	Gain	21.52	0.01				
17:14111361-Del	rs397763766	COX10	3_prime_UTR_variant	Other	-	CT	hsa-miR-3150b-3p	loss	23.37	0.03				
17:14111361-Del	rs397763766	COX10	3_prime_UTR_variant	Other	-	CT	hsa-miR-4784	loss	23.37	0.03				
17:14111361-Del	rs397763766	COX10	3_prime_UTR_variant	Other	-	CT	hsa-miR-4257	loss	20.92	0.03				
17:14111361-Del	rs397763766	COX10	3_prime_UTR_variant	Other	-	CT	hsa-miR-6847-5p	loss	21.39	0.01				
17:14111361-Del	rs397763766	COX10	3_prime_UTR_variant	Other	-	CT	hsa-miR-3916	loss	22.4	0.03				
17:14111361-Del	rs397763766	COX10	3_prime_UTR_variant	Other	-	CT	hsa-miR-6859-5p	loss	21.9	0.03				
17:14111361-Del	rs397763766	COX10	3_prime_UTR_variant	Other	-	CT	hsa-miR-2467-3p	loss	19.55	0.01				
17:14111361-Del	rs397763766	COX10	3_prime_UTR_variant	Other	-	CT	hsa-miR-3125	loss	21.9	0.03				
3:33186356-SNV	rs1127898	CRTAP	3_prime_UTR_variant	Other	C	T	hsa-miR-122b-3p	loss	23.46	0.01	PAX5	Gain	8.701	6.50E-06
3:33186356-SNV	rs1127898	CRTAP	3_prime_UTR_variant	Other	C	T	hsa-miR-140-3p	loss	18.58	0.02	PGR	loss	-3.53	3.76E-04
3:33186356-SNV	rs1127898	CRTAP	3_prime_UTR_variant	Other	C	T	hsa-miR-4773	loss	20.96	0.06	RHOXF1	loss	-4.147	3.80E-04
3:33186356-SNV	rs1127898	CRTAP	3_prime_UTR_variant	Other	C	T	hsa-miR-21-3p	loss	23.96	0.01	PLAG1	Gain	2.455	6.53E-04
3:33186356-SNV	rs1127898	CRTAP	3_prime_UTR_variant	Other	C	T					ZBTB33	Gain	3.015	9.12E-04
3:33186356-SNV	rs1127898	CRTAP	3_prime_UTR_variant	Other	C	T					CACBP	Gain	1.546	1.41E-03
3:68934434-SNV	rs4855535	TAFA4	5_prime_UTR_variant	Other	G	T					HF1H3B	loss	-2.183	2.72E-06
3:68934434-SNV	rs4855535	TAFA4	5_prime_UTR_variant	Other	G	T					NFIC	Gain	6.738	4.28E-05
3:68934434-SNV	rs4855535	TAFA4	5_prime_UTR_variant	Other	G	T					E2F1	Gain	3.884	7.30E-05
3:68934434-SNV	rs4855535	TAFA4	5_prime_UTR_variant	Other	G	T					TATA	Gain	5.328	1.91E-04
3:68934434-SNV	rs4855535	TAFA4	5_prime_UTR_variant	Other	G	T					CREB3L2	Gain	5.644	2.17E-04
3:68934434-SNV	rs4855535	TAFA4	5_prime_UTR_variant	Other	G	T					HSFY2	loss	-6.206	2.68E-04
3:68934434-SNV	rs4855535	TAFA4	5_prime_UTR_variant	Other	G	T					BRCA1	Gain	4.377	6.52E-04
3:68934434-SNV	rs4855535	TAFA4	5_prime_UTR_variant	Other	G	T					NFE2	Gain	2.53	9.05E-04
5:7851181-SNV	rs161874	C5orf49	5_prime_UTR_variant	Other	A	C					E2F1	loss	-3.404	1.51E-05
5:7851181-SNV	rs161874	C5orf49	5_prime_UTR_variant	Other	A	C					PAX4	loss	-6.859	3.62E-05
5:7851181-SNV	rs161874	C5orf49	5_prime_UTR_variant	Other	A	C					TEAD2	loss	-3.336	7.56E-05
5:7851181-SNV	rs161874	C5orf49	5_prime_UTR_variant	Other	A	C					SREBF	Gain	3.553	1.36E-04
1:116235183-Del	rs3841008	VANGL1	3_prime_UTR_variant	Other	-	A	hsa-miR-6734-3p	Gain	22.54	0.04				
1:116235183-Del	rs3841008	VANGL1	3_prime_UTR_variant	Other	-	A	hsa-miR-320a-5p	loss	20.5	0.02				
1:116235183-Del	rs3841008	VANGL1	3_prime_UTR_variant	Other	-	A	hsa-miR-629-3p	loss	21.5	0.03				
10:97366107-SNV	rs8758	ALDH18A1	3_prime_UTR_variant	Other	G	A	hsa-miR-509-3-5p	Gain	22.65	0.01	ATF3	Gain	3.611	4.38E-04
10:97366107-SNV	rs8758	ALDH18A1	3_prime_UTR_variant	Other	G	A	hsa-miR-4418	Gain	22.65	0.01	ELK4	Gain	1.966	1.01E-03
10:97366107-SNV	rs8758	ALDH18A1	3_prime_UTR_variant	Other	G	A	hsa-miR-509-5p	Gain	22.65	0.01	SIRT6	Gain	4.721	1.22E-03
10:97366107-SNV	rs8758	ALDH18A1	3_prime_UTR_variant	Other	G	A	hsa-miR-20a-3p	Gain	23.73	0.01	FOXO6	loss	-3.476	1.96E-03
10:97366107-SNV	rs8758	ALDH18A1	3_prime_UTR_variant	Other	G	A	hsa-miR-5002-3p	Gain	23.73	0.01	ELF1	Gain	2.427	1.99E-03
10:97366107-SNV	rs8758	ALDH18A1	3_prime_UTR_variant	Other	G	A	hsa-miR-5011-3p	Gain	23.15	0.02	IRX4	loss	-2.901	3.09E-03
10:97366107-SNV	rs8758	ALDH18A1	3_prime_UTR_variant	Other	G	A					SMARC	loss	-2.838	3.57E-03
10:97366107-SNV	rs8758	ALDH18A1	3_prime_UTR_variant	Other	G	A					EGR1	Gain	2.483	3.69E-03
2:219501328-SNV	rs1055816	PLCD4,ZNF142	3_prime_UTR_variant	Other	A	G	hsa-miR-7157-5p	Gain	20.39	0.01	GLI	loss	-3.286	3.98E-04
2:219501328-SNV	rs1055816	PLCD4,ZNF142	3_prime_UTR_variant	Other	A	G	hsa-miR-4310	Gain	22.39	0.01	TEAD3	Gain	4.627	6.61E-04
2:219501328-SNV	rs1055816	PLCD4,ZNF142	3_prime_UTR_variant	Other	A	G	hsa-miR-1228-3p	loss	22.93	0.03	TATA	Gain	3.46	1.05E-03
2:219501328-SNV	rs1055816	PLCD4,ZNF142	3_prime_UTR_variant	Other	A	G	hsa-miR-8064	loss	23	0.01	FOXK1	loss	-3.71	1.13E-03
2:219501328-SNV	rs1055816	PLCD4,ZNF142	3_prime_UTR_variant	Other	A	G	hsa-miR-383-3p	loss	21.39	0.01	HIC1	Gain	3.164	1.41E-03
2:219501328-SNV	rs1055816	PLCD4,ZNF142	3_prime_UTR_variant	Other	A	G	hsa-miR-3065-3p	loss	21.39	0.01	TBX20	loss	-2.68	1.59E-03
2:219501328-SNV	rs1055816	PLCD4,ZNF142	3_prime_UTR_variant	Other	A	G					REST	loss	-3.79	1.65E-03
2:219501328-SNV	rs1055816	PLCD4,ZNF142	3_prime_UTR_variant	Other	A	G					HNF4A	loss	-3.401	2.20E-03
6:160888506-Ins	rs746085166	LPAL2	non_coding_exon_variant	Other	GT	-					AP1	loss	-2.85	9.53E-05
6:160888506-Ins	rs746085166	LPAL2	non_coding_exon_variant	Other	GT	-					PAX4	Gain	6.12	1.56E-04
6:160888506-Ins	rs746085166	LPAL2	non_coding_exon_variant	Other	GT	-					GATA4	Gain	2.44	2.97E-03
6:160888506-Ins	rs746085166	LPAL2	non_coding_exon_variant	Other	GT	-					SIN3A	Gain	2.898	3.08E-03
6:160888506-Ins	rs746085166	LPAL2	non_coding_exon_variant	Other	GT	-					DMRTC2	loss	-3.343	4.32E-03
6:160888506-Ins	rs746085166	LPAL2	non_coding_exon_variant	Other	GT	-					FOXP1	loss	-1.973	4.56E-03
6:160888506-Ins	rs746085166	LPAL2	non_coding_exon_variant	Other	GT	-					PTF1A	Gain	2.114	4.95E-03
6:160888506-Ins	rs746085166	LPAL2	non_coding_exon_variant	Other	GT	-					MYEF2	loss	-1.604	5.18E-03

15:55881107-SNV	rs1992237	PYGO1	5_prime_UTR_variant	Other	T	C					FOXL1	Gain	3.848	7.45E-05
15:55881107-SNV	rs1992237	PYGO1	5_prime_UTR_variant	Other	T	C					SCRT1	Gain	4.403	1.20E-04
15:55881107-SNV	rs1992237	PYGO1	5_prime_UTR_variant	Other	T	C					MEIS2	loss	-3.643	2.01E-04
15:55881107-SNV	rs1992237	PYGO1	5_prime_UTR_variant	Other	T	C					HLF	Gain	2.844	8.76E-04
15:55881107-SNV	rs1992237	PYGO1	5_prime_UTR_variant	Other	T	C					HDAC2	Gain	3.107	9.71E-04
5:148376982-SNV	rs1347130	SH3TC2	3_prime_UTR_variant	Other	A	G					SIX5	loss	-4.583	9.72E-05
5:148376982-SNV	rs1347130	SH3TC2	3_prime_UTR_variant	Other	A	G					NFKB	Gain	3.009	1.92E-03
5:148376982-SNV	rs1347130	SH3TC2	3_prime_UTR_variant	Other	A	G					TRIM28	loss	-2.494	2.05E-03
5:148376982-SNV	rs1347130	SH3TC2	3_prime_UTR_variant	Other	A	G					FOXO3	Gain	3.062	2.99E-03
5:148376982-SNV	rs1347130	SH3TC2	3_prime_UTR_variant	Other	A	G					RUNX2	loss	-2.551	3.07E-03
5:148376982-SNV	rs1347130	SH3TC2	3_prime_UTR_variant	Other	A	G					TCF12	loss	-1.95	3.60E-03
5:148376982-SNV	rs1347130	SH3TC2	3_prime_UTR_variant	Other	A	G					CACBP	loss	-1.948	3.69E-03
11:104896844-SNV	rs1977989	CASP1	3_prime_UTR_variant	Other	G	A					POU3F3	loss	-3.997	1.32E-04
11:104896844-SNV	rs1977989	CASP1	3_prime_UTR_variant	Other	G	A					RXRA	loss	-4.635	1.06E-03
11:104896844-SNV	rs1977989	CASP1	3_prime_UTR_variant	Other	G	A					HMGA1	loss	-3.834	1.24E-03
11:104896844-SNV	rs1977989	CASP1	3_prime_UTR_variant	Other	G	A					NANOG	loss	-4.473	2.16E-03
11:104896844-SNV	rs1977989	CASP1	3_prime_UTR_variant	Other	G	A					HSFY2	Gain	4.237	2.63E-03
12:1021159-SNV	rs10849584	RAD52	3_prime_UTR_variant	Other	T	C					PPARA	Gain	12.985	3.60E-07
12:1021159-SNV	rs10849584	RAD52	3_prime_UTR_variant	Other	T	C					MYC	loss	-13.258	3.34E-06
12:1021159-SNV	rs10849584	RAD52	3_prime_UTR_variant	Other	T	C					ETS	loss	-11.02	3.72E-06
12:1021159-SNV	rs10849584	RAD52	3_prime_UTR_variant	Other	T	C					PAX4	loss	-8.394	4.64E-06
12:1021159-SNV	rs10849584	RAD52	3_prime_UTR_variant	Other	T	C					AP1	loss	-8.993	1.76E-05
12:1021159-SNV	rs10849584	RAD52	3_prime_UTR_variant	Other	T	C					REST	loss	-6.681	3.68E-05
12:1021159-SNV	rs10849584	RAD52	3_prime_UTR_variant	Other	T	C					BCL	loss	-5.746	1.13E-04
19:10370542-SNV	rs11115	MRPL4	3_prime_UTR_variant	Other	T	C	hsa-miR-3188	loss	21.22	0.05	BDP1	loss	-3.094	2.24E-05
19:10370542-SNV	rs11115	MRPL4	3_prime_UTR_variant	Other	T	C	hsa-miR-4324	loss	23.25	0.03	AP1	loss	-2.729	1.14E-03
19:10370542-SNV	rs11115	MRPL4	3_prime_UTR_variant	Other	T	C	hsa-miR-3975	loss	19.72	0.03				
19:10370542-SNV	rs11115	MRPL4	3_prime_UTR_variant	Other	T	C	hsa-miR-6765-5p	loss	21.69	0.02				
19:10370542-SNV	rs11115	MRPL4	3_prime_UTR_variant	Other	T	C	hsa-miR-4649-3p	loss	22.32	0.01				
19:10370542-SNV	rs11115	MRPL4	3_prime_UTR_variant	Other	T	C	hsa-miR-2467-5p	loss	20.71	0.02				
19:10370542-SNV	rs11115	MRPL4	3_prime_UTR_variant	Other	T	C	hsa-miR-544b	loss	23.24	0.02				
19:10370542-SNV	rs11115	MRPL4	3_prime_UTR_variant	Other	T	C	hsa-miR-485-5p	loss	19.72	0.05				
19:10370542-SNV	rs11115	MRPL4	3_prime_UTR_variant	Other	T	C	hsa-miR-6884-5p	loss	20.22	0.05				

Supplementary Table 5:

Details of the selected variants detected in the tumors that create binding sites of miRNAs and transcription factors (TFs) influencing gain or loss of function of the targeted gene (shown separately in Whites, Blacks, and Native Americans).
